# Solid-Phase Parallel Synthesis of Dual Histone Deacetylase-Cyclooxygenase Inhibitors

**DOI:** 10.3390/molecules28031061

**Published:** 2023-01-20

**Authors:** Luisa M. Bachmann, Maria Hanl, Felix Feller, Laura Sinatra, Andrea Schöler, Jens Pietzsch, Markus Laube, Finn K. Hansen

**Affiliations:** 1 Institute for Drug Discovery, Medical Faculty, Leipzig University, Brüderstraße 34, 04103 Leipzig, Germany; 2 Department of Pharmaceutical and Cell Biological Chemistry, Pharmaceutical Institute, University of Bonn, An der Immenburg 4, 53121 Bonn, Germany; 3 Department of Radiopharmaceutical and Chemical Biology, Institute of Radiopharmaceutical Cancer Research, Helmholtz-Zentrum Dresden-Rossendorf, Bautzner Landstraße 400, 01328 Dresden, Germany; 4 Faculty of Chemistry and Food Chemistry, School of Science, Technische Universität Dresden, Mommsenstraße 4, 01062 Dresden, Germany

**Keywords:** cancer, COX, HDAC, multi-target drugs, solid-phase synthesis

## Abstract

Multi-target drugs (MTDs) are emerging alternatives to combination therapies. Since both histone deacetylases (HDACs) and cyclooxygenase-2 (COX-2) are known to be overexpressed in several cancer types, we herein report the design, synthesis, and biological evaluation of a library of dual HDAC-COX inhibitors. The designed compounds were synthesized via an efficient parallel synthesis approach using preloaded solid-phase resins. Biological in vitro assays demonstrated that several of the synthesized compounds possess pronounced inhibitory activities against HDAC and COX isoforms. The membrane permeability and inhibition of cellular HDAC activity of selected compounds were confirmed by whole-cell HDAC inhibition assays and immunoblot experiments. The most promising dual inhibitors, **C3** and **C4**, evoked antiproliferative effects in the low micromolar concentration range and caused a significant increase in apoptotic cells. In contrast to previous reports, the simultaneous inhibition of HDAC and COX activity by dual HDAC-COX inhibitors or combination treatments with vorinostat and celecoxib did not result in additive or synergistic anticancer activities.

## 1. Introduction

Every year, several millions of people die of cancer [1]. The development and investigation of new anticancer drugs therefore remains an important challenge. As a multifactorial disease with many signaling pathways and various dysregulated physiological processes involved, cancer requires complex therapy regimes that modulate different biological targets [2,3,4]. In addition to the well-established strategy of combination therapies, multi-target drugs (MTDs), have gained growing attention over the last years [5,6]. In combination therapy, a minimum of two drugs is applied to achieve synergistic or additive effects aiming at a higher therapeutic efficacy. Engaging different modes of action, they may also help to avoid cellular mechanisms of compensation or resistance [3,5]. MTDs, in contrast, are single molecules with multiple modes of action that specifically address several disease-relevant targets [2,5]. The advantages of MTDs include the simplification of therapy regimes and the reduction of side effects and drug-drug-interactions [3,4,5]. Another important advantage of MTDs is that only one regulatory approval is required for one molecular entity, while two or more approvals are needed for a combination therapy. The challenge in designing MTDs lies in the balancing of the affinity to each target while retaining suitable physicochemical properties and the desired pharmacological activities. Optimal efficacy may, moreover, require different doses reaching the respective targets [2,3,4,5].

In recent years, various target combinations have been covered for potent hybrid molecules inspired by different drug classes [2,7,8,9,10,11]. In this study, we focused on the development of dual inhibitors of histone deacetylases (HDACs) and cyclooxygenase-2 (COX-2), both of which are known to be overexpressed in several cancer types [7,12,13,14,15,16].

In brief, HDACs modify histones by removing acetyl groups from ε-*N*-acetylated lysine residues to induce the formation of a condensed chromatin structure which is inaccessible for the transcription of DNA [2,8,17,18,19]. Thus, HDAC inhibition appears to be a promising strategy to treat cancer by regulating the activation of tumor-suppressing genes or the deactivation of tumor-promoting genes, respectively [8,17,18]. Involved in cell growth, proliferation, differentiation, migration, angiogenesis, immunomodulation, and other cellular processes [20]; eighteen different HDAC isoforms are known so far. Generally, they are divided into zinc-dependent enzymes (HDAC1-11; class I: HDAC1-3 and 8; class IIa: HDAC4, 5, 7 and 9; class IIb: HDAC6 and 10; class IV: HDAC11) and NAD^+^-dependent sirtuins (SIRT1-7; class III) [2,19]. In accordance with the conserved structure of the binding site, hybrid HDAC inhibitors typically feature the characteristic linker and the zinc binding group (ZBG) of HDAC inhibitors (HDACi) and solvent exposed cap groups derived from other cytotoxic or targeted anticancer drugs [2,7,8]. 

Functioning as a oxidoreductase, COX-2 converts arachidonic acid to prostaglandin H2 [21]. COX-2 is inducible by cytokines, tumor promotors, and growth factors, whereas constitutive expression is limited to kidneys, brain, the gastro-intestinal tract, and thymus [21,22,23]. Often overexpressed in tumors, COX-2 is involved in the regulation of cell proliferation, migration, angiogenesis, apoptosis, metastasis, and immune resistance of tumor cells [12,13,14,24,25]. Different studies reported that the reduction in prostaglandin E2 synthesis in tumors by non-steroidal anti-inflammatory drugs (NSAIDs) or COX-2 inhibitors is associated with chemo-preventive effects in some tumors, such as colon, breast, prostate, lung, head, and neck cancer. COX-2 inhibition sensitizes cancer cells to chemotherapeutics, reduces the risk of metastasis and showed synergistic effects in combination with chemotherapy and radiotherapy in animal experiments. Furthermore, the tumor mortality and the probability for recurrence may be reduced [12,14,26,27,28,29,30,31].

The simultaneous inhibition of COX-2 and HDACs by means of combination therapies showed synergistic antitumor effects on cells derived from salivary adenoid cystic carcinoma as well as tongue squamous cell carcinoma and appeared more effective than single treatment with either drug [32,33]. Another study indicated potent cell growth inhibition by dual COX-2/HDAC inhibition in a human pancreatic ductal adenocarcinoma (PDAC) mimicking cell model [34]. Being a very deadly type of cancer that is often diagnosed in a late stage, PDAC is as yet poorly treatable, but there is some evidence suggesting that HDAC expression is altered in PDAC while deregulated histone acetylation seems to drive the development of pancreatic cancer [34]. HDACi monotherapy, however, indicated limited efficacy in PDAC and other solid tumors [34]. In a study on lung cancer cells, it was observed that HDAC inhibition induces COX-2 expression which, in turn, stimulates the tumor growth, thus possibly causing the limited efficacy [34]. A combination of both COX-2 and HDAC inhibition therefore seems to be a promising treatment option.

A previous study on dual-acting COX-2/HDAC inhibitors as MTDs based on celecoxib and indometacin as COX-2 binding motif and a hydroxamic acid moiety as HDAC binding motif demonstrated cell growth inhibition in different tumor cell lines (breast, non-small cell lung, colon, androgen-dependent and -independent prostate cancer) that outmatched combinations of vorinostat with either celecoxib or indometacin [35]. In healthy cells, the damage was less significant [35].

In this study, we present hybrid molecules with inhibitory activities against COX-2 and selected HDAC isoforms. The synthesized conjugates consist of a hydroxamic acid function as ZBG, a variable linker region, and different cap groups. The linker fragments were attached to a ZBG precursor, which was immobilized on a solid phase. Subsequent coupling of the COX inhibitory cap groups followed by cleavage from the solid phase yielded a range of bifunctional inhibitors. All synthesized compounds were tested for their inhibitory activity against COX-1, COX-2, HDAC1, and HDAC6 as well as their antiproliferative activity against PDAC cell line AsPC1. The most promising dual inhibitors were further characterized in a panel of seven cancer cell lines. Furthermore, we investigated the intracellular target engagement of the best compounds by whole-cell HDAC inhibition assays and immunoblot experiments. Their ability to induce apoptosis was confirmed by annexin V/propidium iodide apoptosis assays.

## 2. Results and Discussion

### 2.1. Design and Synthesis of Dual HDAC-COX Inhibitors

**Scheme 1:**
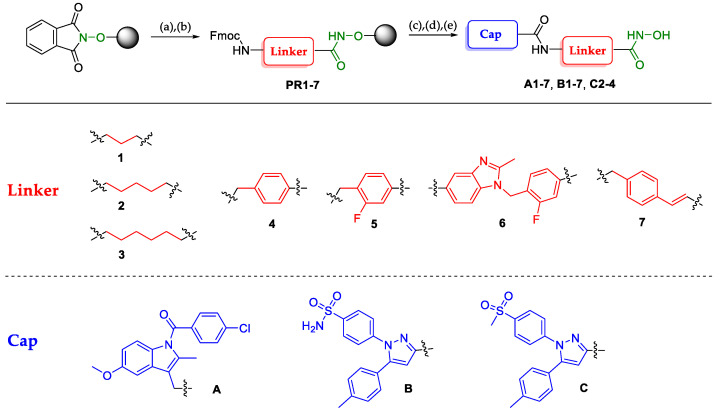
Solid phase synthesis of dual HDAC-COX inhibitors. Reagents and conditions: (a) 5% N_2_H_4_*H_2_O in MeOH, r.t., 2 × 15 min. (b) Fmoc-NH-Linker-COOH (2.00 eq.), HATU (2.00 eq.), HOBt*H_2_O (2.00 eq.), DIPEA (3.00 eq.) in DMF, r.t., 18 h. (c) 20% piperidine in DMF, r.t., 2 × 5 min. (d) Cap-COOH (2.00 eq.), HATU (2.00 eq.), DIPEA (3.00 eq.) in DMF, r.t., 18 h. (e) 5% TFA in DCM, r.t., 1 h.

For the design of the target compounds, we selected seven different HDACi linkers including alkyl linkers (**1–3**), benzyl linkers (**4**, **5**), a benzimidazole-fluorobenzyl linker (**6**), and a phenylvinyl linker (**7**) (Scheme 1). Comprising a propyl linker that is too short to reach the Zn^2+^ in the active site, linker **1** was included as an HDAC negative control. The alkyl linkers **2** and **3** as well as the phenylvinyl linker **7** were predicted to yield rather unselective HDACi due to their similarity to the pan-HDACi vorinostat and panobinostat. In contrast, the benzyl linkers **4**, **5,** and the benzimidazole-fluorobenzyl linker **6,** were included as well-known HDAC6 preferential linkers [36,37,38]. For the COX inhibitor scaffold (and HDACi capping group), we selected the unselective COX inhibitor indometacin (**A**) and two COX-2 selective celecoxib-based derivatives (**B**, **C**). With **B** containing a sulfonamide in *p*-position of one of the phenyl rings and **C** featuring a methyl sulfone moiety (see Scheme 1), the two compounds differ by only one functional group. 

The designed compounds were synthesized as outlined in Scheme 1. In the first step, a phthaloyl protected hydroxylamine resin, readily available from *N*-hydroxyphthalimide and a 2-chlorotrityl chloride (2-CTC) resin [39], was deprotected by treatment with hydrazine hydrate to furnish the free aminoxy group. The subsequent amide coupling reaction with the respective Fmoc-protected linker generated the preloaded resins **PR1-7** in multi-gram scales. The seven preloaded resins were obtained in high purities (as indicated by test cleavages followed by purity determination by high performance liquid chromatography (HPLC)) and in loading capacities ranging from 0.48 to 0.77 mmol*g^−1^. For the subsequent parallel synthesis of the desired dual HDAC-COX inhibitors, an aliquot of the respective resin (**PR1-7**) was subjected to Fmoc deprotection followed by amide coupling reaction with the cap group **A**, **B**, or **C**. Cleavage from the solid support followed by purification with preparative HPLC yielded the target compounds **A1–A7**, **B1–B7**, and **C2–C4** (Table 1). Compounds **A6** and **B6** were isolated as TFA salts due to the acidic modifier during HPLC purification. Compounds **A1**, **A4–A7**, **B1**, **B4–B7**, **C2**, and **C4** are novel compounds, while compounds **A2, A3**, **B2, B3**, and **C3** were previously prepared by Raji et al. using solution-phase methods [35].

### 2.2. Determination of COX Inhibition

In general, cyclooxygenase inhibitors exert anti-inflammatory, antipyretic, and analgesic effects by binding in the cyclooxygenase-active site and hence inhibiting the synthesis of prostaglandins [21]. COX-2 inhibitors, also called coxibs, selectively bind to the COX-2 isoform whose binding pocket differs from COX-1 because of an amino acid exchange in the sequence from isoleucine to valine, which facilitates the binding of taller substrates [21]. As lead compounds of this study, indometacin happens to be unselective, whereas celecoxib represents a selective inhibitor for the isoenzyme. The ability to inhibit both isoforms, ovine COX-1 and human COX-2, was determined using the “COX Fluorescent Inhibitor Screening Assay Kit” (Cayman Chemical, Ann Arbor, MI, USA) in a concentration range up to 100 µM [40,41]; the results are shown in Table 2. In general, modification of indometacin (**A**) and celecoxib derivatives (**B** and **C**) with linker and zinc binding motifs was tolerated by COX-2 leading to inhibitory potencies in the low micromolar range of IC_50_ between 1–10 µM with exception of compound **B1** (IC_50_ = 43.0 µM). More pronounced differences were obtained in the selectivity between both isoforms due to differences in the inhibition profile for COX-1 ranging from IC_50_ of 1.8 µM up to >100 µM. A definite structure-activity relationship could not be derived from this set of substances. The highest selectivity and most potent COX-2 inhibition was obtained for the methylsulfonyl-substituted celecoxib derivative **C3** comprising a hexyl linker which showed a 96-fold selectivity for COX-2 and IC_50_ (COX-2) of 0.98 µM. The respective aminosulfonyl-substituted celecoxib derivative **B3** showed unselective inhibition, while the indometacin derivative **A3** demonstrated a 14-fold preference for COX-2. The COX-2-selective inhibition for the alkyl substituted derivatives **A2**, **A3**, **B2**, **B3**, and **C3** reported by Raji et al. [35] could be verified in our study, although the observed trend was not completely in line with our results. Compound **A3**, which Raji et al. found to be a very potent and selective inhibitor of COX-2 (IC_50_ COX-2 = 0.33 µM and IC_50_ COX-1 = 21.00 µM [35]), appeared to be no more than a moderate inhibitor in this set. Compound **C3** (IC_50_ COX-2 = 4.44 µM and IC_50_ COX-1 = 23.09 µM [35]), on the other hand, showed opposite results. For the aromatic linker derivatives **4–7** which were designed for this study, we found rather non-selective COX inhibition in the low micromolar concentration range. The two benzimidazole-fluorobenzyl-based compounds **A6** and **B6** exerted nearly equipotent inhibition of both isoforms, while the derivatives with benzyl linkers **4** and **5**, as well as the phenylvinyl linker **7** favored COX-2 inhibition with selectivity indices ranging between 2–15.

### 2.3. Inhibition of HDAC1 and HDAC6 

All synthesized compounds and vorinostat as positive control were screened in a fluorogenic assay for their in vitro inhibitory activity against HDAC1 (as a representative class I isoform) and HDAC6 (as a representative class IIb isoform); the results are presented in Table 2. As expected, the compounds with a short propyl linker (**A1** and **B1**) showed low inhibitory activities against both isoforms. In agreement with the literature data on vorinostat and panobinostat derivatives [42], the compounds with a pentyl (**A2**, **B2**, and **C2**), hexyl (**A3**, **B3**, and **C3**), and phenylvinyl linker (**A7** and **B7**) turned out to be potent but rather unselective HDACi. The introduction of a benzyl linker provided compounds **A4**, **B4**, and **C4** with a slightly improved preference for HDAC6. This effect was more pronounced when a fluorine atom was introduced in *meta*-position to the hydroxamic acid (**A5** and **B5**). The beneficial effect of a fluorinated benzyl linker with regard to HDAC6 selectivity is in line with recent results disclosed by us and others [38,43,44]. Notably, the benzimidazole-fluorobenzyl-based compounds **A6** (IC_50_ HDAC1 = >2.80 µM; IC_50_ HDAC6 = 0.011 µM; SI = >254) and **B6** (IC_50_ HDAC1 = 0.829 µM; IC_50_ HDAC6 = 0.005 µM; SI = 166) demonstrated potent and highly selective HDAC6 inhibition, which is in excellent agreement with a recent report on HDAC6-selective proteolysis-targeting chimeras (PROTACs) [45]). With respect to the impact of the different cap groups, it can be concluded that the indometacin-derived derivatives (**A** series) are suitable for less potent but more selective HDAC6 inhibition. In contrast, the celecoxib-based compounds (**B** and **C** series) displayed more potent but less selective HDAC6 inhibition.

### 2.4. Determination of Lipophilicity

As a measure of lipophilicity, log*D*_7.4HPLC_ as the distribution coefficient at pH 7.4 was determined for all compounds (Table 2) by an HPLC method originally described by Donovan and Pescatore [46]. The log*D*_7.4HPLC_ was found to consistently increase with the respective linker lipophilicity in each sub-set (**A**, **B**, or **C**) of compounds. Covering a range from 1.23 for **B1** to 3.31 for **A7**, the compound lipophilicities lie within the range predicted for potential good absorption and bioavailability in vivo and moderate permeability and solubility in general [47].

### 2.5. Antiproliferative Properties of Dual HDAC-COX Inhibitors

The antiproliferative potential of the dual HDAC-COX inhibitors **A1–A7**, **B1–B7**, and **C2–C4** was determined using MTT assays in the PDAC cell line AsPC1. The PDAC cell line was chosen due to a report by Peulen et al., in which it was demonstrated that the anti-cancer effects of HDACi can be enhanced by the simultaneous inhibition of COX-2 [34]. Vorinostat and celecoxib were included as control compounds; the results are summarized in Table 2. Five out of the 17 tested dual inhibitors showed no effect against the AsPC1 cell line. The remaining compounds revealed IC_50_ values ranging from 2.39 to 59.32 µM. Vorinostat (IC_50_ AsPC1 = 1.04 µM) turned out to be the most active compound in this screening, while celecoxib displayed no antiproliferative activity.

Compounds **A7**, **B7**, **C3**, and **C4** were selected as representative compounds to assess their antiproliferative potential in a larger panel of cell lines including melanoma (MV3), pancreatic cancer (PC-3), breast cancer (MDA-MB-231), squamous cell carcinoma (FaDu), colorectal cancer (HT-29), and glioblastoma (U-87). This initial selection of candidates for a more detailed analysis in different cell lines was based both on lipophilicity and cytotoxic effects in the AsPC1 cell line. Based on the IC_50_ values from the COX and HDAC inhibition assays in comparison to the effects on AsPC1 cells, we concluded that some of the compounds were potent inhibitors against the isolated enzymes but not able to enter cells and exert their effect there. **B4**, for example, was one very potent HDAC inhibitor in the low nanomolar range which showed, however, only a IC_50_ of 15.84 µM against AsPC1. The selected derivatives (**A7**, **B7**, **C3**, and **C4**) cover compounds from all three classes (A, B, and C) with different lipophilicity and compounds that exerted cell cytotoxicity in the potent, intermediate, and weak range. **C3** and **C4** were selected as very potent inhibitors in AsPC1 cell viability having a low lipophilicity, **B7** as a potent inhibitor in AsPC1 cells with intermediate lipophilicity, and **A7** as rather weak inhibitor in AsPC1 cells having the highest lipophilicity. Again, vorinostat and celecoxib were included as controls; the results are presented in Table 3. Interestingly, the dual HDAC-COX inhibitor **C3** exerted the most pronounced antiproliferative activities across the seven cell lines with IC_50_ values ranging from 2.21 µM (FaDu) to 6.91 µM (U-87). Vorinostat displayed the highest activity in all cell lines, while celecoxib was inactive. To investigate the potential of the combined inhibition of HDAC and COX activity by single target drugs, we also included 1:1 combinations of vorinostat and celecoxib into the assays. The combinations showed similar IC_50_ values compared to the treatment with vorinostat alone (Table 3), thus indicating that the activity of vorinostat is not boosted by celecoxib. On the whole, our data indicate that, at least in the seven cell lines used in this study, the simultaneous inhibition of HDAC and COX activity by dual HDAC-COX inhibitors or a combination treatment does not lead to additive or synergistic anticancer activity. 

### 2.6. Intracellular Target Engagement and Apoptosis Induction

**Figure 1:**
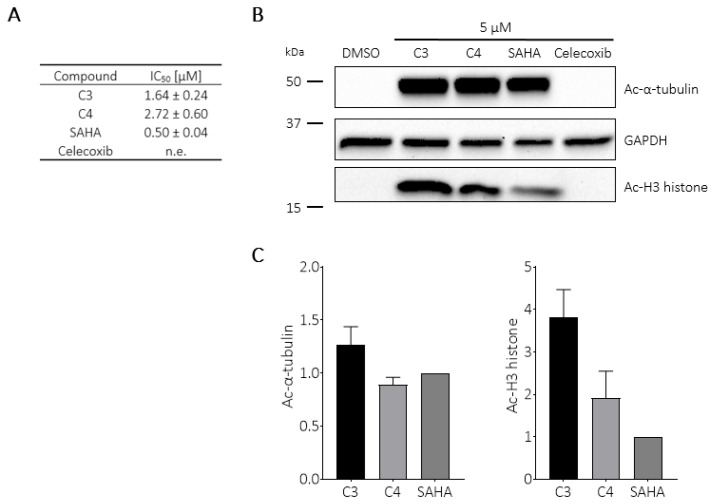
Intracellular target engagement of histone deacetylases by compounds **C3** and **C4**. (**A**)**.** Whole-cell HDAC inhibition assays of **C3**, **C4**, vorinostat, and celecoxib in PC-3 cells; n.e. = no effect up to 25 µM. (**B**)**.** Representative immunoblot analysis of acetylated α-tubulin and acetylated histone H3. PC-3 cells were incubated for 24 h with **C3**, **C4**, vorinostat, and celecoxib at the indicated concentration. Afterwards, cell lysates were immunoblotted with anti-acetyl-α-tubulin and acetyl-histone H3 antibodies. GAPDH was used as a loading control. (**C**)**.** Densitometric analysis of acetylated α-tubulin and acetylated histone H3 of **C3**, **C4** and vorinostat. Intensity was normalized to GAPDH and depictured in relation to vorinostat.

On average across seven cell lines, compounds **C3** and **C4** demonstrated the most pronounced antiproliferative activities. Consequently, both compounds were characterized in more detail using vorinostat and celecoxib as controls. First, the cellular HDAC inhibitory activities of the selected compounds were assessed in whole-cell HDAC inhibition assays in PC-3 cells using Boc-Lys(Ac)-AMC as broad-spectrum HDAC substrate; the results are summarized in Figure 1A. In good agreement with the results from the MTT assays (Table 3), compound **C3** (IC_50_ = 1.64 µM) displayed a more potent inhibition of cellular HDAC activity compared to **C4** (IC_50_ = 2.72 µM). The positive control vorinostat showed the strongest HDAC inhibitory capacity (IC_50_ = 0.50 µM), while, as expected, celecoxib had no effect on HDAC activity up to a concentration of 100 µM. To confirm the HDAC inhibition in a cellular environment, PC-3 cells were treated with 5 µM of **C3**, **C4**, vorinostat, and celecoxib and subsequently subjected to immunoblot analysis. **C3**, **C4**, and vorinostat induced prominent acetylation of histone H3 (a marker of HDAC1-3 inhibition) and α-tubulin (a marker of HDAC6 inhibition), while celecoxib did not increase the acetylation levels of both proteins (Figure 1B). Taken together, the results from the whole-cell HDAC inhibition assays and immunoblot experiments confirmed the intracellular unselective HDAC inhibition by **C3** and **C4**. 

**Figure 2:**
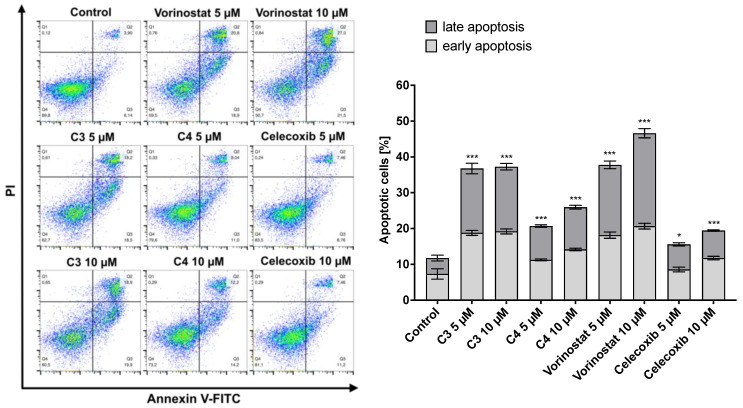
Apoptosis of PC-3 cells induced by **C3**, **C4**, vorinostat, and celecoxib. **C3**, **C4**, vorinostat, and celecoxib were incubated with PC-3 cells for 72 h at the indicated concentrations and apoptosis was detected by annexin V and PI staining; representative data of *n* = 2 independent experiments performed in triplicates. Statistical analysis to compare annexin V positive cells with control was performed using one-way ANOVA (* *p* < 0.05, *** *p* < 0.001).

In the next step, the induction of apoptosis following the treatment of PC-3 cells with 5 or 10 µM of **C3**, **C4**, vorinostat, and celecoxib for 72 h was determined (Figure 2). The treated cells were stained with annexin V and propidium iodide (PI) and afterwards analyzed by flow cytometry. The percentage of cells that were annexin V positive but PI negative were considered early apoptotic, and the percentage of cells that were both annexin V and PI positive were considered late apoptotic. The treatment with **C3** and **C4** (5 and 10 µM) led to a significant (*p* < 0.001) increase in apoptotic cells. These results indicate that the induction of apoptosis contributes to the antiproliferative properties of **C3** and **C4**.

## 3. Materials and Methods

### 3.1. General Remarks

#### 3.1.1. Materials and Experimental Procedures

Commercially available chemicals were purchased from abcr GmbH (Karlsruhe, Germany), Acros Organics (Thermo Fisher Scientific, Geel, Belgium), Alfa Aesar (Thermo Fisher Scientific, Kandel, Germany), Carbolution Chemicals GmbH (St. Ingbert, Germany), Carl Roth GmbH + Co. KG (Karlsruhe, Germany), Fluorochem (Hadfield, United Kingdom), Grüssing (Filsum, Germany), Iris Biotech GmbH (Marktredwitz, Germany), Merck KGaA (Darmstadt, Germany), Sigma-Aldrich (St. Louis, MO, USA), TCI Deutschland GmbH (Eschborn, Germany), and VWR International (Radnor, PA, USA) and used without further purification. The cap groups 1-(4-sulfamoylphenyl)-5-(*p*-tolyl)-1*H*-pyrazole-3-carboxylic acid and 1-(4-(methylsulfonyl)phenyl)-5-(*p*-tolyl)-1*H*-pyrazole-3-carboxylic acid were prepared as previously reported [41,48]. Methyl 3-fluoro-4-methylbenzoate and methyl 4-(bromomethyl)-3-fluorobenzoate, for the synthesis of the fluorobenzyl linker, were synthesized according to the literature [45]. The Fmoc-protected propyl, hexyl, benzyl, benzimidazole-fluorobenzyl and phenylvinyl linkers were synthesized as previously described [39,45]. The Fmoc-protection of the linkers, the modification of the 2-chlorotrityl chloride resin, and the coupling of the linkers on the modified resin were also performed as previously reported [39]. Solvents of technical-grade quality were distilled before use. Except of peptide grade DMF (Iris Biotech GmbH, Marktredwitz, Germany), HPLC-grade solvents were used for the solid-phase synthesis. For analytical and preparative HPLC, ACN in HPLC-grade quality (HiPerSolv CHROMANORM, VWR) was used. Bidistilled water was obtained from distilled water with a Milli-Q Simplicity 185 Water Purification System (Merck Millipore (Burlington, MA, USA)). For the solid-phase synthesis PP-syringes with PE-frits (sizes: 2/10/20 mL, pore size: 23 μm, MultiSynTech GmbH (Witten, Germany)) were used. The 2-chlorotrityl chloride resin (200–400 mesh, 1.60 mmol/g) was purchased from Iris Biotech. Thin-layer chromatography (TLC) was performed on silica plates (silica gel 60, F254, Merck) and ultraviolet light (254 nm) was used for the detection. Column chromatography was carried out on silica gel (NORMASIL 60^®^, particle size: 40–63 µm, VWR). 

#### 3.1.2. High Performance Liquid Chromatography (HPLC) 

For analytical purposes, a Thermo Fisher Scientific GmbH (Waltham, MA, USA) UltiMate^TM^ 3000 UHPLC system with a Macherey Nagel (Düren, Germany) Nucleodur 5 μm C18 100 Å column (250 × 4.6 mm) was used with bidistilled H_2_O with 0.1% TFA (A) and ACN (B) as mobile phase. After the column was equilibrated for 5 min with the initial conditions A/B 95:5, a linear gradient from 5 to 95% B over 15 min was performed followed by a 5 min isocratic phase of 95% B. The method was carried out with a flow rate of 1 mL/min at 25 °C and the chromatograms were acquired via UV absorption detection at 254 nm.

For the preparative purification a Varian ProStar HPLC system, either a preparative Macherey Nagel Nucleodur 5 μm C18 HTec column (150 × 32 mm) or a semi-preparative Phenomenex (Aschaffenburg, Germany) Jupiter 5 μm C18 100 Å column (250 × 10 mm) was used with bidistilled H_2_O with 0.1% TFA (A) and ACN (B) as mobile phase. After the column was equilibrated for 5 min with the initial conditions A/B 95:5, a linear gradient from 5 to 95% B over 15 min was performed followed by a 10 min isocratic phase of 95% B (method A). For method B, the linear gradient from 5 to 95% B was extended to 20 min. The methods were carried out with flow rates of 15 mL/min for the preparative and 4 mL/min for the semi-preparative column at room temperature and the chromatograms were acquired via UV absorption detection at 220 and 254 nm.

Furthermore, a Knauer (Berlin, Germany) AZURA preparative HPLC system with a preparative Macherey Nagel Nucleodur 5 μm C18 HTec column (250 × 32 mm) and the fraction collector Foxy R1 was used. The eluents were the same as mentioned above. For method C, linear gradients from 5–95 % B over 20 min and for method D over 25 min were used. The methods were carried out with a flow rate of 20 mL/min at room temperature and the chromatograms were acquired via UV absorption detection at 220 and 254 nm.

#### 3.1.3. Nuclear Magnetic Resonance Spectroscopy (NMR)

For the characterization of the compounds proton (^1^H), carbon (^13^C), and fluorine (^19^F), NMR spectra were acquired on a Bruker (Billerica, MA, USA) Avance III HD 400, a Varian/Agilent (Santa Clara, CA, USA) Mercury Plus 400, or a Varian/Agilent Mercury Plus 300 at room temperature. The used frequencies were 300 or 400 MHz for the ^1^H spectra, 75, 76, or 101 MHz for the ^13^C spectra and 377 MHz for the ^19^F spectra. The chemical shifts δ were given in parts per million (ppm) and normalized on the residual solvent signal of the deuterated solvents (CDCl_3_: ^1^H-NMR: 7.26 ppm, ^13^C-NMR: 77.16 ppm; DMSO-*d_6_*: ^1^H-NMR: 2.50 ppm, ^13^C-NMR: 39.52 ppm). The coupling constants *J* were given in Hertz (Hz), and the multiplicities of the signals were described as singlet (s), doublet (d), triplet (t), quartet (q), quintet (p), multiplet (m), and their corresponding combinations. They were given as they were measured and thus might disagree with the expected values. 

#### 3.1.4. Mass Spectrometry (MS)

For the characterization of the compounds, high resolution electrospray ionisation mass spectra (HR-ESI-MS) were recorded with a Bruker Daltonics (Bremen, Germany) micrOTOF coupled to a LC Packings Ultimate HPLC system and controlled by micrOTOFControl3.4 and HyStar 3.2-LC/MS. 

#### 3.1.5. TNBS-Test

One drop of 2,4,6-trinitrobenzenesulfonic acid in DMF (1%) and DIPEA in DMF (10%) were added to some resin beads and incubated for 3 min. A red colouring of the resin beads confirmed the presence of free amino groups.

#### 3.1.6. Determination of the Loading

The loading was determined on a Shimadzu (Kyōto, Japan) double beam photometer UV-160A. To a defined amount of the resin (exact weight 5 mg), a solution of 20% piperidine in DMF (500 µL) was added and incubated for 5 min at room temperature. The solution was transferred to another tube and the remaining resin was treated with the mentioned deprotection solution (500 µL) again for 5 min. The solutions were combined and the absorbance was measured at 300 nm at room temperature in a quartz cuvette (volume: 3500 µL, path length: 10 mm, 100-QS, Hellma Analytics (Müllheim, Germany)). By using the Lambert-Beer law A = ε*c*d (A = absorbance, ε = molar extinction coefficient with ε_300 nm_ (dibenzofulvene) = 7800 L/(mol*cm), c = concentration in mol/L, d = optical path length in cm), the concentration was calculated, and by including the mass of the weighed resin, the loading in mmol/g was determined. 

#### 3.1.7. Determination of the LogD_7.4HPLC_

The log*D*_7.4HPLC_ value was determined as previously reported by us [41] utilizing a modified HPLC method originally described by Donovan and Pescatore [46]. Hydrocortisone (t_R_ = 10.70 min, log*D*_7.4_ = 1,46) and triphenylene (t_R_ = 29.50 min, log*D*_7.4_ = 5.49) served as references to calculate log*D*_7.4HPLC_ as given in formula 4 of reference [46] and toluene (t_R_ = 16.51 min, measured log*D*_7.4HPLC_ = 2.71, literature log*D*_7.4_ = 2.72) as internal reference.

### 3.2. General Procedures

#### General Procedure A: Coupling of the Cap Group

The given amounts refer to a synthesis scale of 0.1 mmol of the linker-modified resins **PR1-7**. The linker-modified resin **PR1-7** (1.00 eq., defined loading) was swollen in DMF (1.5 mL) for 30 min. Then, the Fmoc protecting group was cleaved by treatment with 20% piperidine in DMF (1.5 mL) for 5 min. This step was repeated once. Afterwards, the resin was washed with DMF (5 × 2 mL) and DCM (5 × 2 mL). To confirm that the Fmoc deprotection was successful, a TNBS-test was carried out with some resin beads, and afterwards the resin was washed with DMF (5 × 2 mL) again. Next, a solution of the respective acid Cap-COOH **A–C** (2.00 eq.), HATU (2.00 eq.), and DIPEA (3.00 eq.) in DMF (1 mL/mmol acid) was agitated for 5 min, added to the resin, and incubated for 18 h at room temperature. Subsequently, the resin was washed with DMF (10 × 2 mL) and DCM (10 × 2 mL). The completion of the reaction was monitored by the TNBS-test and the resin was washed with DCM (10 × 2 mL) again and dried in vacuo. For the cleavage of the coupling product, the resin was treated with 5% TFA in DCM (1 mL/40 mg resin) for 1 h at room temperature. The resulting solution was concentrated under reduced pressure followed by the purification of the crude products **A1–A7**, **B1–B7**, and **C2–C4** via (semi)-preparative HPLC. The product fractions were lyophilized, and the products **A1–A7**, **B2–B7**, and **C2–C4** were isolated in purities of 95% or higher, while **B1** was obtained in a purity of 89%.

### 3.3. Syntheses

#### 3.3.1. Building Block Synthesis

Methyl 4-(aminomethyl)-3-fluorobenzoate (**8**): NaN_3_ (0.38 g, 5.9 mmol, 1.30 eq.) was dissolved in distilled water (4 mL) under argon atmosphere, and acetone (6 mL) was added. Subsequently, a solution of methyl 4-(bromomethyl)-3-fluorobenzoate (1.11 g, 4.5 mmol, 1.00 eq.) in acetone (6 mL) was added to the NaN_3_ solution and the mixture was stirred for 2 h at room temperature, followed by 2 h at 75 °C. Anhydrous MgSO_4_ (3.99 g, 33.1 mmol, 7.36 eq.) was added to bind the water in the reaction mixture. After further stirring for 1 h at a temperature of 75 °C, once again NaN_3_ (0.38 g, 5.9 mmol, 1.30 eq.) was added and the mixture was stirred overnight at 75 °C. The complete conversion into methyl 4-(azidomethyl)-3-fluorobenzoate was confirmed by HPLC. After the solution cooled down to room temperature, distilled water (5 mL) was added. The acetone phase was removed under reduced pressure and the remaining aqueous layer was extracted with EtOAc (3 × 20 mL). The organic phase was washed with brine (30 mL), dried over anhydrous MgSO_4_, filtrated, and the solvent was removed under reduced pressure. Methyl 4-(azidomethyl)-3-fluorobenzoate was isolated as a colorless oil and directly dissolved in MeOH (25 mL) under argon atmosphere. Pd/C (0.09 g, 0.45 mmol, 0.10 eq.) was added and the mixture was stirred under hydrogen atmosphere for 2 h at room temperature. After the reaction mixture was filtrated over Celite 545, the solvent was removed under reduced pressure. The crude product was purified by flash column chromatography (1–10% MeOH/DCM). The desired product **8** (0.34 g, 1.9 mmol, 42%) was isolated as a yellow solid. R_f_ = 0.53 (DCM/MeOH 90:10). ^1^H-NMR (400 MHz, DMSO-*d_6_*): δ = 7.78 (dd, *J* = 7.9, 1.6 Hz, 1H), 7.67 (t, *J* = 7.6 Hz, 1H), 7.63 (dd, *J* = 10.5, 1.6 Hz, 1H), 3.85 (s, 3H), 3.81 (s, 2H), 3.32 (s, 2H) ppm. ^13^C-NMR (101 MHz, DMSO-*d_6_*): δ = 165.2 (d, ^4^*J*_C-F_ = 3.0 Hz), 160.0 (d, ^1^*J*_C-F_ = 245.4 Hz), 133.1 (d, ^2^*J*_C-F_ = 15.0 Hz), 130.4 (d, ^3^*J*_C-F_ = 4.9 Hz), 130.0 (d, ^3^*J*_C-F_ = 7.6 Hz), 125.1 (d, ^4^*J*_C-F_ = 3.3 Hz), 115.4 (d, ^2^*J*_C-F_ = 23.9 Hz), 52.3, 45.1 (d, ^3^*J*_C-F_ = 3.1 Hz) ppm. HRMS-ESI (*m*/*z*): [M + H]^+^ calc. for C_9_H_10_FNO_2_: 184.0768; found: 184.0780.

4-(((((9*H*-Fluoren-9-yl)methoxy)carbonyl)amino)methyl)-3-fluorobenzoic acid (**9**): Methyl 4-(aminomethyl)-3-fluorobenzoate **8** (0.33 g, 1.8 mmol, 1.00 eq.) was dissolved in THF/MeOH (5:1, 48 mL). NaOH (50 mg/mL, 5.76 mL, 7.2 mmol, 4.00 eq.) was added and the reaction solution was stirred for 2 h at room temperature. After completion of the ester hydrolysis, which was monitored by HPLC, 1M HCl (7.20 mL, 7.2 mmol, 4.00 eq.) was added to neutralize the solution. The organic solvent was removed under reduced pressure to obtain the intermediate 4-(aminomethyl)-3-fluorobenzoic acid in the aqueous layer, which was diluted with 5% aqueous Na_2_CO_3_ solution/1,4-dioxane (3:2, 34 mL, 19 mL/mmol). After the solution was cooled down to 0 °C, Fmoc-Cl (0.70 g, 2.7 mmol, 1.50 eq.) was added slowly over 20 min and subsequently the mixture, allowed to warm to room temperature, and stirred for 18 h. Distilled water (30 mL) was added and the mixture was washed with Et_2_O (3 × 50 mL). The aqueous layer was acidified with 6M HCl to pH 4. Afterwards, the formed precipitate was collected by filtration, washed with distilled water (pH 4, 100 mL), and dried in vacuo to yield the desired product **9** (0.45 g, 1.2 mmol, 64%) as a white solid. R_f_ = 0.17 (DCM/MeOH 95:5). ^1^H-NMR (400 MHz, DMSO-*d_6_*): δ = 7.93 (d, *J* = 6.0 Hz, 1H), 7.89 (d, *J* = 7.7 Hz, 2H), 7.74 (dd, *J* = 7.9, 1.6 Hz, 1H), 7.70 (d, *J* = 7.5 Hz, 2H), 7.62 (dd, *J* = 10.5, 1.6 Hz, 1H), 7.42 (t, *J* = 7.4 Hz, 2H), 7.38 − 7.28 (m, 2H), 4.37 (d, *J* = 6.7 Hz, 2H), 4.28 (d, *J* = 6.0 Hz, 2H), 4.23 (t, *J* = 6.7 Hz, 1H) ppm, two signals could not be detected due to solvent exchange. ^13^C-NMR (75 MHz, DMSO-*d_6_*): δ = 166.1 (d, ^4^*J*_C-F_ = 1.7 Hz), 159.5 (d, ^1^*J*_C-F_ = 246.1 Hz), 156.3, 143.8 (2C), 140.8 (2C), 131.7 (d, ^3^*J*_C-F_ = 7.3 Hz), 131.5 (d, ^2^*J*_C-F_ = 14.8 Hz), 129.3 (d, ^3^*J*_C-F_ = 4.1 Hz), 127.6 (2C), 127.0 (2C), 125.3 (d, ^4^*J*_C-F_ = 2.9 Hz), 125.1 (2C), 120.1 (2C), 115.6 (d, ^2^*J*_C-F_ = 23.2 Hz), 65.4, 46.8, 37.6 ppm. HRMS-ESI (*m*/*z*): [M + Na]^+^ calc. for C_23_H_18_FNO_4_: 414.1112; found: 414.1124.

#### 3.3.2. Library Synthesis

4-(2-(1-(4-Chlorobenzoyl)-5-methoxy-2-methyl-1*H*-indol-3-yl)acetamido)-*N*-hydroxybutanamide (**A1**): According to General Procedure A, the Fmoc-propyl linker-preloaded resin **PR1** (0.14 g, 0.1 mmol, 1.00 eq., loading: 0.71 mmol/g) was Fmoc-deprotected and subsequently treated with a solution of indometacin (0.07 g, 0.2 mmol, 2.00 eq.), HATU (0.08 g, 0.2 mmol, 2.00 eq.) and DIPEA (0.05 mL, 0.3 mmol, 3.00 eq.) in DMF (0.4 mL). The cleavage from the resin was performed with 4 mL of the cleavage cocktail. After purification via preparative HPLC (method B), the desired product **A1** (33 mg, 0.07 mmol, 71%) was isolated as a white solid in a purity of 99%. t_R_ = 16.99 min. ^1^H-NMR (400 MHz, DMSO-*d_6_*): δ = 10.34 (s, 1H), 8.04 (t, *J* = 5.6 Hz, 1H), 7.71 − 7.63 (m, 4H), 7.10 (d, *J* = 2.5 Hz, 1H), 6.94 (d, *J* = 9.0 Hz, 1H), 6.70 (dd, *J* = 9.0, 2.6 Hz, 1H), 3.76 (s, 3H), 3.49 (s, 2H), 3.04 (q, *J* = 6.7 Hz, 2H), 2.22 (s, 3H), 1.94 (t, *J* = 7.5 Hz, 2H), 1.62 (p, *J* = 7.6 Hz, 2H) ppm. ^13^C-NMR (101 MHz, DMSO-*d_6_*): δ = 169.3, 168.8, 167.8, 155.5, 137.5, 135.1, 134.3, 131.1 (2C), 130.9, 130.3, 129.0 (2C), 114.5, 114.4, 111.3, 101.8, 55.4, 38.4, 31.2, 29.9, 25.4, 13.4 ppm. HRMS-ESI (*m*/*z*): [M + H]^+^ calc. for C_23_H_24_ClN_3_O_5_: 458.1477; found: 458.1486.

6-(2-(1-(4-Chlorobenzoyl)-5-methoxy-2-methyl-1*H*-indol-3-yl)acetamido)-*N*-hydroxyhexanamide (**A2**): According to General Procedure A, the Fmoc-pentyl linker-preloaded resin **PR2** (0.29 g, 0.2 mmol, 1.00 eq., loading: 0.70 mmol/g) was Fmoc-deprotected and subsequently treated with a solution of indometacin (0.14 g, 0.4 mmol, 2.00 eq.), HATU (0.15 g, 0.4 mmol, 2.00 eq.), and DIPEA (0.10 mL, 0.6 mmol, 3.00 eq.) in DMF (0.6 mL). The cleavage from the resin was performed with 8 mL of the cleavage cocktail. After purification via preparative HPLC (method B), the desired product **A2** (48 mg, 0.10 mmol, 49%) was isolated as a white solid in a purity of 99%. t_R_ = 17.47 min. ^1^H-NMR (400 MHz, DMSO-*d_6_*): δ = 10.31 (s, 1H), 8.01 (t, *J* = 5.6 Hz, 1H), 7.71 − 7.62 (m, 4H), 7.11 (d, *J* = 2.5 Hz, 1H), 6.93 (d, *J* = 9.0 Hz, 1H), 6.70 (dd, *J* = 9.0, 2.6 Hz, 1H), 3.76 (s, 3H), 3.48 (s, 2H), 3.03 (q, *J* = 6.6 Hz, 2H), 2.22 (s, 3H), 1.91 (t, *J* = 7.4 Hz, 2H), 1.42 (dp, *J* = 29.1, 7.4 Hz, 4H), 1.22 (q, *J* = 8.6 Hz, 2H) ppm. ^13^C-NMR (101 MHz, DMSO-*d_6_*): δ = 169.2, 169.0, 167.8, 155.5, 137.5, 135.1, 134.3, 131.1 (2C), 130.9, 130.3, 129.0 (2C), 114.5, 114.5, 111.2, 101.9, 55.4, 38.6, 32.2, 31.2, 28.9, 26.0, 24.9, 13.4 ppm. HRMS-ESI (*m*/*z*): [M + H]^+^ calc. for C_25_H_28_ClN_3_O_5_: 486.1790; found: 486.1814.

7-(2-(1-(4-Chlorobenzoyl)-5-methoxy-2-methyl-1*H*-indol-3-yl)acetamido)-*N*-hydroxyheptanamide (**A3**): According to General Procedure A, the Fmoc-hexyl linker-preloaded resin **PR3** (0.26 g, 0.2 mmol, 1.00 eq., loading: 0.77 mmol/g) was Fmoc-deprotected and subsequently treated with a solution of indometacin (0.14 g, 0.4 mmol, 2.00 eq.), HATU (0.15 g, 0.4 mmol, 2.00 eq.), and DIPEA (0.10 mL, 0.6 mmol, 3.00 eq.) in DMF (0.4 mL). The cleavage from the resin was performed with 7.5 mL of the cleavage cocktail. After purification via preparative HPLC (method B), the desired product **A3** (68 mg, 0.14 mmol, 68%) was isolated as a white solid in a purity of 98%. t_R_ = 17.81 min. ^1^H-NMR (300 MHz, DMSO-*d_6_*): δ = 10.31 (s, 1H), 8.00 (t, *J* = 5.6 Hz, 1H), 7.71 – 7.62 (m, 4H), 7.11 (d, *J* = 2.4 Hz, 1H), 6.94 (d, *J* = 9.0 Hz, 1H), 6.70 (dd, *J* = 9.0, 2.6 Hz, 1H), 3.76 (s, 3H), 3.48 (s, 2H), 3.04 (q, *J* = 6.6 Hz, 2H), 2.22 (s, 3H), 1.91 (t, *J* = 7.3 Hz, 2H), 1.41 (dt, *J* = 15.9, 6.9 Hz, 4H), 1.22 (d, *J* = 4.3 Hz, 4H) ppm. ^13^C-NMR (76 MHz, DMSO-*d_6_*): δ = 169.2, 169.1, 167.9, 155.5, 137.5, 135.1, 134.3, 131.1 (2C), 130.9, 130.3, 129.0 (2C), 114.5, 114.5, 111.2, 101.9, 55.4, 39.0 *, 32.2, 31.2, 29.1, 28.3, 26.1, 25.1, 13.4 ppm, * overlapping with DMSO-signal. HRMS-ESI (*m*/*z*): [M + H]^+^ calc. for C_26_H_30_ClN_3_O_5_: 500.1947; found: 500.1957.

4-((2-(1-(4-Chlorobenzoyl)-5-methoxy-2-methyl-1*H*-indol-3-yl)acetamido)methyl)-*N*-hydroxybenzamide (**A4**): According to General Procedure A, the Fmoc-benzyl linker-preloaded resin **PR4** (0.29 g, 0.2 mmol, 1.00 eq., loading: 0.69 mmol/g) was Fmoc-deprotected and subsequently treated with a solution of indometacin (0.21 g, 0.6 mmol, 3.00 eq.), HATU (0.23 g, 0.6 mmol, 3.00 eq.), and DIPEA (0.17 mL, 1.0 mmol, 5.00 eq.) in DMF (0.6 mL). The cleavage from the resin was performed with 8 mL of the cleavage cocktail. After purification via preparative HPLC (method A), the desired product **A4** (44 mg, 0.09 mmol, 44%) was isolated as a white solid in a purity of 97%. t_R_ = 17.80 min. ^1^H-NMR (400 MHz, DMSO-*d_6_*): δ = 11.17 (s, 1H), 8.56 (t, *J* = 6.0 Hz, 1H), 7.71 − 7.62 (m, 6H), 7.29 (d, *J* = 8.1 Hz, 2H), 7.13 (d, *J* = 2.5 Hz, 1H), 6.96 (d, *J* = 9.0 Hz, 1H), 6.72 (dd, *J* = 9.0, 2.5 Hz, 1H), 4.32 (d, *J* = 5.9 Hz, 2H), 3.74 (s, 3H), 3.60 (s, 2H), 2.24 (s, 3H) ppm. ^13^C-NMR (101 MHz, DMSO-*d_6_*): δ = 169.6, 167.9, 164.0, 155.6, 142.8, 137.6, 135.2, 134.2, 131.2, 131.1 (2C), 130.8, 130.3, 129.0 (2C), 126.9 (2C), 126.8 (2C), 114.6, 114.2, 111.4, 101.8, 55.4, 42.0, 31.1, 13.4 ppm. HRMS-ESI (*m*/*z*): [M + H]^+^ calc. for C_27_H_24_ClN_3_O_5_: 506.1477; found: 506.1475.

4-((2-(1-(4-Chlorobenzoyl)-5-methoxy-2-methyl-1*H*-indol-3-yl)acetamido)methyl)-3-fluoro-*N*-hydroxybenzamide (**A5**): According to General Procedure A, the Fmoc-fluorobenzyl linker-preloaded resin **PR5** (0.21 g, 0.1 mmol, 1.00 eq., loading: 0.48 mmol/g) was Fmoc-deprotected and subsequently treated with a solution of indometacin (0.07 g, 0.2 mmol, 2.00 eq.), HATU (0.08 g, 0.2 mmol, 2.00 eq.), and DIPEA (0.05 mL, 0.3 mmol, 3.00 eq.) in DMF (0.4 mL). The cleavage from the resin was performed with 5.5 mL of the cleavage cocktail. After purification via preparative HPLC (method D), the desired product **A5** (34 mg, 0.06 mmol, 65%) was isolated as a white solid in a purity of 96%. t_R_ = 18.17 min. ^1^H-NMR (400 MHz, DMSO-*d_6_*): δ = 11.28 (s, 1H), 8.57 (t, *J* = 5.9 Hz, 1H), 7.70 − 7.63 (m, 4H), 7.52 (t, *J* = 1.9 Hz, 1H), 7.50 (dd, *J* = 4.9, 1.5 Hz, 1H), 7.34 (t, *J* = 7.9 Hz, 1H), 7.12 (d, *J* = 2.6 Hz, 1H), 6.95 (d, *J* = 9.0 Hz, 1H), 6.71 (dd, *J* = 9.0, 2.5 Hz, 1H), 4.34 (d, *J* = 5.7 Hz, 2H), 3.74 (s, 3H), 3.60 (s, 2H), 2.23 (s, 3H) ppm. ^13^C-NMR (101 MHz, DMSO-*d_6_*): δ = 169.7, 167.9, 162.6, 159.6 (d, ^1^*J*_C-F_ = 245.3 Hz), 155.6, 137.6, 135.3, 134.2, 133.5 (d, ^3^*J*_C-F_ = 7.0 Hz), 131.1 (2C), 130.8, 130.3, 129.4 (d, ^3^*J*_C-F_ = 4.1 Hz), 129.3 (d, ^2^*J*_C-F_ = 14.2 Hz), 129.0 (2C), 122.6 (d, ^4^*J*_C-F_ = 2.4 Hz), 114.6, 114.1, 113.5 (d, ^2^*J*_C-F_ = 23.3 Hz), 111.4, 101.7, 55.4, 36.1 (d, ^3^*J*_C-F_ = 3.5 Hz), 31.0, 13.4 ppm. HRMS-ESI (*m*/*z*): [M + H]^+^ calc. for C_27_H_23_ClFN_3_O_5_: 524.1383; found: 524.1391.

4-((5-(2-(1-(4-Chlorobenzoyl)-5-methoxy-2-methyl-1*H*-indol-3-yl)acetamido)-2-methyl-1*H*-benzo[*d*]imidazol-1-yl)methyl)-3-fluoro-*N*-hydroxybenzamide (TFA salt) (**A6**): According to General Procedure A, the Fmoc-benzimidazole-fluorobenzyl linker-preloaded resin **PR6** (0.15 g, 0.1 mmol, 1.00 eq., loading: 0.65 mmol/g) was Fmoc-deprotected and subsequently treated with a solution of indometacin (0.07 g, 0.2 mmol, 2.00 eq.), HATU (0.08 g, 0.2 mmol, 2.00 eq.), and DIPEA (0.05 mL, 0.3 mmol, 3.00 eq.) in DMF (0.35 mL). The cleavage from the resin was performed with 5 mL of the cleavage cocktail. After purification via preparative HPLC (method C), the desired product **A6** (60 mg, 0.08 mmol, 78%) was isolated as a light-yellow solid (TFA salt) in a purity of 97%. t_R_ = 16.30 min. ^1^H-NMR (400 MHz, DMSO-*d_6_*): δ = 11.36 (s, 1H), 10.61 (s, 1H), 8.24 (d, *J* = 1.9 Hz, 1H), 7.70 (s, 1H), 7.68 (s, 2H), 7.65 (d, *J* = 8.6 Hz, 2H), 7.63 − 7.56 (m, 2H), 7.54 (dd, *J* = 9.0, 1.9 Hz, 1H), 7.35 (t, *J* = 7.8 Hz, 1H), 7.19 (d, *J* = 2.5 Hz, 1H), 6.93 (d, *J* = 8.9 Hz, 1H), 6.72 (dd, *J* = 9.0, 2.6 Hz, 1H), 5.76 (s, 2H), 3.81 (s, 2H), 3.74 (s, 3H), 2.79 (s, 3H), 2.30 (s, 3H) ppm. ^13^C-NMR (101 MHz, DMSO-*d_6_*): δ = 168.9, 167.9, 162.1, 159.8 (d, ^1^*J*_C-F_ = 247.3 Hz), 158.2 (d, ^2^*J*_C-F_ = 32.8 Hz, TFA), 155.6, 152.1, 137.6, 136.9, 135.5, 135.0 (d, ^3^*J*_C-F_ = 7.1 Hz), 134.2, 131.2 (2C), 130.9, 130.3, 129.7 (d, ^3^*J*_C-F_ = 3.4 Hz), 129.1 (2C), 128.2, 124.5 (d, ^2^*J*_C-F_ = 14.0 Hz), 123.2, 117.5, 116.8 (d, ^1^*J*_C-F_ = 297.3 Hz, TFA), 114.6, 114.2 (d, ^2^*J*_C-F_ = 23.4 Hz), 113.9, 112.6, 111.2, 104.4, 102.0, 55.4, 42.3, 32.0, 13.4, 12.0 ppm. ^19^F-NMR (377 MHz, DMSO-*d_6_*): δ = −73.92, −115.75 – −115.87 (m) ppm. HRMS-ESI (*m*/*z*): [M + H]^+^ calc. for C_35_H_29_ClFN_5_O_5_: 654.1914; found: 654.1932.

(*E*)-3-(4-((2-(1-(4-Chlorobenzoyl)-5-methoxy-2-methyl-1*H*-indol-3-yl)acetamido)methyl)phenyl)-*N*-hydroxyacrylamide (**A7**): According to General Procedure A, the Fmoc-phenylvinyl linker-preloaded resin **PR7** (0.14 g, 0.1 mmol, 1.00 eq., loading: 0.69 mmol/g) was Fmoc-deprotected and subsequently treated with a solution of indometacin (0.07 g, 0.2 mmol, 2.00 eq.), HATU (0.08 g, 0.2 mmol, 2.00 eq.), and DIPEA (0.05 mL, 0.3 mmol, 3.00 eq.) in DMF (0.4 mL). The cleavage from the resin was performed with 5 mL of the cleavage cocktail. After purification via preparative HPLC (method D), the desired product **A7** (38 mg, 0.07 mmol, 71%) was isolated as a white solid in a purity of 95%. t_R_ = 18.17 min. ^1^H-NMR (400 MHz, DMSO-*d_6_*): δ = 10.73 (s, 1H), 8.54 (t, *J* = 5.9 Hz, 1H), 7.70–7.62 (m, 4H), 7.52 − 7.37 (m, 3H), 7.26 (d, *J* = 8.0 Hz, 2H), 7.12 (d, *J* = 2.5 Hz, 1H), 6.97 (d, *J* = 9.0 Hz, 1H), 6.72 (dd, *J* = 9.0, 2.6 Hz, 1H), 6.43 (d, *J* = 15.8 Hz, 1H), 4.29 (d, *J* = 5.9 Hz, 2H), 3.73 (s, 3H), 3.59 (s, 2H), 2.23 (s, 3H) ppm. ^13^C-NMR (101 MHz, DMSO-*d_6_*): δ = 169.5, 167.9, 162.7, 155.6, 141.1, 138.0, 137.5, 135.2, 134.3, 133.4, 131.1 (2C), 130.8, 130.3, 129.0 (2C), 127.7 (2C), 127.4 (2C), 118.6, 114.6, 114.2, 111.4, 101.8, 55.4, 42.1, 31.2, 13.4 ppm. HRMS-ESI (*m*/*z*): [M + H]^+^ calc. for C_29_H_26_ClN_3_O_5_: 532.1634; found: 532.1638.

*N*-(4-(Hydroxyamino)-4-oxobutyl)-1-(4-sulfamoylphenyl)-5-(*p*-tolyl)-1*H*-pyrazole-3-carboxamide (**B1**): According to General Procedure A, the Fmoc-propyl linker-preloaded resin **PR1** (0.14 g, 0.1 mmol, 1.00 eq., loading: 0.71 mmol/g) was Fmoc-deprotected and subsequently treated with a solution of 1-(4-sulfamoylphenyl)-5-(*p*-tolyl)-1*H*-pyrazole-3-carboxylic acid (0.07 g, 0.2 mmol, 2.00 eq.), HATU (0.08 g, 0.2 mmol, 2.00 eq.), and DIPEA (0.05 mL, 0.3 mmol, 3.00 eq.) in DMF (0.4 mL). The cleavage from the resin was performed with 4 mL of the cleavage cocktail. After purification via preparative HPLC (method B), the desired product **B1** (29 mg, 0.06 mmol, 64%) was isolated as a white solid in a purity of 89% and was used without further purification. t_R_ = 14.93 min. ^1^H-NMR (400 MHz, DMSO-*d_6_*): δ = 10.37 (s, 1H), 8.40 (t, *J* = 5.9 Hz, 1H), 7.86 (d, *J* = 8.7 Hz, 2H), 7.61 − 7.36 (m, 4H), 7.24 − 7.13 (m, 4H), 6.97 (s, 1H), 3.25 (q, *J* = 6.7 Hz, 2H), 2.31 (s, 3H), 2.05 − 1.95 (m, 2H), 1.74 (p, *J* = 7.3 Hz, 2H) ppm. ^13^C-NMR (75 MHz, DMSO-*d_6_*): δ = 168.8, 160.9, 147.8, 144.5, 143.4, 141.7, 138.6, 129.4 (2C), 128.6 (2C), 126.6 (2C), 126.2, 125.8 (2C), 108.0, 38.3, 30.0, 25.5, 20.8 ppm. HRMS-ESI (*m*/*z*): [M + H]^+^ calc. for C_21_H_23_N_5_O_5_S: 458.1493; found: 458.1502.

*N*-(6-(Hydroxyamino)-6-oxohexyl)-1-(4-sulfamoylphenyl)-5-(*p*-tolyl)-1*H*-pyrazole-3-carboxamide (**B2**): According to General Procedure A, the Fmoc-pentyl linker-preloaded resin **PR2** (0.14 g, 0.1 mmol, 1.00 eq., loading: 0.70 mmol/g) was Fmoc-deprotected and subsequently treated with a solution of 1-(4-sulfamoylphenyl)-5-(*p*-tolyl)-1*H*-pyrazole-3-carboxylic acid (0.07 g, 0.2 mmol, 2.00 eq.), HATU (0.08 g, 0.2 mmol, 2.00 eq.), and DIPEA (0.05 mL, 0.3 mmol, 3.00 eq.) in DMF (0.4 mL). The cleavage from the resin was performed with 4 mL of the cleavage cocktail. After purification via preparative HPLC (method B), the desired product **B2** (34 mg, 0.07 mmol, 70%) was isolated as a white solid in a purity > 99%. t_R_ = 15.39 min. ^1^H-NMR (400 MHz, DMSO-*d_6_*): δ = 10.32 (s, 1H), 8.31 (t, *J* = 6.0 Hz, 1H), 7.86 (d, *J* = 8.6 Hz, 2H), 7.55 − 7.44 (m, 4H), 7.19 (q, *J* = 8.3 Hz, 4H), 6.96 (s, 1H), 3.24 (q, *J* = 6.7 Hz, 2H), 2.31 (s, 3H), 1.95 (t, *J* = 7.4 Hz, 2H), 1.51 (p, *J* = 7.4 Hz, 4H), 1.27 (q, *J* = 8.7 Hz, 2H) ppm. ^13^C-NMR (101 MHz, DMSO-*d_6_*): δ = 169.1, 160.8, 147.9, 144.5, 143.4, 141.7, 138.6, 129.4 (2C), 128.6 (2C), 126.6 (2C), 126.2, 125.7 (2C), 108.0, 38.4, 32.2, 29.0, 26.0, 24.9, 20.8 ppm. HRMS-ESI (*m*/*z*): [M + H]^+^ calc. for C_23_H_27_N_5_O_5_S: 486.1806; found: 486.1805.

*N*-(7-(Hydroxyamino)-7-oxoheptyl)-1-(4-sulfamoylphenyl)-5-(*p*-tolyl)-1*H*-pyrazole-3-carboxamide (**B3**): According to General Procedure A, the Fmoc-hexyl linker-preloaded resin **PR3** (0.13 g, 0.1 mmol, 1.00 eq., loading: 0.77 mmol/g) was Fmoc-deprotected and subsequently treated with a solution of 1-(4-sulfamoylphenyl)-5-(*p*-tolyl)-1*H*-pyrazole-3-carboxylic acid (0.07 g, 0.2 mmol, 2.00 eq.), HATU (0.08 g, 0.2 mmol, 2.00 eq.), and DIPEA (0.05 mL, 0.3 mmol, 3.00 eq.) in DMF (0.4 mL). The cleavage from the resin was performed with 4 mL of the cleavage cocktail. After purification via preparative HPLC (method D), the desired product **B3** (20 mg, 0.04 mmol, 41%) was isolated as a white solid in a purity > 99%. t_R_ = 15.77 min, ^1^H-NMR (400 MHz, DMSO-*d_6_*): δ = 10.33 (s, 1H), 8.31 (t, *J* = 6.0 Hz, 1H), 7.86 (d, *J* = 8.6 Hz, 2H), 7.56 − 7.44 (m, 4H), 7.19 (q, *J* = 8.3 Hz, 4H), 6.96 (s, 1H), 3.24 (q, *J* = 6.7 Hz, 2H), 2.31 (s, 3H), 1.94 (t, *J* = 7.3 Hz, 2H), 1.50 (q, *J* = 7.2 Hz, 4H), 1.31 − 1.23 (m, 4H) ppm. ^13^C-NMR (101 MHz, DMSO-*d_6_*): δ = 169.1, 160.8, 147.9, 144.5, 143.4, 141.7, 138.6, 129.4 (2C), 128.6 (2C), 126.6 (2C), 126.2, 125.7 (2C), 108.0, 38.5, 32.2, 29.1, 28.4, 26.2, 25.1, 20.8 ppm. HRMS-ESI (*m*/*z*): [M + H]^+^ calc. for C_24_H_29_N_5_O_5_S: 500.1962; found: 500.1960.

*N*-(4-(Hydroxycarbamoyl)benzyl)-1-(4-sulfamoylphenyl)-5-(*p*-tolyl)-1*H*-pyrazole-3-carboxamide (**B4**): According to General Procedure A, the Fmoc-benzyl linker-preloaded resin **PR4** (0.14 g, 0.1 mmol, 1.00 eq., loading: 0.69 mmol/g) was Fmoc-deprotected and subsequently treated with a solution of 1-(4-sulfamoylphenyl)-5-(*p*-tolyl)-1*H*-pyrazole-3-carboxylic acid (0.07 g, 0.2 mmol, 2.00 eq.), HATU (0.08 g, 0.2 mmol, 2.00 eq.), and DIPEA (0.05 mL, 0.3 mmol, 3.00 eq.) in DMF (0.4 mL). The cleavage from the resin was performed with 4.5 mL of the cleavage cocktail. After purification via preparative HPLC (method B), the desired product **B4** (29 mg, 0.06 mmol, 57%) was isolated as a white solid in a purity > 99%. t_R_ = 15.82 min. ^1^H-NMR (400 MHz, DMSO-*d_6_*): δ = 11.16 (s, 1H), 9.00 (t, *J* = 6.3 Hz, 1H), 7.86 (d, *J* = 8.6 Hz, 2H), 7.71 (d, *J* = 8.3 Hz, 2H), 7.54 (d, *J* = 8.6 Hz, 2H), 7.48 (s, 2H), 7.38 (d, *J* = 8.4 Hz, 2H), 7.23 − 7.17 (m, 4H), 7.01 (s, 1H), 4.50 (d, *J* = 6.3 Hz, 2H), 2.31 (s, 3H) ppm. ^13^C-NMR (101 MHz, DMSO-*d_6_*): δ = 164.1, 161.0, 147.5, 144.6, 143.5, 142.9, 141.6, 138.6, 131.3, 129.4 (2C), 128.6 (2C), 127.1 (2C), 126.9 (2C), 126.6 (2C), 126.2, 125.8 (2C), 108.1, 41.9, 20.8 ppm. HRMS-ESI (*m*/*z*): [M + H]^+^ calc. for C_25_H_23_N_5_O_5_S: 506.1493; found: 506.1507.

*N*-(2-Fluoro-4-(hydroxycarbamoyl)benzyl)-1-(4-sulfamoylphenyl)-5-(*p*-tolyl)-1*H*-pyrazole-3-carboxamide (**B5**): According to General Procedure A, the Fmoc-fluorobenzyl linker-preloaded resin **PR5** (0.21 g, 0.1 mmol, 1.00 eq., loading: 0.48 mmol/g) was Fmoc-deprotected and subsequently treated with a solution of 1-(4-sulfamoylphenyl)-5-(*p*-tolyl)-1*H*-pyrazole-3-carboxylic acid (0.07 g, 0.2 mmol, 2.00 eq.), HATU (0.08 g, 0.2 mmol, 2.00 eq.), and DIPEA (0.05 mL, 0.3 mmol, 3.00 eq.) in DMF (0.4 mL). The cleavage from the resin was performed with 5.5 mL of the cleavage cocktail. After purification via preparative HPLC (method D), the desired product **B5** (33 mg, 0.06 mmol, 63%) was isolated as a white solid in a purity > 99%. t_R_ = 16.16 min. ^1^H-NMR (400 MHz, DMSO-*d_6_*): δ = 11.28 (s, 1H), 8.98 (t, *J* = 6.1 Hz, 1H), 7.86 (d, *J* = 8.6 Hz, 2H), 7.58 (dd, *J* = 8.0, 1.7 Hz, 1H), 7.56 − 7.50 (m, 3H), 7.48 (s, 2H), 7.43 (t, *J* = 7.8 Hz, 1H), 7.23 − 7.16 (m, 4H), 7.02 (s, 1H), 4.54 (d, *J* = 6.0 Hz, 2H), 2.31 (s, 3H) ppm. ^13^C-NMR (101 MHz, DMSO-*d_6_*): δ = 162.7, 161.1, 159.5 (d, ^1^*J*_C-F_ = 245.2 Hz), 147.4, 144.7, 143.5, 141.6, 138.7, 133.4 (d, ^3^*J*_C-F_ = 6.9 Hz), 129.4 (2C), 129.3, 129.3, 128.6 (2C), 126.7 (2C), 126.1, 125.8 (2C), 122.8, 113.5 (d, ^2^*J*_C-F_ = 23.6 Hz), 108.1, 35.9 (d, ^3^*J*_C-F_ = 3.7 Hz), 20.8 ppm. HRMS-ESI (*m*/*z*): [M + H]^+^ calc. for C_25_H_22_FN_5_O_5_S: 524.1398; found: 524.1381.

*N*-(1-(2-Fluoro-4-(hydroxycarbamoyl)benzyl)-2-methyl-1*H*-benzo[*d*]imidazol-5-yl)-1-(4-sulfamoylphenyl)-5-(*p*-tolyl)-1*H*-pyrazole-3-carboxamide (TFA salt) (**B6**): According to General Procedure A, the Fmoc-benzimidazole-fluorobenzyl linker-preloaded resin PR6 (0.15 g, 0.1 mmol, 1.00 eq., loading: 0.65 mmol/g) was Fmoc-deprotected and subsequently treated with a solution of 1-(4-sulfamoylphenyl)-5-(*p*-tolyl)-1*H*-pyrazole-3-carboxylic acid (0.07 g, 0.2 mmol, 2.00 eq.), HATU (0.08 g, 0.2 mmol, 2.00 eq.), and DIPEA (0.05 mL, 0.3 mmol, 3.00 eq.) in DMF (0.4 mL). The cleavage from the resin was performed with 5 mL of the cleavage cocktail. After purification via preparative HPLC (method C), the desired product B6 (47 mg, 0.06 mmol, 61%) was isolated as a white solid (TFA salt) in a purity of 96%. t_R_ = 15.03 min. ^1^H-NMR (400 MHz, DMSO-*d_6_*): δ = 11.36 (s, 1H), 10.55 (s, 1H), 8.42 (d, *J* = 1.9 Hz, 1H), 7.90 (d, *J* = 8.7 Hz, 2H), 7.86 (dd, *J* = 9.0, 1.9 Hz, 1H), 7.74 (d, *J* = 9.0 Hz, 1H), 7.66 − 7.56 (m, 4H), 7.51 (s, 2H), 7.40 (t, *J* = 7.8 Hz, 1H), 7.23 (s, 4H), 7.17 (s, 1H), 5.79 (s, 2H), 2.82 (s, 3H), 2.32 (s, 3H) ppm. ^13^C-NMR (101 MHz, DMSO-*d_6_*): δ = 162.1, 159.9, 159.8 (d, ^1^*J*_C-F_ = 247.0 Hz), 158.2 (q, ^2^*J*_C-F_ = 32.8 Hz, TFA), 152.3, 147.4, 145.0, 143.7, 141.6, 138.8, 136.4, 135.1 (d, ^3^*J*_C-F_ = 7.2 Hz), 132.0 (d, ^3^*J*_C-F_ = 4.8 Hz), 129.8 (d, ^4^*J*_C-F_ = 3.0 Hz), 129.4 (2C), 128.6 (2C), 128.5, 126.7 (2C), 126.1 (2C), 126.0, 124.5 (d, ^2^*J*_C-F_ = 14.3 Hz), 123.3, 118.7, 116.7 (q, ^1^*J*_C-F_ = 297.5 Hz, TFA), 114.2 (d, ^2^*J*_C-F_ = 23.0 Hz), 112.4, 108.5, 105.6, 42.4, 20.8, 12.1 ppm. ^19^F-NMR (377 MHz, DMSO-*d_6_*): δ = −73.98, −115.66 − −115.78 (m) ppm. HRMS-ESI (*m*/*z*): [M + H]^+^ calc. for C_33_H_28_FN_7_O_5_S: 654.1929; found: 654.1934.

(*E*)-*N*-(4-(3-(Hydroxyamino)-3-oxoprop-1-en-1-yl)benzyl)-1-(4-sulfamoylphenyl)-5-(*p*-tolyl)-1*H*-pyrazole-3-carboxamide (**B7**): According to General Procedure A, the Fmoc-phenylvinyl linker-preloaded resin **PR7** (0.14 g, 0.1 mmol, 1.00 eq., loading: 0.69 mmol/g) was Fmoc-deprotected and subsequently treated with a solution of 1-(4-sulfamoylphenyl)-5-(*p*-tolyl)-1*H*-pyrazole-3-carboxylic acid (0.07 g, 0.2 mmol, 2.00 eq.), HATU (0.08 g, 0.2 mmol, 2.00 eq.), and DIPEA (0.05 mL, 0.3 mmol, 3.00 eq.) in DMF (0.4 mL). The cleavage from the resin was performed with 5 mL of the cleavage cocktail. After purification via preparative HPLC (method D), the desired product **B7** (31 mg, 0.06 mmol, 58%) was isolated as a white solid in a purity of 99%. t_R_ = 16.28 min. ^1^H-NMR (300 MHz, DMSO-*d_6_*): δ = 10.73 (s, 1H), 8.97 (t, *J* = 6.3 Hz, 1H), 7.86 (d, *J* = 8.6 Hz, 2H), 7.53 (dd, *J* = 8.4, 4.3 Hz, 4H), 7.48 (s, 2H), 7.43 (d, *J* = 15.7 Hz, 1H), 7.36 (d, *J* = 8.0 Hz, 2H), 7.20 (d, *J* = 2.9 Hz, 4H), 7.01 (s, 1H), 6.43 (d, *J* = 15.8 Hz, 1H), 4.48 (d, *J* = 6.2 Hz, 2H), 2.31 (s, 3H) ppm. ^13^C-NMR (76 MHz, DMSO-*d_6_*): δ = 162.7, 161.0, 147.6, 144.6, 143.5, 141.6, 141.2, 138.6, 138.1, 133.4, 129.4 (2C), 128.6 (2C), 127.8 (2C), 127.5 (2C), 126.7 (2C), 126.2, 125.8 (2C), 118.6, 108.1, 41.9, 20.8 ppm. HRMS-ESI (*m*/*z*): [M + H]^+^ calc. for C_27_H_25_N_5_O_5_S: 532.1649; found: 532.1636.

*N*-(6-(Hydroxyamino)-6-oxohexyl)-1-(4-(methylsulfonyl)phenyl)-5-(*p*-tolyl)-1*H*-pyrazole-3-carboxamide (**C2**): According to General Procedure A, the Fmoc-pentyl linker-preloaded resin **PR2** (0.14 g, 0.1 mmol, 1.00 eq., loading: 0.70 mmol/g) was Fmoc-deprotected and subsequently treated with a solution of 1-(4-(methylsulfonyl)phenyl)-5-(*p*-tolyl)-1*H*-pyrazole-3-carboxylic acid (0.07 g, 0.2 mmol, 2.00 eq.), HATU (0.08 g, 0.2 mmol, 2.00 eq.), and DIPEA (0.05 mL, 0.3 mmol, 3.00 eq.) in DMF (0.4 mL). The cleavage from the resin was performed with 4 mL of the cleavage cocktail. After purification via preparative HPLC (method B), the desired product **C2** (35 mg, 0.07 mmol, 73%) was isolated as a white solid in a purity of 98%. t_R_ = 16.03 min. ^1^H-NMR (300 MHz, DMSO-*d_6_*): δ = 10.32 (s, 1H), 8.33 (t, *J* = 5.9 Hz, 1H), 7.99 (d, *J* = 8.7 Hz, 2H), 7.60 (d, *J* = 8.7 Hz, 2H), 7.20 (d, *J* = 3.0 Hz, 4H), 6.98 (s, 1H), 3.27 (s, 3H), 3.26 − 3.20 (m, 2H), 2.32 (s, 3H), 1.95 (t, *J* = 7.3 Hz, 2H), 1.51 (p, *J* = 7.3 Hz, 4H), 1.27 (q, *J* = 8.3 Hz, 2H) ppm. ^13^C-NMR (75 MHz, DMSO-*d_6_*): δ = 169.1, 160.7, 148.1, 144.6, 143.1, 140.0, 138.7, 129.5 (2C), 128.6 (2C), 128.1 (2C), 126.1, 125.9 (2C), 108.2, 43.3, 38.5, 32.2, 29.0, 26.0, 24.9, 20.8 ppm. HRMS-ESI (*m*/*z*): [M + H]^+^ calc. for C_24_H_28_N_4_O_5_S: 485.1853; found: 485.1851.

*N*-(7-(Hydroxyamino)-7-oxoheptyl)-1-(4-(methylsulfonyl)phenyl)-5-(*p*-tolyl)-1*H*-pyrazole-3-carboxamide (**C3**): According to General Procedure A, the Fmoc-hexyl linker-preloaded resin **PR3** (0.13 g, 0.1 mmol, 1.00 eq., loading: 0.77 mmol/g) was Fmoc-deprotected and subsequently treated with a solution of 1-(4-(methylsulfonyl)phenyl)-5-(*p*-tolyl)-1*H*-pyrazole-3-carboxylic acid (0.05 g, 0.2 mmol, 1.50 eq.), HATU (0.08 g, 0.2 mmol, 2.00 eq.), and DIPEA (0.05 mL, 0.3 mmol, 3.00 eq.) in DMF (0.3 mL). The cleavage from the resin was performed with 4 mL of the cleavage cocktail. After purification via preparative HPLC (method D), the desired product **C3** (12 mg, 0.02 mmol, 25%) was isolated as a white solid in a purity of 97%. t_R_ = 16.46 min. ^1^H-NMR (400 MHz, DMSO-*d_6_*): δ = 10.32 (s, 1H), 8.34 (t, *J* = 6.0 Hz, 1H), 7.99 (d, *J* = 8.7 Hz, 2H), 7.60 (d, *J* = 8.7 Hz, 2H), 7.24 − 7.16 (m, 4H), 6.98 (s, 1H), 3.27 (s, 3H), 3.26 − 3.20 (m, 2H), 2.32 (s, 3H), 1.94 (t, *J* = 7.3 Hz, 2H), 1.50 (q, *J* = 7.3 Hz, 4H), 1.31 − 1.23 (m, 4H) ppm. ^13^C-NMR (101 MHz, DMSO-*d_6_*): δ = 169.1, 160.7, 148.2, 144.6, 143.1, 140.0, 138.7, 129.5 (2C), 128.6 (2C), 128.1 (2C), 126.1, 125.9 (2C), 108.2, 43.3, 38.5, 32.2, 29.1, 28.4, 26.2, 25.1, 20.8 ppm. HRMS-ESI (*m*/*z*): [M + H]^+^ calc. for C_25_H_30_N_4_O_5_S: 499.2010; found: 499.2017.

*N*-(4-(Hydroxycarbamoyl)benzyl)-1-(4-(methylsulfonyl)phenyl)-5-(*p*-tolyl)-1*H*-pyrazole-3-carboxamide (**C4**): According to General Procedure A, the Fmoc-benzyl linker-preloaded resin **PR4** (0.14 g, 0.1 mmol, 1.00 eq., loading: 0.69 mmol/g) was Fmoc-deprotected and subsequently treated with a solution of 1-(4-(methylsulfonyl)phenyl)-5-(*p*-tolyl)-1*H*-pyrazole-3-carboxylic acid (0.07 g, 0.2 mmol, 2.00 eq.), HATU (0.08 g, 0.2 mmol, 2.00 eq.), and DIPEA (0.05 mL, 0.3 mmol, 3.00 eq.) in DMF (0.4 mL). The cleavage from the resin was performed with 4 mL of the cleavage cocktail. After purification via semi-preparative HPLC (method B), the desired product **C4** (26 mg, 0.05 mmol, 51%) was isolated as a white solid in a purity of 98%. t_R_ = 16.46 min. ^1^H-NMR (400 MHz, DMSO-*d_6_*): δ = 11.17 (s, 1H), 9.02 (t, *J* = 6.3 Hz, 1H), 7.99 (d, *J* = 8.7 Hz, 2H), 7.71 (d, *J* = 8.3 Hz, 2H), 7.61 (d, *J* = 8.7 Hz, 2H), 7.38 (d, *J* = 8.3 Hz, 2H), 7.24 − 7.18 (m, 4H), 7.02 (s, 1H), 4.50 (d, *J* = 6.2 Hz, 2H), 3.27 (s, 3H), 2.32 (s, 3H) ppm. ^13^C-NMR (101 MHz, DMSO-*d_6_*): δ = 164.1, 161.0, 147.8, 144.7, 143.1, 142.9, 140.0, 138.7, 131.3, 129.5 (2C), 128.6 (2C), 128.1 (2C), 127.1 (2C), 126.9 (2C), 126.1, 125.9 (2C), 108.4, 43.3, 41.9, 20.8 ppm. HRMS-ESI (*m*/*z*): [M + H]^+^ calc. for C_26_H_24_N_4_O_5_S: 505.1540; found: 505.1538.

### 3.4. Biological Evaluation

#### 3.4.1. Cell Lines and Cell Culture

The human prostate cancer cell line PC-3, pharynx carcinoma cell line FaDu, and human glioblastoma astrocytoma cell line U87-MG were purchased from the American Type Culture Collection (ATCC). The colorectal adenocarcinoma cell line HT-29 was obtained from the European Collection of Authenticated Cell Cultures (ECACC). The amelanotic melanoma cell line MV3 and the breast cancer cell line MDA-MB-231 were purchased from EPO GmbH Berlin-Buch. The human AsPC1 cell line was provided by Prof. Dr. Ulrich Massing, University Hospital Freiburg, Germany, Clinic for Tumor Biology. Cell culture materials and reagents were purchased from PAN Biotech (Aidenbach, Germany) unless otherwise stated. All cells were incubated in a humidified atmosphere with 5% CO_2_ at 37 °C in RPMI 1640 (PC-3, MV3, AsPC1, FaDu), DMEM (MDA-MB-231, U87-MG) or McCoy´s 5A medium (HT-29) containing 10% fetal bovine serum and 2 mM L-glutamine. Medium was supplemented with 50 U/mL penicillin and 50 µg/mL streptomycin (PC-3, MV3, AsPC1, MDA-MB-231) and 1 mM sodium pyruvate (PC-3, MDA-MB-231). FaDu, HT-29, and the U87-MG cell line medium was purchased from Gibco (Darmstadt, Germany); the corresponding fetal bovine serum was purchased from Sigma Aldrich (St. Louis, MO, USA).

#### 3.4.2. MTT Cell Viability Assay

The intrinsic cytotoxicity of test compounds was determined by a MTT assay as previously described [42,49]. AsPC1, MDA-MB-231, FaDu, HT-29, and U87-MG cells were seeded at a density of 5000 cells/well, and MV3 and PC-3 cells were seeded at a density of 3000 cells/well in 96-well plates (Starlab GmbH, Hamburg, Germany or Greiner Bio-One, Frickenhausen, Germany); all were allowed to attach overnight. Subsequently, cells were exposed to increasing concentrations of the test compounds. After 72 h, MTT solution (5 mg/mL 3-(4,5-dimethylthiazol-2-yl)-2,5-diphenyltetrazolium bromide, in phosphate-buffered saline, Applichem, Darmstadt, Germany or Sigma Aldrich, Steinheim, Germany) was added to determine cell survival. The formazan dye was dissolved in DMSO after 1 h and absorbance was measured at 570 nm and 690, respectively, nm in a Multiskan microplate photometer (Thermo Fisher Scientific, Waltham, MA, USA) and a Cytation 5 Imaging Reader (BioTEK, Santa Clara, CA, USA). Concentration effect curves were generated using nonlinear regression with GraphPad Prism and mean IC_50_ values were calculated based on at least three independent experiments in duplicates.

#### 3.4.3. Apoptosis Assay

PC-3 cells (3 × 10^4^ cells/mL) were seeded into 6-well plates and incubated with different concentrations of compounds or vehicle control (DMSO) for 72 h. Cells were stained with annexin V and propidium iodide and analyzed by flow cytometry (Guava^®^ easyCyte^TM^, Luminex, Austin, TX, USA), according to the manufacturer’s protocol (Catalog#640914, BioLegend, SanDiego, CA, USA). Two independent experiments were performed in triplicates.

Statistical analysis was performed with GraphPad Prism (Graph-Pad Software, San Diego, CA, USA). The results were expressed as mean with standard deviation (SD). The normality of the data was determined using Shapiro-Wilk’s test. A one-way analysis of variance (ANOVA) was performed to determine statistical differences between means. If the difference between means was significant (*p* < 0.05), the mean of each column was compared with the mean of the control and corrected for multiple comparisons using Dunnett’s post-hoc test. Differences were considered statistically significant at * *p* < 0.05, *** *p* < 0.001.

#### 3.4.4. HDAC Whole Cell Assay

Human prostate carcinoma cells PC-3 were seeded in a concentration of 15 × 10^3^ cells/well (total volume of 81 µL) in 96-well cell culture microplates (Catalog# 655086, Greiner Bio-One, Frickenhausen, Germany) and cultured for 24 h. Afterwards, cells were treated with 9 µL decreasing concentrations of the corresponding compound for 18 h. For determination of HDAC activity, 10 µL of substrate solution, containing 3 mM Boc-Lys(ε-Ac)-AMC (Catalog# 233691-67-3, BLD pharmatech GmbH, Kaiserslautern, Germany) and 0.5% IGEPAL CA-630 (Catalog# J61055, Alfa Aesar, Thermo Fisher Scientific, Kandel, Germany), was added and incubated for 3 h under cell culture conditions. The reaction was stopped with 100 µL of stop solution (50 mM Tris-HCl, 137 mM NaCl, 2.7 mM KCl, 1 mM MgCl_2_, 1% IGEPAL CA-630, 10 µM vorinostat, 2.0 mg/mL Trypsin) and developed for 1.5 h under cell culture conditions. The fluorescence signal was measured by Spark multimode microplate reader (Tecan Group AG, Männedorf, Swiss) at excitation of λ = 355 nm and emission of λ = 460 nm. All compounds were tested in three independent experiments in duplicates and IC_50_ values were determined by nonlinear regression with GraphPad Prism. 

#### 3.4.5. Immunoblot

PC-3 cells (7 × 10^4^ cells/mL were treated with 5 µM of the indicated compound or vehicle (DMSO) for 24 h. Cell lysis was performed with Cell Extraction Buffer (10 mM Tris, pH 7.4, 100 mM NaCl, 1 mM EDTA, 1 mM EGTA, 1 mM NaF, 20 mM Na_4_P_2_O_7_, 2 mM Na_3_VO_4_, 1% Triton™ X-100, 10% glycerol, 0.1% SDS, 0.5% deoxycholate (Catalog# FNN0011, Thermo Fisher Scientific Inc., Waltham, MA, USA) and addition of Halt Protease Inhibitor Cocktail (100x) (Catalog# 78429, Life Technologies GmbH, Carlsbad, CA, USA) and phenylmethanesulfonyl fluoride (Catalog# 10837091001, Sigma-Aldrich, St. Louis, MO, USA), according to manufacturer instruction. Protein content was determined by Pierce™ BCA Protein Assay Kit (Catalog# 23225, Thermo Fisher Scientific Inc.) according to manufacturer’s guidelines. Samples were denatured by Leammli 2x Concentrate (Catalog# S3401-10VL, Sigma-Aldrich) and Precision Plus Protein Unstained Standard was used as molecular weight marker (Catalog# 1610363, Bio-Rad, Hercules, CA, USA). SDS-PAGE was performed with 12% Mini-PROTEAN TGX Stain-Free Gel at 200 V for 50 min (Catalog# 4568044, Bio-Rad). Afterwards, proteins were transferred by Trans-Blot Turbo Transfer System (Bio-Rad) to Immobilon-FL PVDF Membrane (Catalog# IPFL00005, Millipore Merck, Burlington, MA, USA) at 1.0 A for 30 min and incubated with 5% milk powder-solution for 1 h at room temperature under slight agitation. Subsequently, the membranes were incubated with anti-acetyl-histone H3 (Catalog# 9677S, Cell Signaling Technology, Denver, MA, USA), anti-acetyl-α-tubulin (Catalog#5335, Cell Signaling Technology), and anti-GAPDH (Catalog# T0004, Affinity Biosciences, Cincinnati, OH, USA) in 1:1000–1:10000 dilutions at 4 °C overnight. Incubation with HRP-conjugated secondary anti-mouse (Catalog# sc-516102, Santa Cruz, Dallas, TX, USA) and anti-rabbit (Catalog# HAF008, R&D Systems, Inc., Minneapolis, MN, USA) was performed for 1.5 h, and membranes were developed with clarity western ECL substrate (Catalog# 1705061, Bio-Rad). ChemiDoc XRS+ System (Bio-Rad) was used for detection and Image Lab Software 6.1 (Bio-Rad) for analyses. Four replicates were performed.

#### 3.4.6. In Vitro HDAC1 and HDAC6 Cell Assay

The inhibition of human HDAC1 and 6 was determined as previously described [50]. OptiPlate-96 black microplates (Perkin Elmer) were used with an assay volume of 50 µL. 5 µL test compound or control, diluted in assay buffer (50 mM Tris-HCl, pH 8.0, 137 mM NaCl, 2.7 mM KCl, 1 mM MgCl_2_, 0.1 mg/mL BSA), were incubated with 35 µL of the fluorogenic substrate ZMAL (Z-Lys(Ac)-AMC) (21.43 µM in assay buffer) and 10 µL of human recombinant HDAC1 (BPS Bioscience, Catalog# 50051) or HDAC6 (BPS Bioscience, Catalog# 50006) at 37 °C. After an incubation time of 90 min, 50 µL of 0.4 mg/mL trypsin in trypsin buffer (50 mM Tris-HCl, pH 8.0, 100 mM NaCl) were added, followed by further incubation at 37 °C for 30 min. Fluorescence was measured with an excitation wavelength of 355 nm and an emission wavelength of 460 nm using a Fluoroskan Ascent microplate reader (Thermo Scientific). All compounds were tested at least twice and in duplicates, and the 50% inhibitory concentration (IC_50_) was determined by plotting dose response curves and nonlinear regression with GraphPad Prism.

#### 3.4.7. Determination of COX Inhibition

The COX inhibition potency against ovine COX-1 and human COX-2 was determined using the fluorescence-based COX assay “COX Fluorescent Inhibitor Screening Assay Kit” (catalog number 700100; Cayman Chemical, Ann Arbor, MI, USA) according to the manufacturer’s instructions as previously reported by us [40,41]. All compounds were assayed in a concentration range of 10 nM to 100 μM in a 10-fold dilutions series, and every inhibitor concentration was assayed in duplicate. If necessary, a narrower concentration range with further concentrations in between was applied to determine IC_50_. Celecoxib was used as internal control. IC_50_ values were estimated using a nonlinear logistic regression fitting procedure (sigmoidal dose−response model) with GraphPad Prism.

## 4. Conclusions

In this work, we have prepared seven different preloaded resins, which were utilized for the solid-phase parallel synthesis of a set of 17 dual HDAC-COX inhibitors. All synthesized compounds were evaluated for the inhibition of COX-1, COX-2, HDAC1, HDAC6, and antiproliferative activity against the PDAC cell line AsPC1. Several of the compounds under study demonstrated a pronounced COX and HDAC inhibitory activity. The selectivity of respective dual inhibitor for the different isoforms turned out to be highly dependent on the nature of the linker and COX inhibitor scaffold used. Whole-cell HDAC inhibition assays and immunoblot analysis confirmed that **C3** and **C4** are capable of inhibiting HDAC activity in a cellular environment. In addition, both **C3** and **C4** caused a significant increase in apoptotic cells, which indicates that the induction of apoptosis contributes to the anticancer properties of **C3** and **C4**. However, at least in the seven cancer cell lines used in this study, the simultaneous inhibition of HDAC and COX activity by dual HDAC-COX inhibitors or combination treatments did not result in additive or synergistic anticancer activities, which disagrees with previous reports. 

## Figures and Tables

**Table 1:** Chemical structures of **A1–A7**, **B1–B7**, and **C2–C4**.

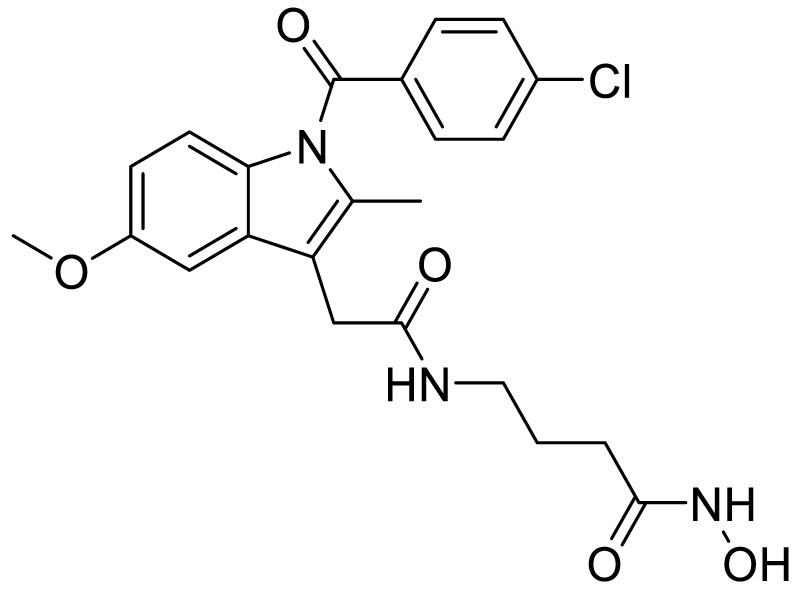 **A1**	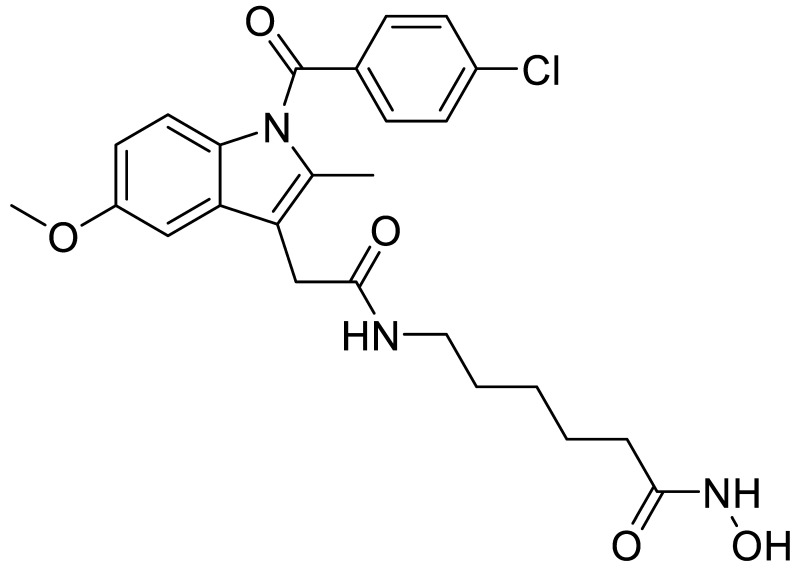 **A2**	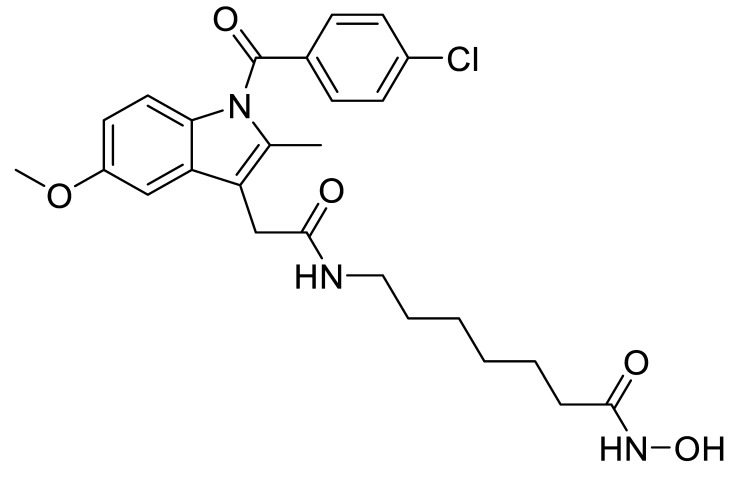 **A3**
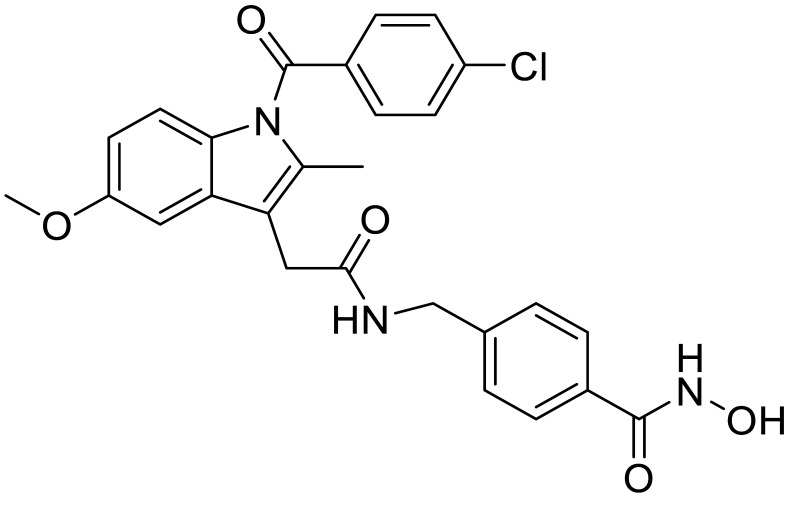 **A4**	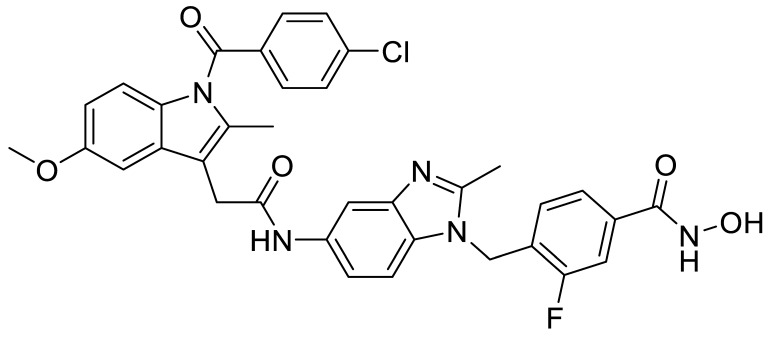 **A5**	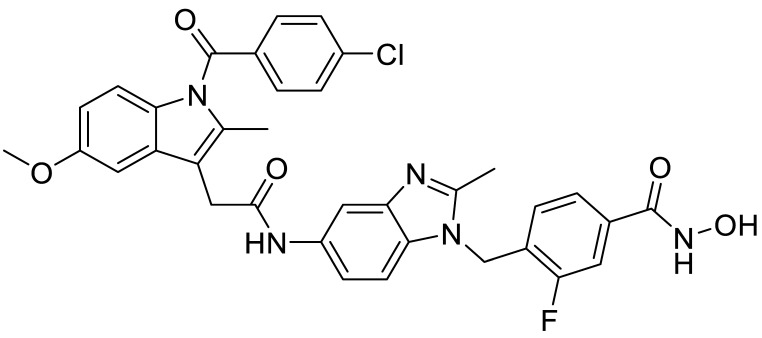 **A6**
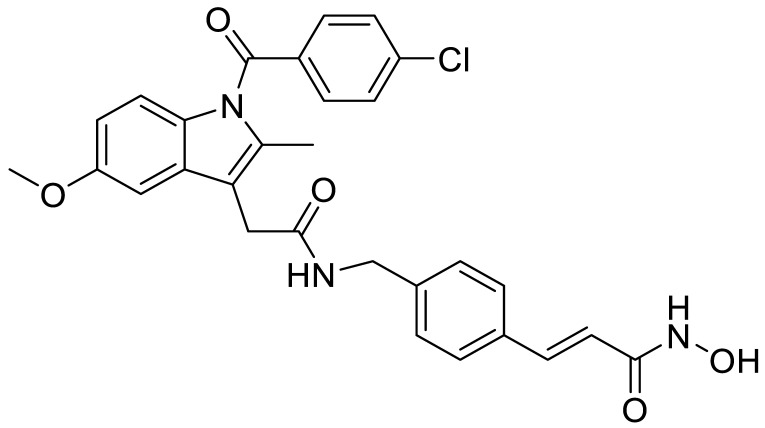 **A7**	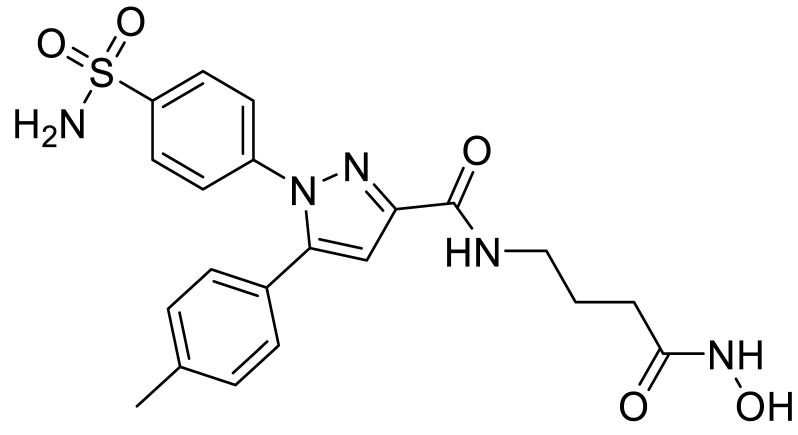 **B1**	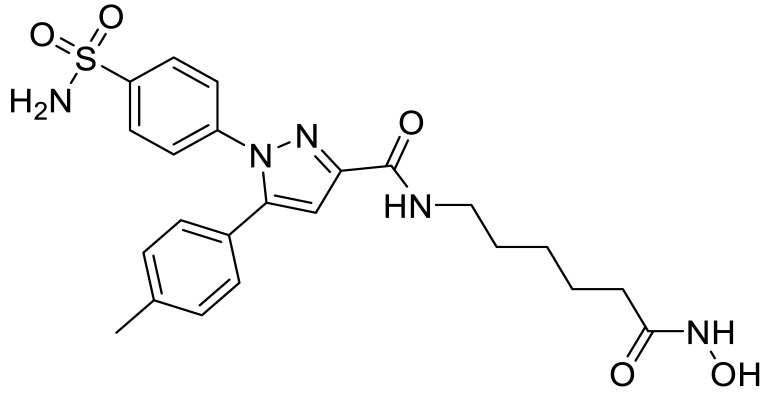 **B2**
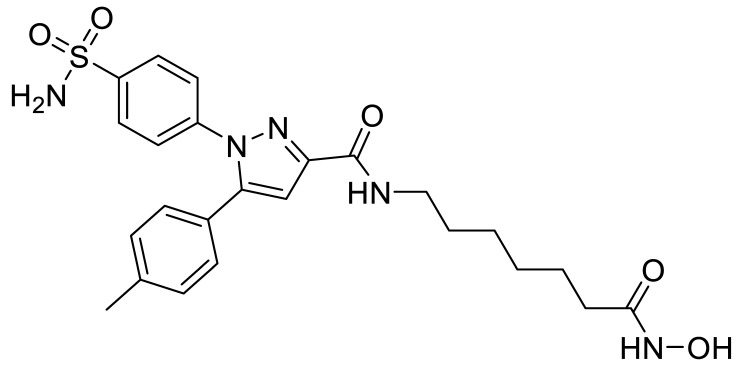 **B3**	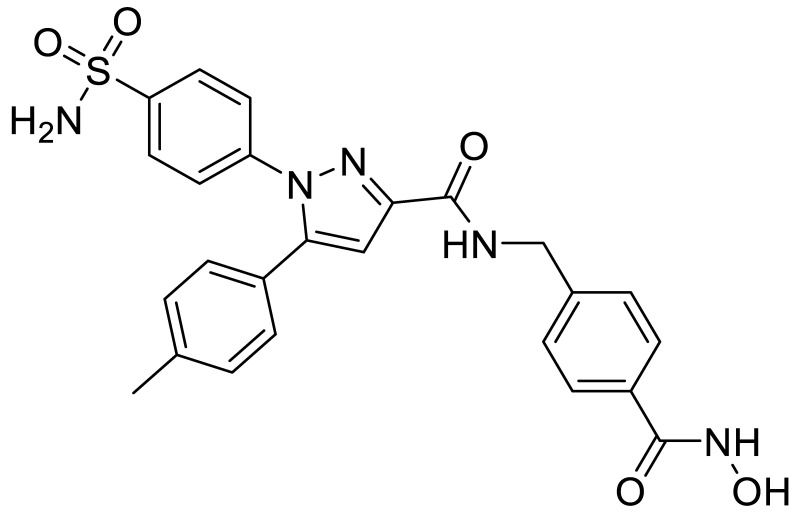 **B4**	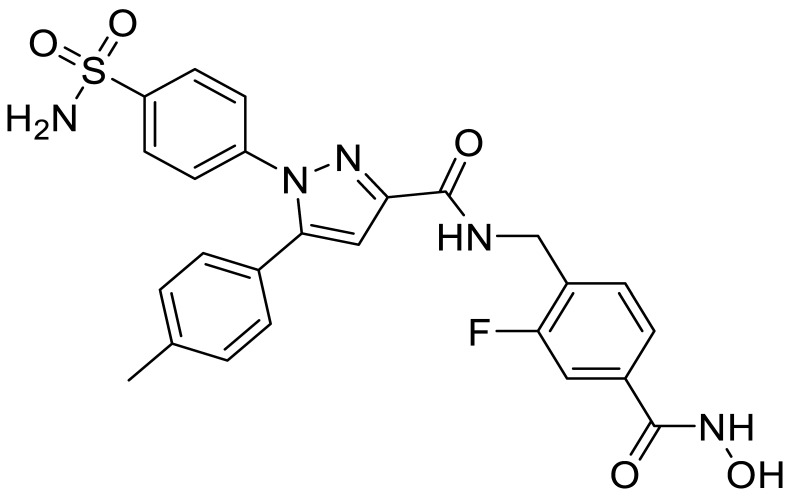 **B5**
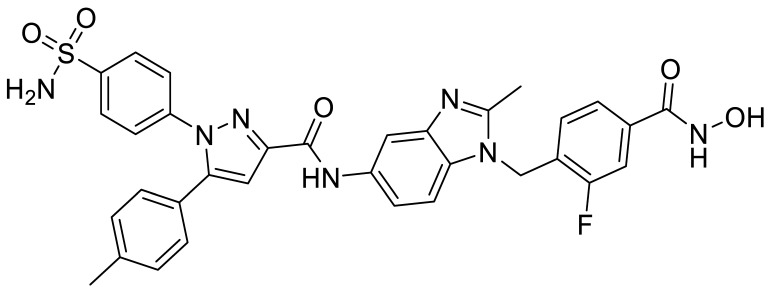 **B6**	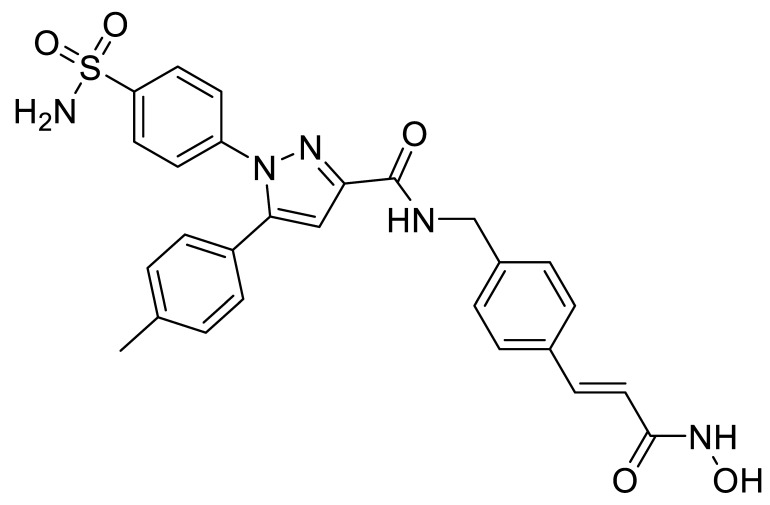 **B7**	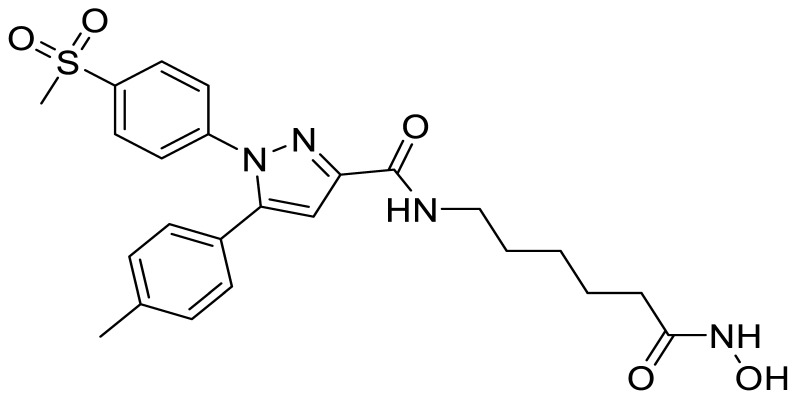 **C2**
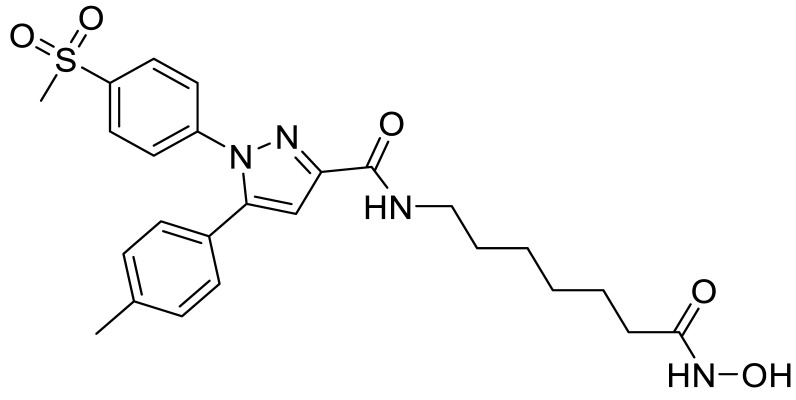 **C3**	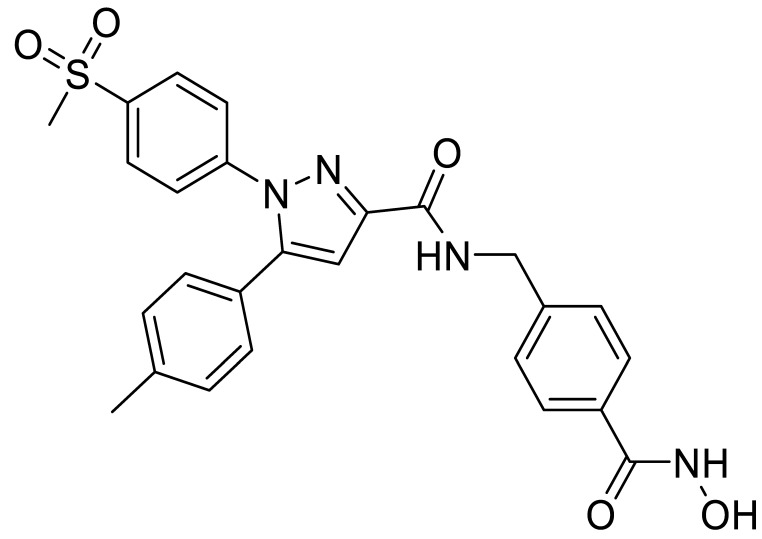 **C4**	

**Table 2:** Inhibitory activity against COX-1, COX-2, HDAC1, HDAC6, and AsPC1 cells as well as log*D* values of **A1–A7**, **B1–B7,** and **C2–C4**.

Cmpd ^1^	COX-1 ^2^ IC_50_ [µM]	COX-2 ^2^ IC_50_ [µM]	SI COX-1/2	HDAC1 IC_50_ [µM]	HDAC6 IC_50_ [µM]	SI HDAC1/6	log*D*_7.4HPLC_^3^	AsPC1 IC_50_ [µM] ^4^
**A1**	>100	5.7	>17	3.990 ± 0.143	3.039 ± 0.147	1.3	2.48	36.46 ± 6.36
**A2**	38.1	4.1	9.3	0.554 ± 0.059	0.055 ± 0.004	10	2.63	10.28 ± 1.95
**A3**	27.2	2.0	13.6	0.377 ± 0.004	0.084 ± 0.012	4	2.76	6.25 ± 1.07
**A4**	18.1	1.2	15.1	>3.33	0.174 ± 0.012	>19	2.89	n.e.
**A5**	6.5	1.6	4.0	>3.33	0.052 ± 0.007	>64	3.00	35.02 ± 5.08
**A6**	2.4	1.9	1.2	>2.80	0.011 ± 0.001	>254	3.14	n.e.
**A7**	3.5	1.8	2.2	0.542 ± 0.052	0.112 ± 0.008	5	3.31	19.20 ± 2.87
**B1**	>100	43.0	>2.3	2.502 ± 0.260	0.695 ± 0.017	4	1.23	n.e.
**B2**	>100	3.3	>30.3	0.099 ± 0.004	0.023 ± 0.0001	4	1.53	45.51 ± 15.83
**B3**	11.6	7.5	1.5	0.034 ± 0.001	0.006 ± 0.00001	6	1.79	3.49 ± 1.29
**B4**	10.8	1.4	7.7	0.039 ± 0.004	0.005 ± 0.0001	8	1.89	15.84 ± 4.26
**B5**	8.8	2.7	3.3	0.155 ± 0.032	0.003 ± 0.0004	52	2.05	n.e.
**B6**	1.8	1.4	1.3	0.829 ± 0.038	0.005 ± 0.0001	166	2.39	n.e.
**B7**	7.5	1.9	3.9	0.014 ± 0.001	0.060 ± 0.011	0.2	2.28	2.54 ± 0.68
**C2**	70.9	3.3	21.5	0.123 ± 0.004	0.038 ± 0.002	3	1.75	59.32 ± 8.47
**C3**	93.8	0.98	95.7	0.043 ± 0.002	0.009 ± 0.001	5	1.99	2.39 ± 0.92
**C4**	11.5	4.1	2.8	0.058 ± 0.001	0.010 ± 0.001	6	2.11	4.28 ± 0.93
**Vorinostat**	n.d.	n.d.	-	0.094 ± 0.013	0.027 ± 0.004	3	n.d.	1.04 ± 0.37
**Cele-coxib**	>100	0.04 ± 0.02 (*n* = 6)	>2500	n.d.	n.d.	n.d.	n.d.	n.e.

^1^ The compound code defines the structure of the respective dual HDAC-COX inhibitor. For instance, compound **A1** contains cap **A** (indometacin) and linker **1** (propyl), see Scheme 1 for the definition of **A–C** and **1–7**. Chemical structure with reference to the compound code is given as IUPAC name in the experimental section and as figures in Table 1. ^2^ All compounds were measured at every concentration in duplicate with the Cytation 5 plate reader and the “COX Fluorescent Inhibitor Screening Assay Kit, Item No. 700,100 (Cayman chemicals, USA)” according to the manufacturer’s instructions. Celecoxib served as reference. The *pIC*_50_ *(pIC_50_ =* -*log_10_ (IC*_50_ *[M]))* for COX-2 was found to be 7.48 ± 0.27 (mean ± SD, *n* = 6; IC_50_ = 38.1 ± 20.7 nM) with an inter-assay coefficient of variation of 3.6%. ^3^ All compounds were measured in duplicate and standard deviation was <0.02 for all compounds. The retention time, mean, and standard deviation of retention time is given in the electronic supporting information (Table S1) for every compound. ^4^ n.e. = no effect up to 100 µM.

**Table 3:** Antiproliferative activity of **A7**, **B7**, **C3**, and **C4** against seven cancer cell lines of different tissue origins. ^1.^

Compound	AsPC1 IC_50_ [µM]	MV3 IC_50_ [µM]	PC-3 IC_50_ [µM]	MDA-MB-231 IC_50_ [µM]	FaDu IC_50_ [µM]	HT-29 IC_50_ [µM]	U-87 IC_50_ [µM]
**A7**	19.20 ± 2.87	20.94 ± 5.75	10.59 ± 1.75	24.45 ± 2.03	6.61 ± 0.74	6.18 ± 1.26	17.46 ± 4.96
**B7**	2.54 ± 0.68	11.59 ± 5.33	7.75 ± 2.30	12.47 ± 4.33	4.79 ± 0.45	6.40 ± 0.67	17.76 ± 2.25
**C3**	2.39 ± 0.92	3.37 ± 0.98	2.67 ± 0.10	3.55 ± 0.62	2.21 ± 0.73	3.56 ± 0.74	6.91 ± 0.31
**C4**	4.28 ± 0.93	9.82 ± 3.02	7.59 ± 1.98	9.57 ± 2.06	3.67 ± 0.50	5.46 ± 0.80	10.63 ± 0.97
**Vorinostat**	1.04 ± 0.37	1.72 ± 0.35	1.40 ± 0.57	1.60 ± 0.46	1.50 ± 0.28	1.58 ± 0.71	4.25 ± 0.84
**Celecoxib**	n.e.	n.e.	n.e.	n.e.	n.e.	n.e.	n.e.
**V + C (1:1) ^2^**	1.21 ± 0.12	1.62 ± 0.34	1.30 ± 0.50	1.46 ± 0.29	1.50 ± 0.41	1.52 ± 0.54	4.94 ± 0.72

^1^ n.e. = no effect up to 100 µM. ^2^ 1:1 combination of vorinostat and celecoxib.

## Data Availability

Data is contained within the article or Supplementary Material.

## Supplementary Materials

The following supporting information can be downloaded at: https://www.mdpi.com/article/10.3390/molecules28031061/s1, Table S1: Retention time, mean of retention time with standard deviation, and log*D* with standard deviation of **A1–A7**, **B1–B7**, and **C2–C4**. NMR spectra and HPLC traces of final compounds.
