# Supplementary material for: Solid-Phase Parallel Synthesis of Dual Histone Deacetylase-Cyclooxygenase Inhibitors
Source: Molecules. 2023 Jan 20;28(3):1061. doi: 10.3390/molecules28031061 (PMC9920637; doi:10.3390/molecules28031061)
Supplement: Supplementary file 1 [file molecules-28-01061-s001.zip › molecules-2144174-supplementary.pdf]

Electronic Supplementary Information

Solid-Phase Parallel Synthesis of Dual Histone Deacetylase-Cyclooxygenase Inhibitors

Luisa M. Bachmann[1], Maria Hanl[2], Felix Feller[2], Laura Sinatra[1], Andrea Schöler[1], Jens Pietzsch[3,4], Markus Laube[3,*] and Finn K. Hansen[2,*]

[1] Institute for Drug Discovery, Medical Faculty, Leipzig University, Brüderstraße 34, D-04103 Leipzig, Germany
[2] Pharmaceutical Institute, Department of Pharmaceutical and Cell Biological Chemistry, University of Bonn, An der Immenburg 4, D-53121 Bonn, Germany
[3] Helmholtz-Zentrum Dresden-Rossendorf, Institute of Radiopharmaceutical Cancer Research, Department of Radiopharmaceutical and Chemical Biology, Bautzner Landstraße 400, D-01328, Dresden, Germany
[4] Technische Universität Dresden, School of Science, Faculty of Chemistry and Food Chemistry, Mommsenstraße 4, D-01062 Dresden, Germany
* Correspondence: m.laube@hzdr.de (M.L.); finn.hansen@uni-bonn.de (F.K.H)

Table of Contents

1. Supplemental Table……………………………………………………………………………..S3
2. NMR Spectra……………………………….…….....…………………………………….…….…….……S4
3. HPLC Chromatograms………………………….....….……………………………………..……..S24

1. Supplemental Table

Table S1. Retention time, mean of retention time with standard deviation (t_R ± SD), and log*D* with standard deviation of **A1–A7**, **B1–B7**, and **C2–C4**.

Compound	t_R [min]	t_R ± SD [min]	log*D* ± SD
**A1**	15.46 / 15.49	15.47 ± 0.02	2.48 ± 0
**A2**	16.15 / 16.17	16.16 ± 0.01	2.63 ± 0
**A3**	16.75 / 16.80	16.77 ± 0.03	2.76 ± 0.01
**A4**	17.44 / 17.29	17.36 ± 0.07	2.89 ± 0.02
**A5**	17.77 / 17.98	17.87 ± 0.1	3.00 ± 0.02
**A6**	18.52 / 18.56	18.54 ± 0.02	3.14 ± 0
**A7**	18.46 / 18.55	18.5 ± 0.05	3.31 ± 0.01
**B1**	9.61 / 9.64	9.62 ± 0.01	1.23 ± 0
**B2**	11.04 / 11.05	11.04 ± 0	1.53 ± 0
**B3**	12.22 / 12.28	12.25 ± 0.03	1.79 ± 0.01
**B4**	12.73 / 12.70	12.71 ± 0.01	1.89 ± 0
**B5**	13.46 / 13.48	13.47 ± 0.01	2.05 ± 0
**B6**	15.01 / 15.08	15.04 ± 0.03	2.39 ± 0.01
**B7**	14.52 / 14.49	14.5 ± 0.01	2.28 ± 0
**C2**	12.02 / 12.07	12.05 ± 0.02	1.75 ± 0
**C3**	13.20 / 13.18	13.19 ± 0.01	1.99 ± 0
**C4**	13.75 / 13.71	13.73 ± 0.02	2.11 ± 0

2. NMR Spectra

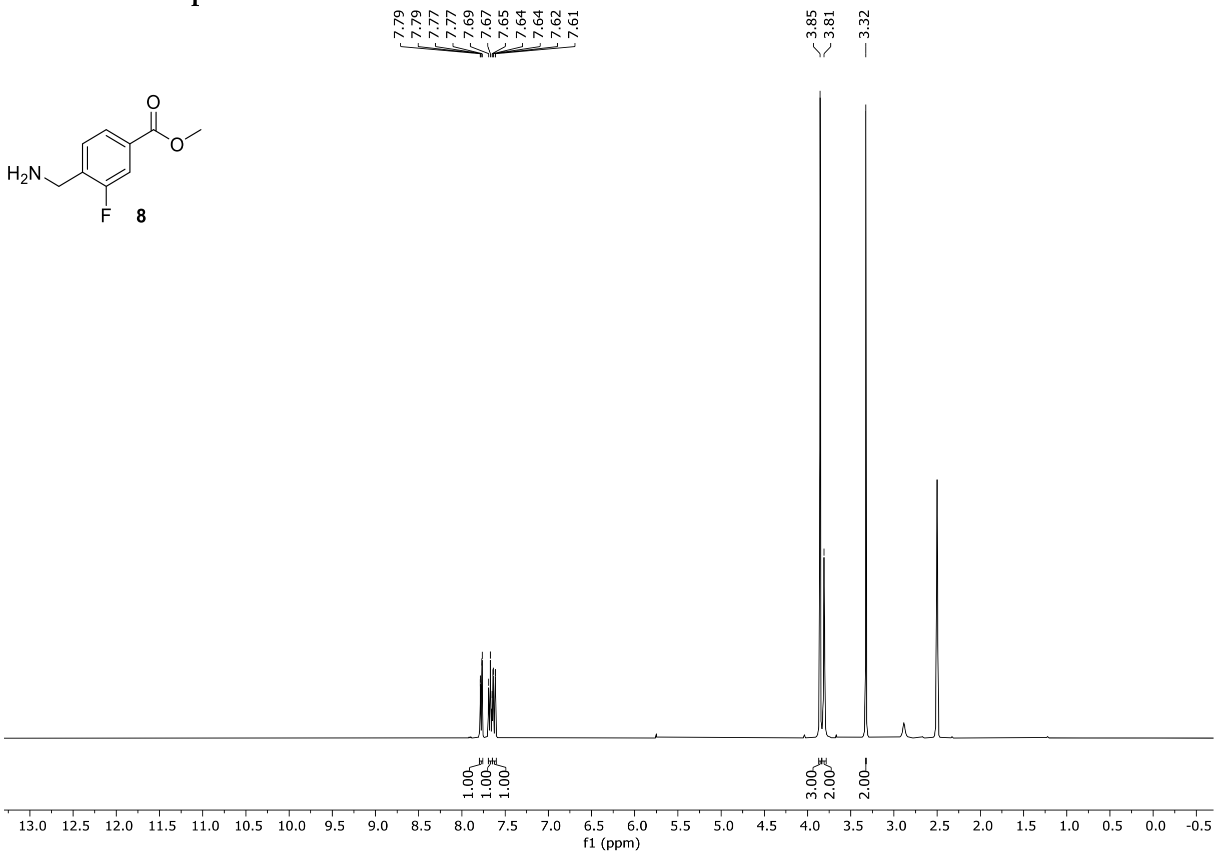

Figure S1. ^{1}H-NMR spectrum of **8** in DMSO-d_6.

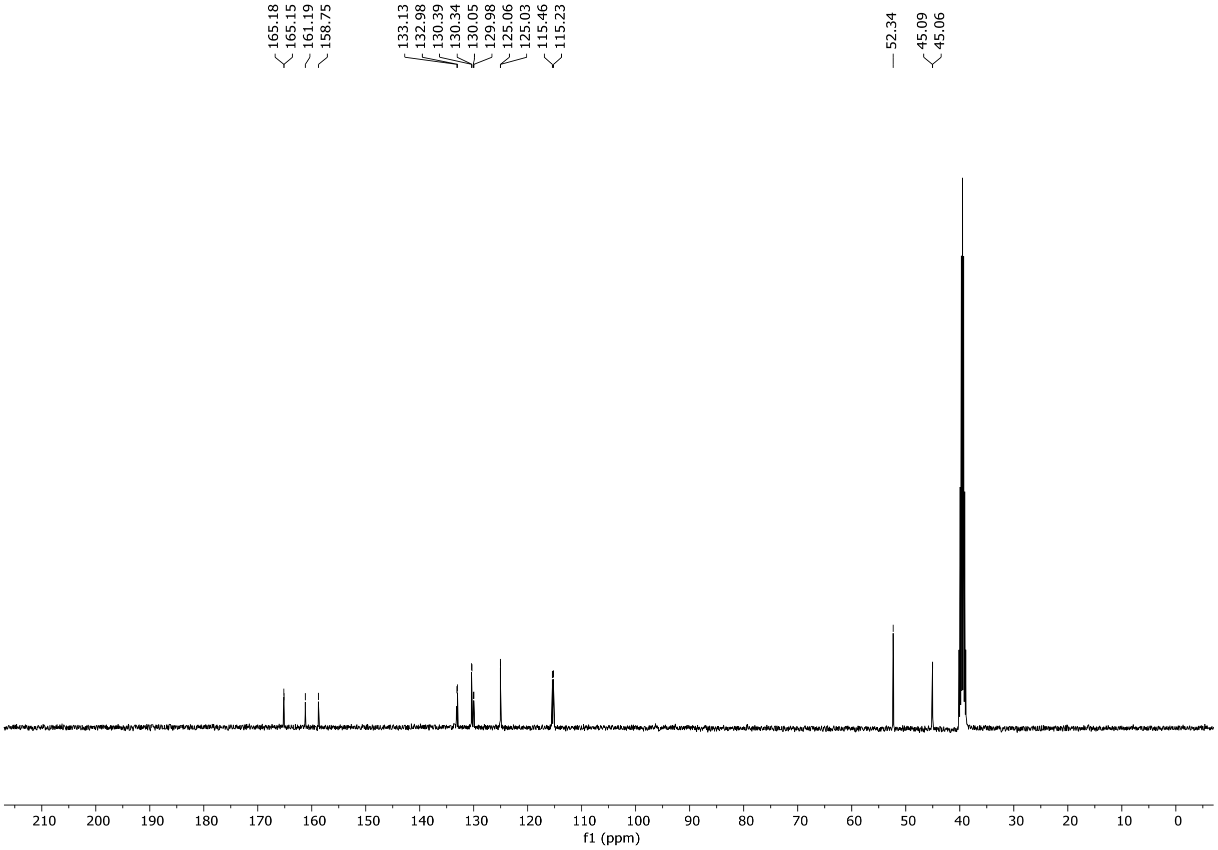

Figure S2. ^{13}C-NMR spectrum of **8** in DMSO-d_6.

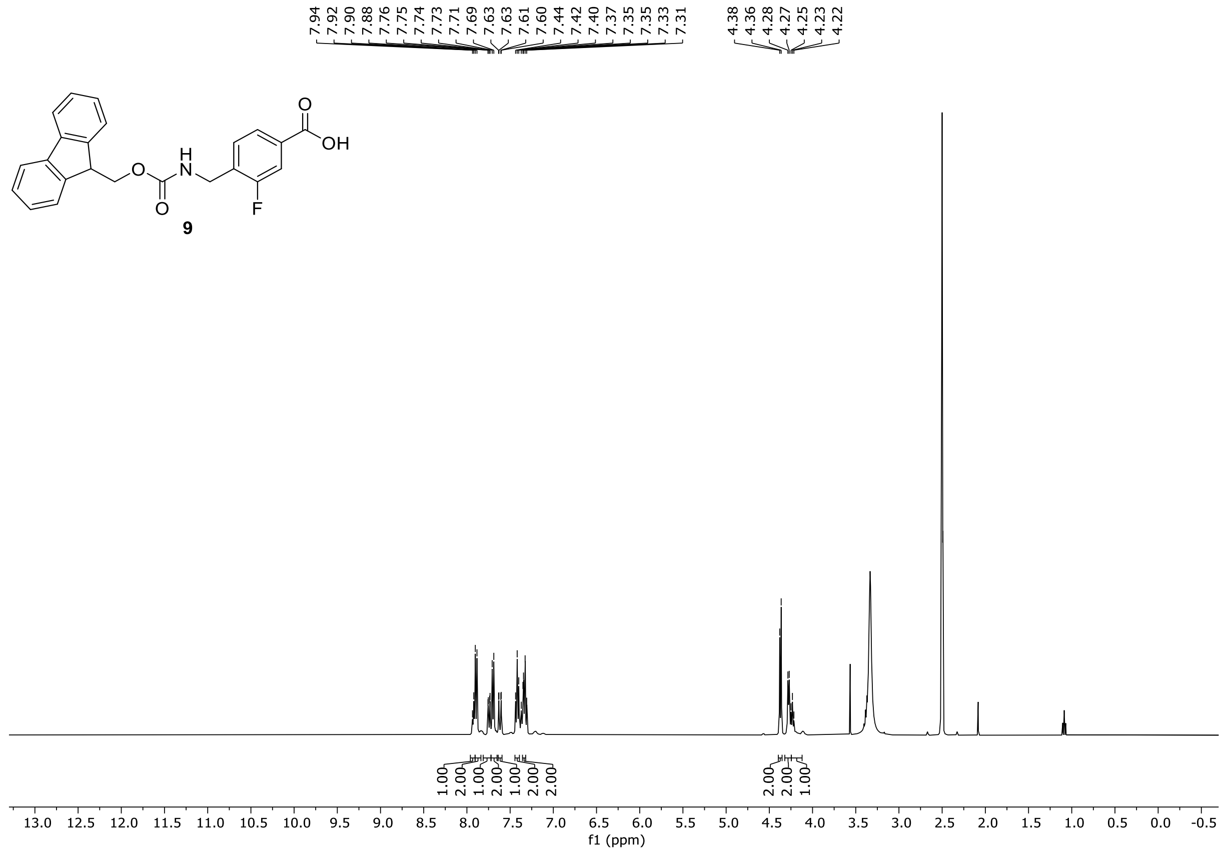

Figure S3. ^{1}H-NMR spectrum of **9** in DMSO-d_6.

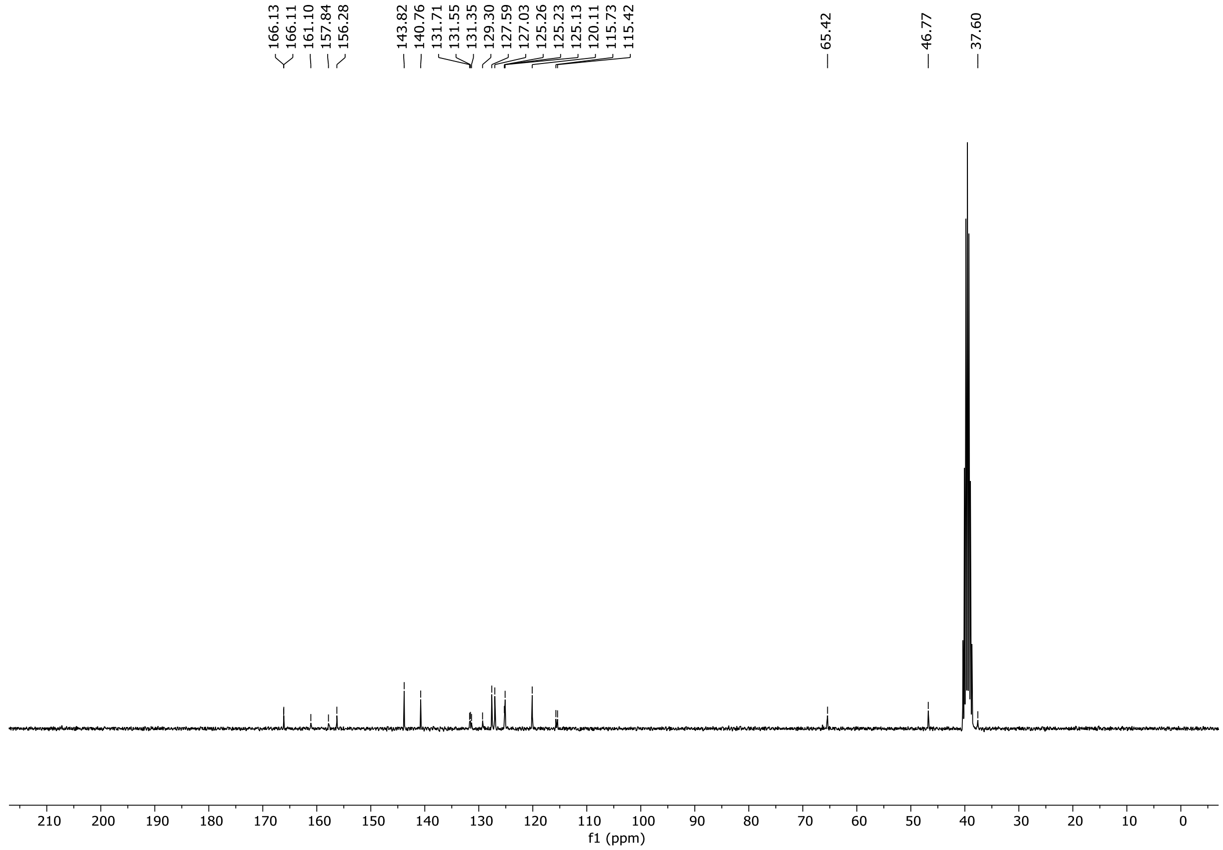

Figure S4. ^{13}C-NMR spectrum of **9** in DMSO-d_6.

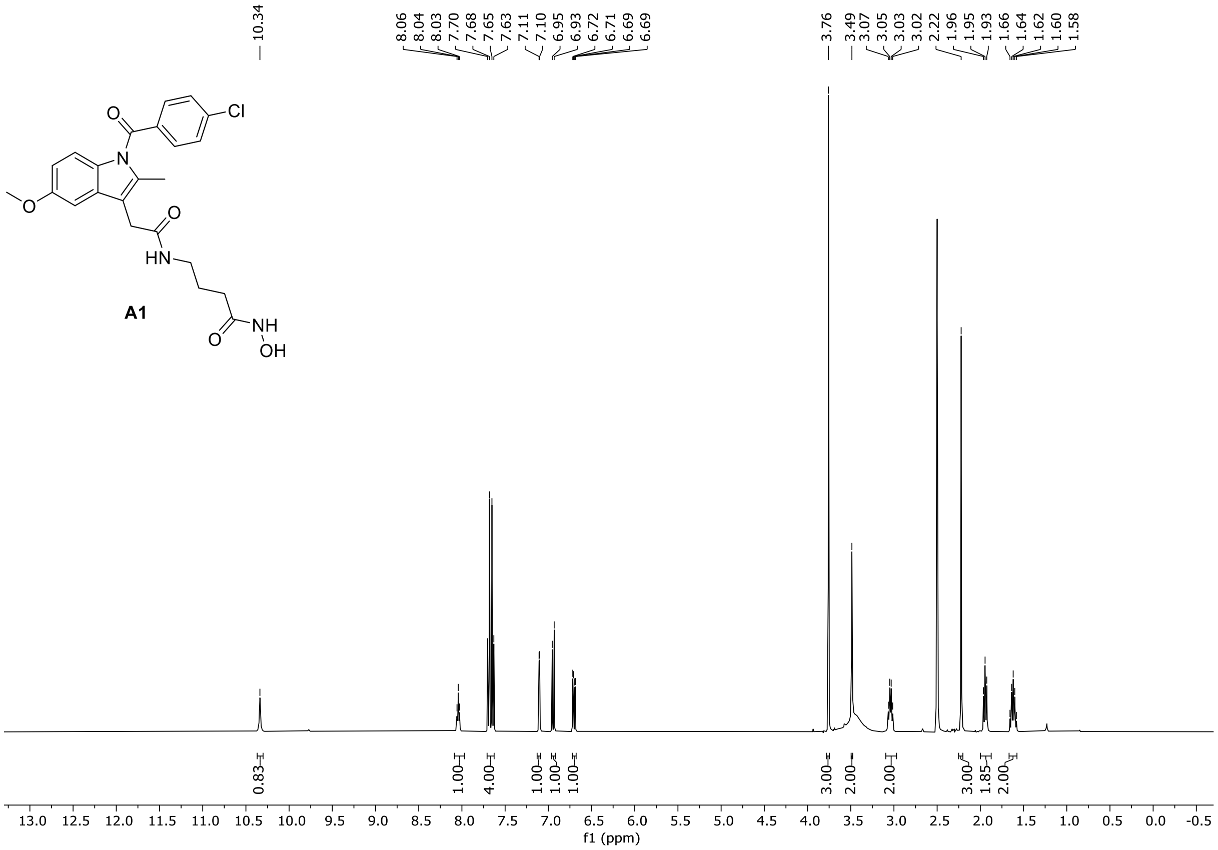

Figure S5. ^{1}H-NMR spectrum of **A1** in DMSO-d_6.

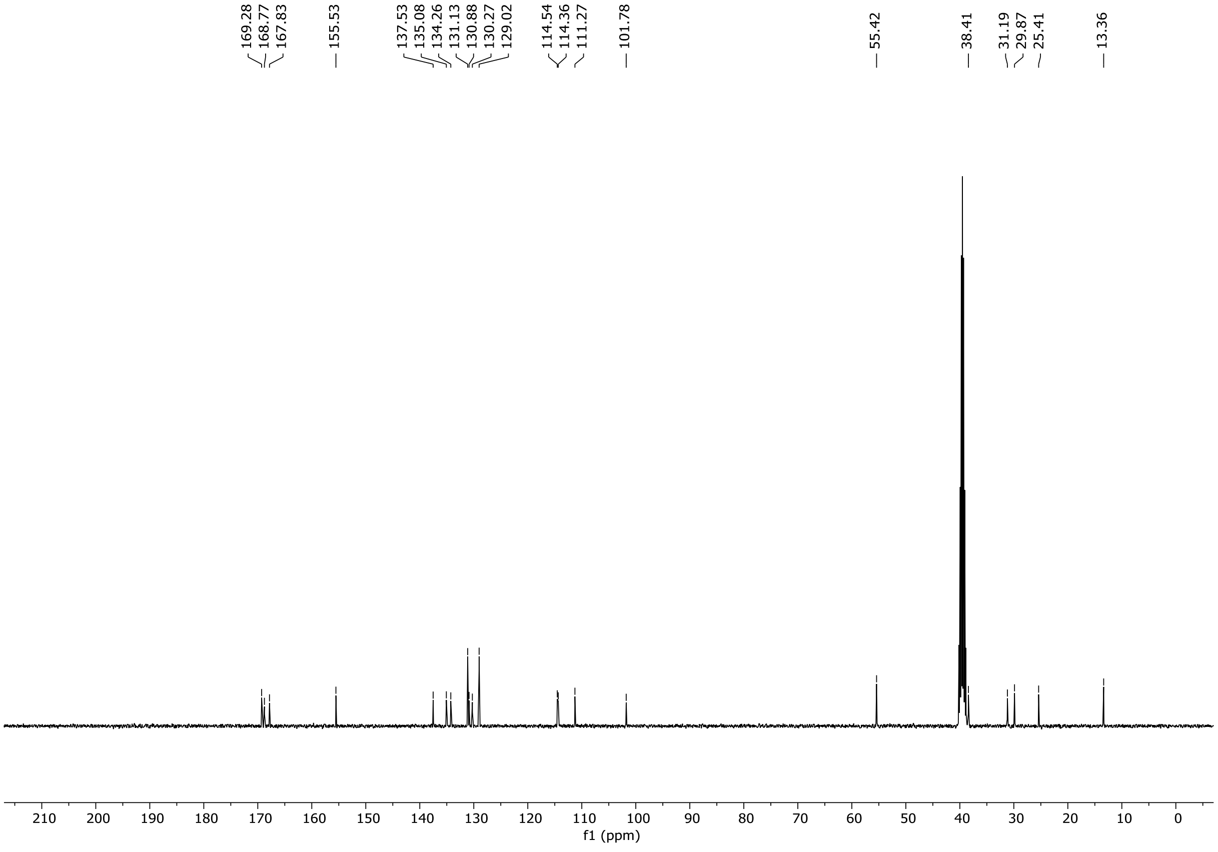

Figure S6. ^{13}C-NMR spectrum of **A1** in DMSO-d_6.

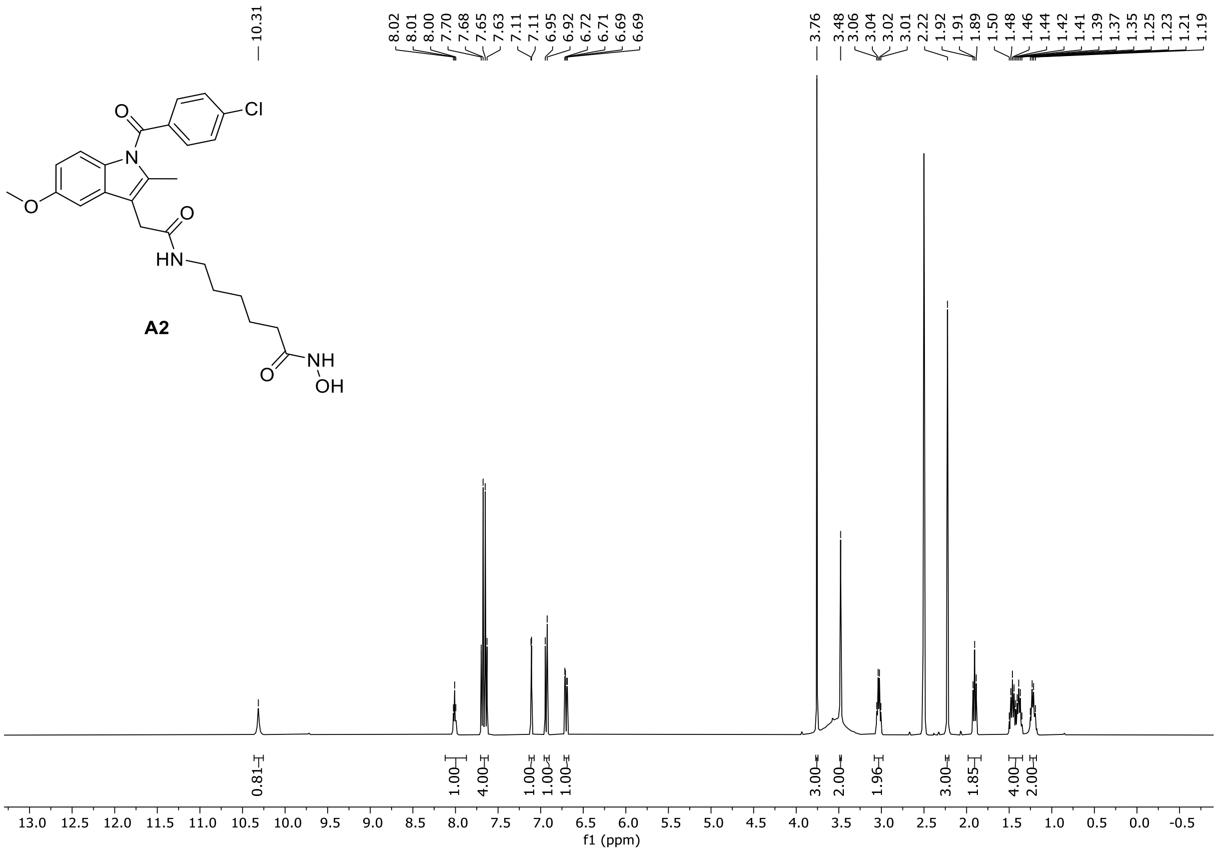

Figure S7. ^{1}H-NMR spectrum of **A2** in DMSO-d_6.

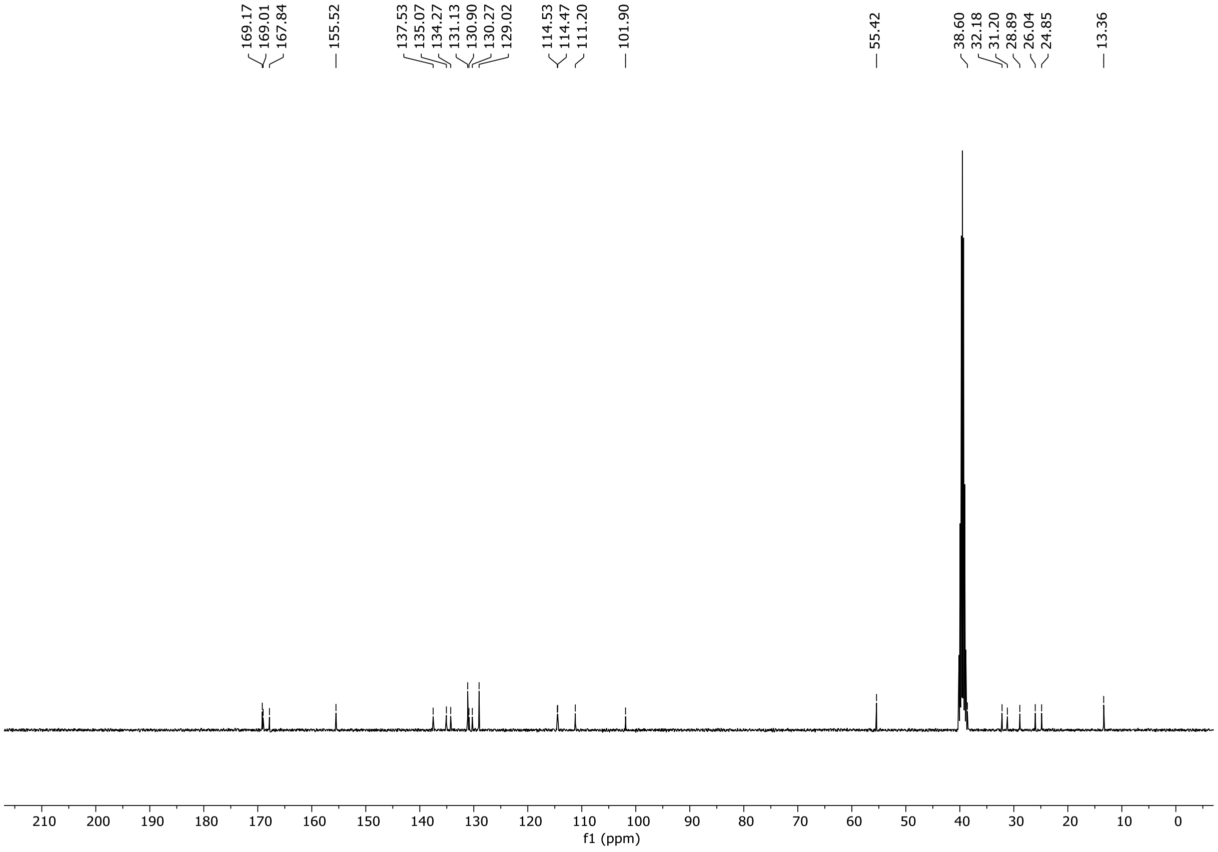

Figure S8. ^{13}C-NMR spectrum of **A2** in DMSO-d_6.

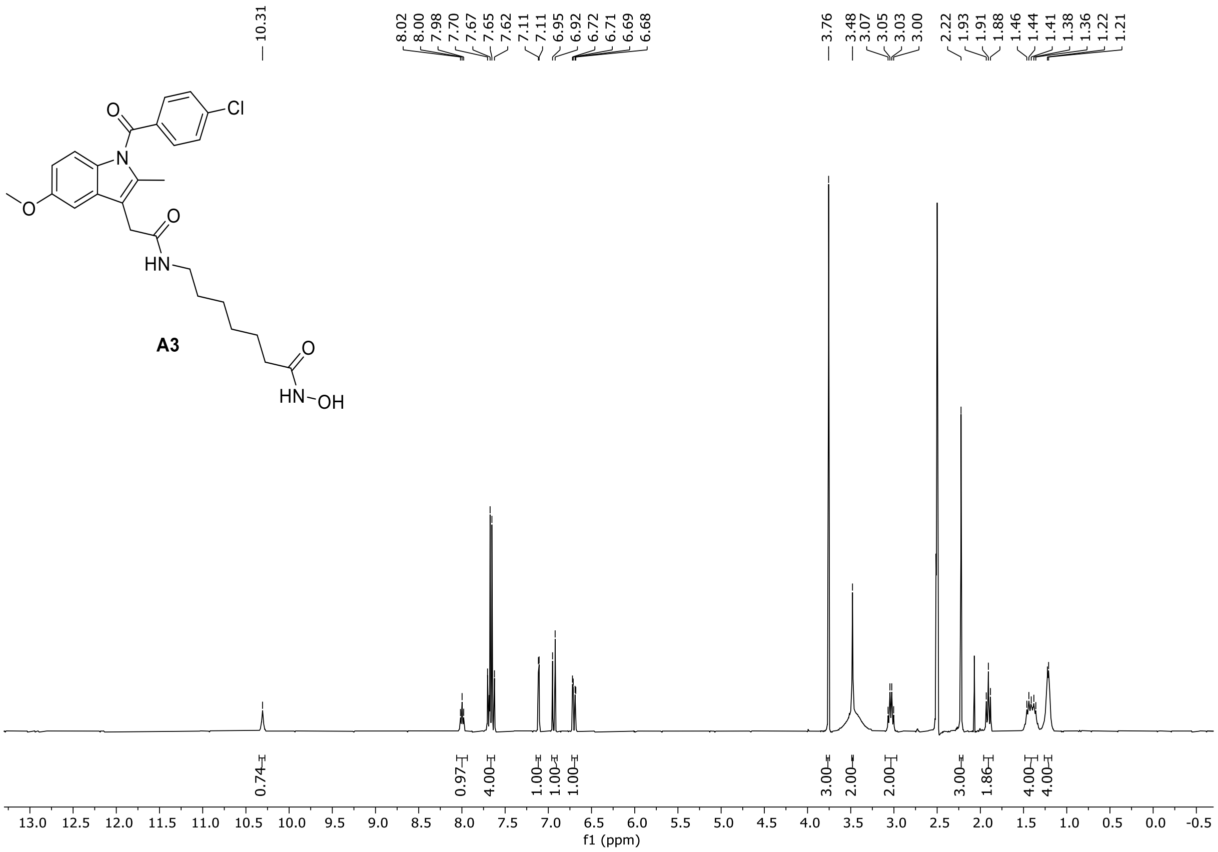

Figure S9. ^{1}H-NMR spectrum of **A3** in DMSO-d_6.

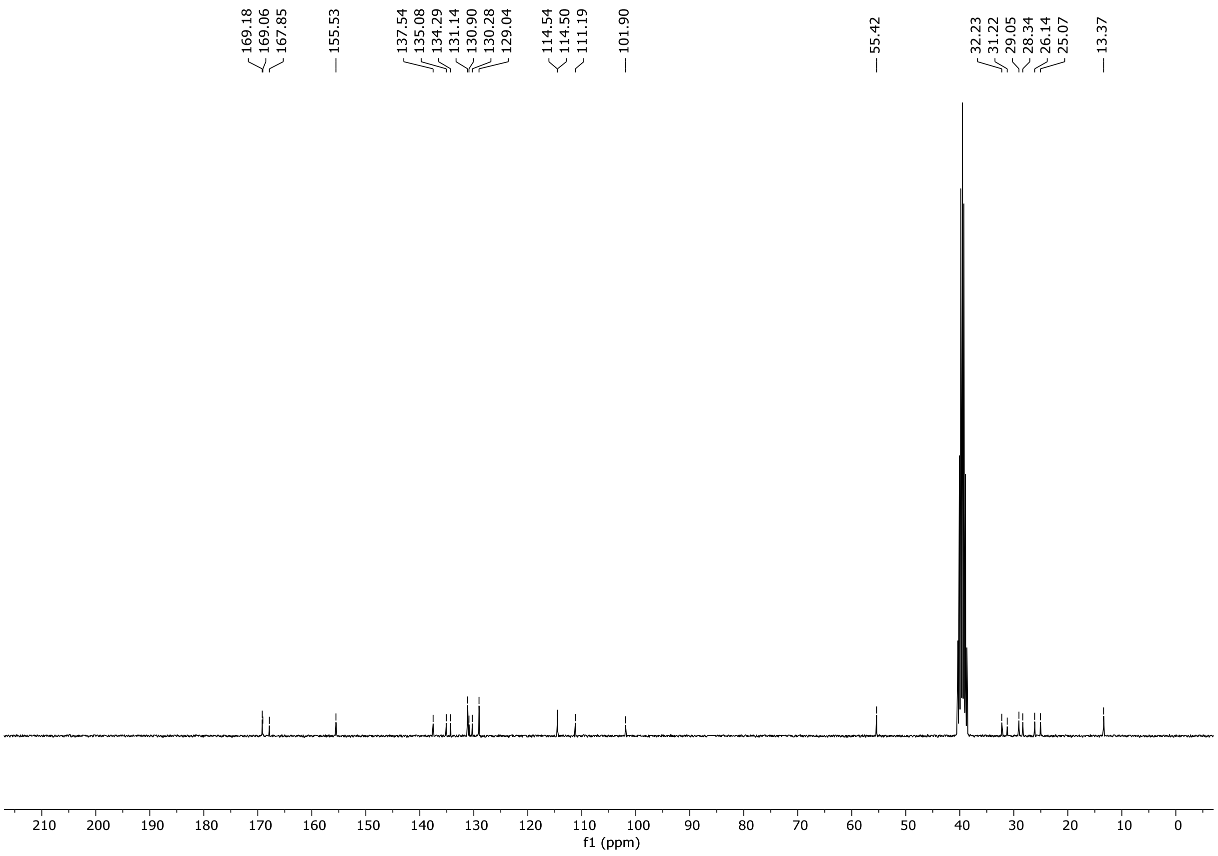

Figure S10. ^{13}C-NMR spectrum of **A3** in DMSO-d_6.

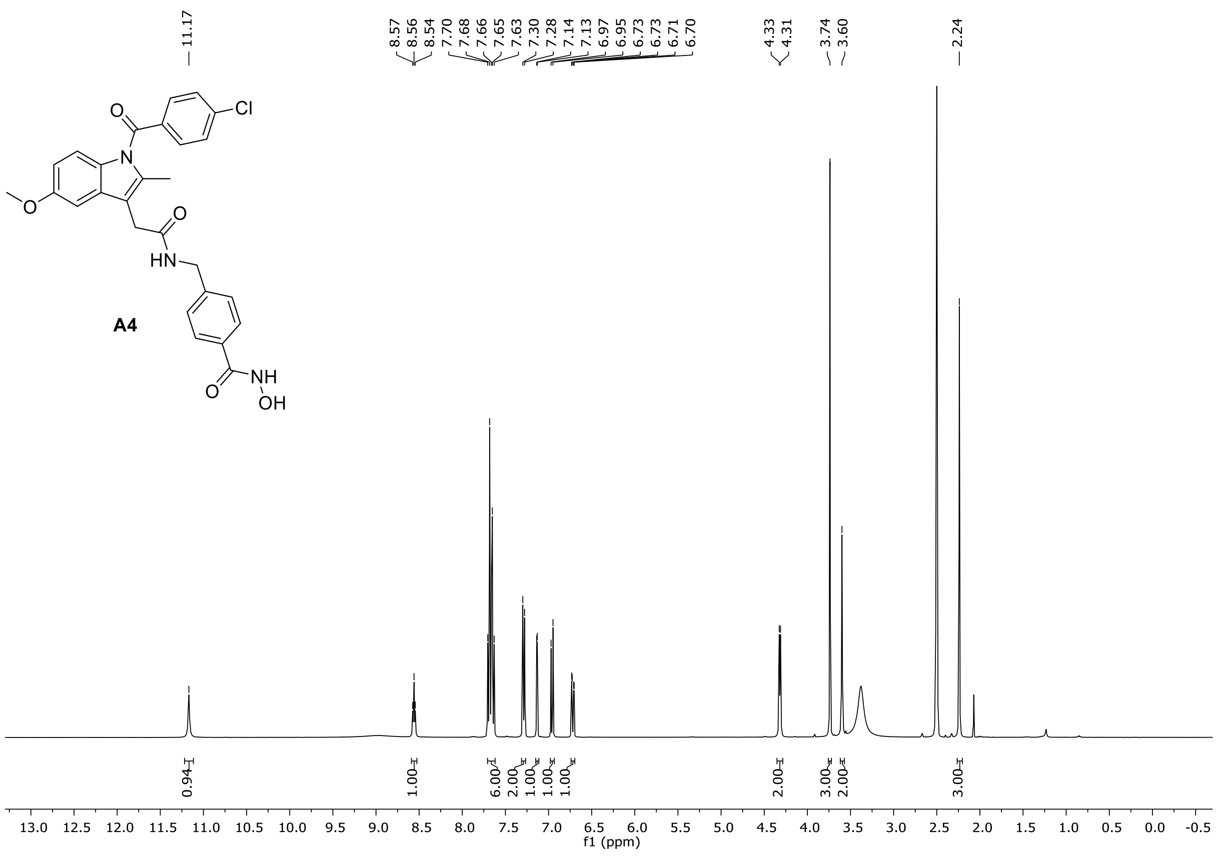

Figure S11. ^{1}H-NMR spectrum of **A4** in DMSO-*d*$_6$.

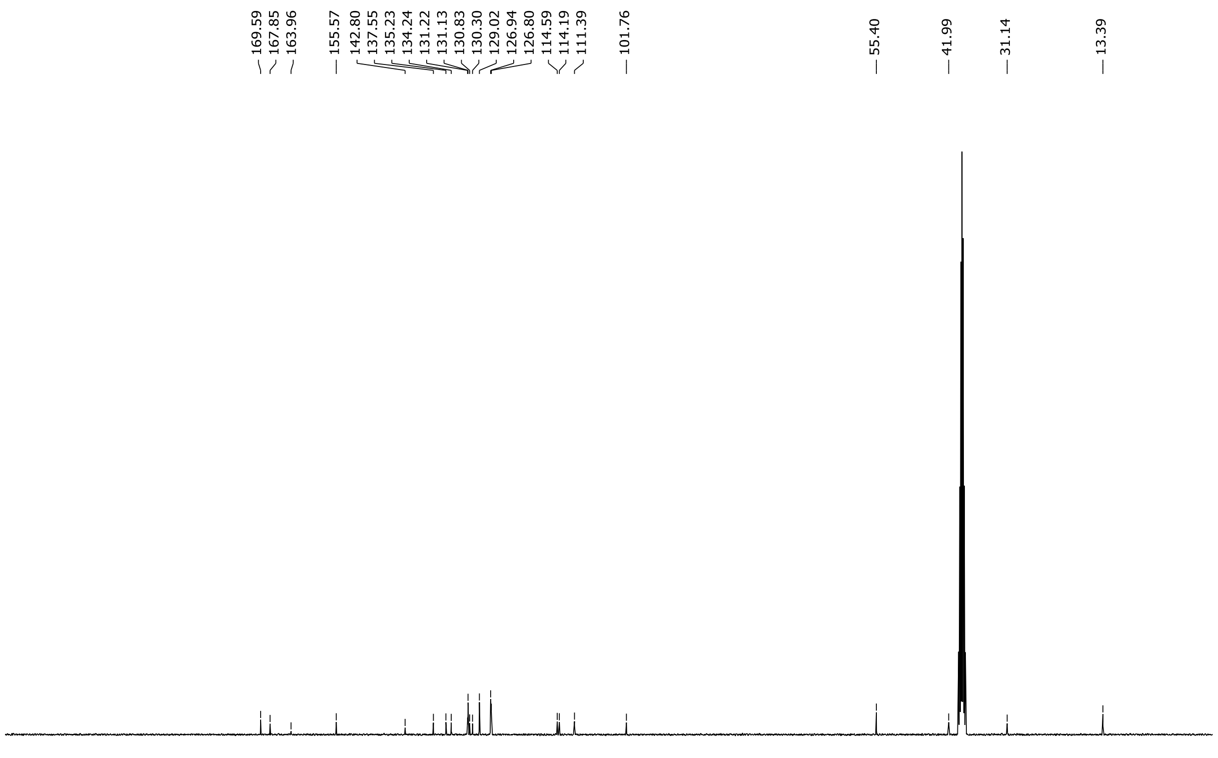

Figure S12. ^{13}C-NMR spectrum of **A4** in DMSO-*d*$_6$.

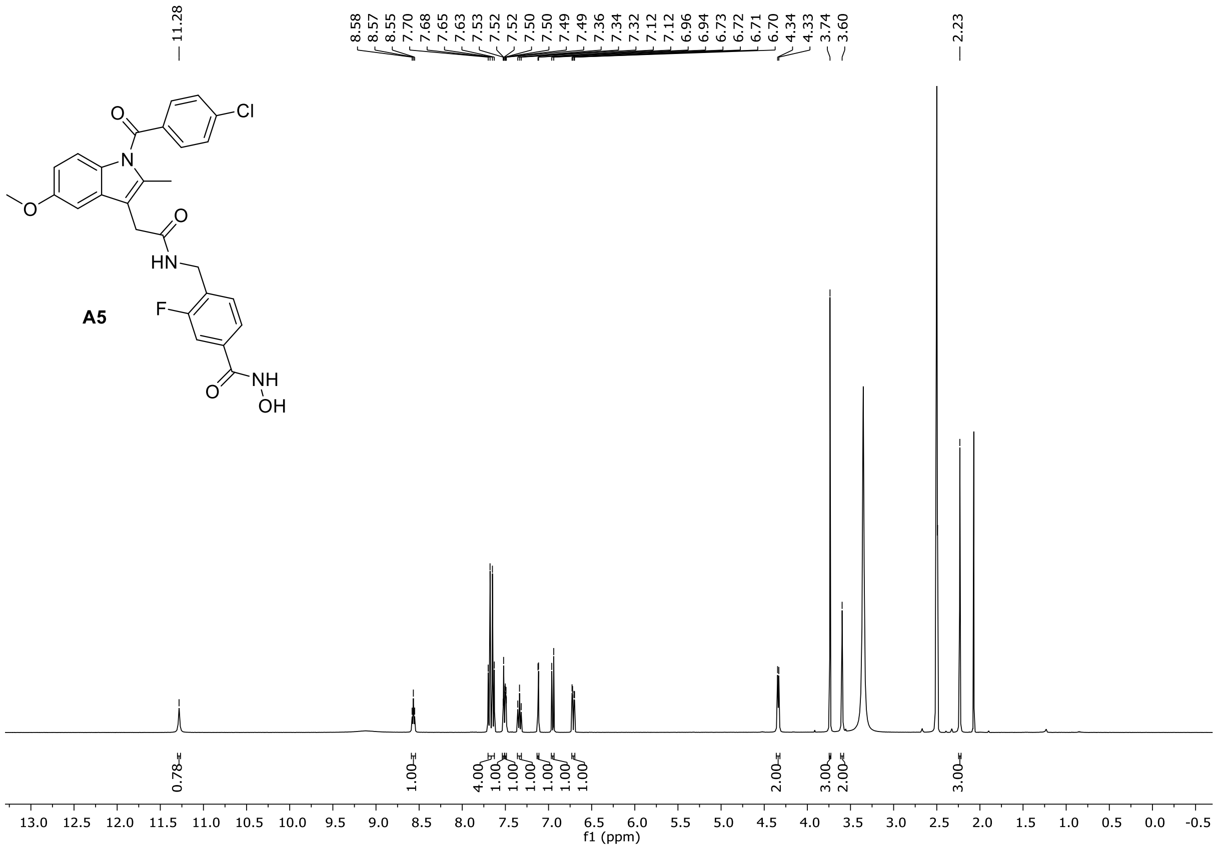

Figure S13. ^{1}H-NMR spectrum of **A5** in DMSO-d_6.

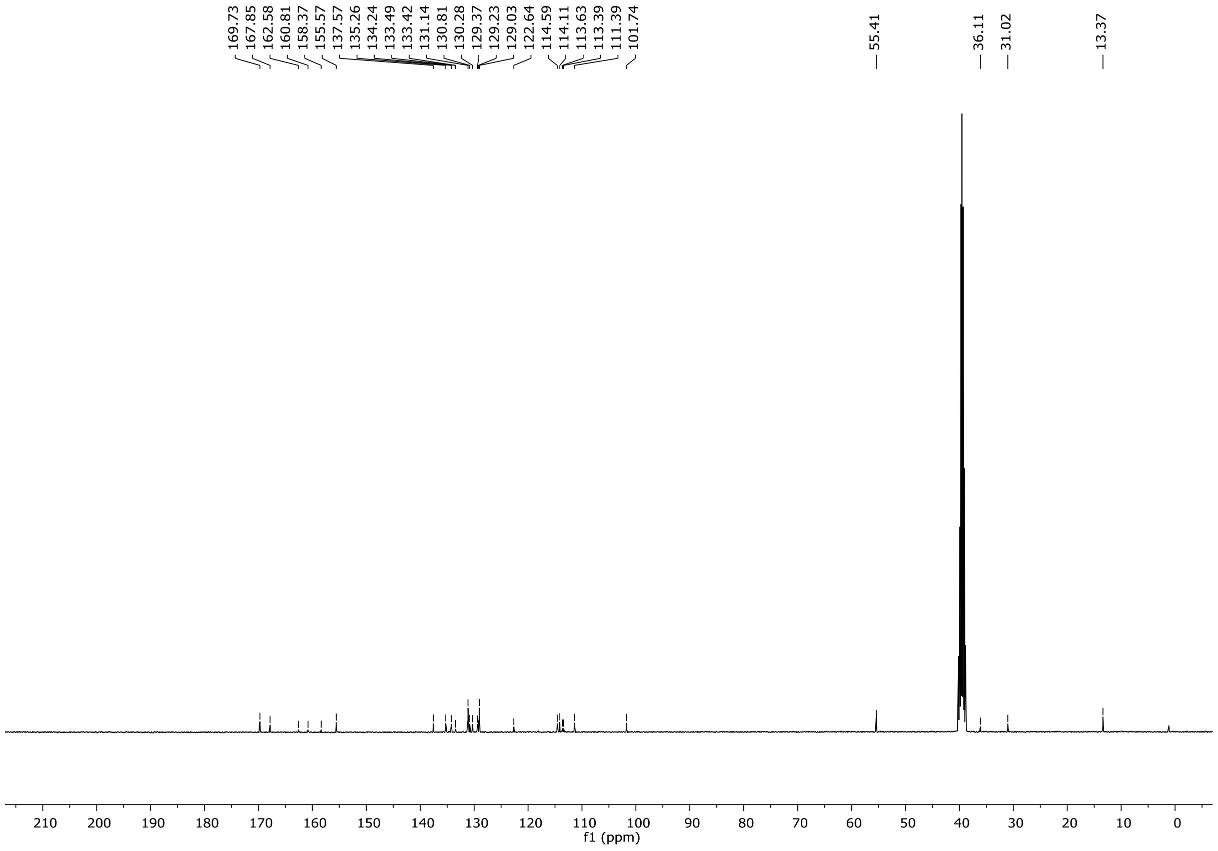

Figure S14. ^{13}C-NMR spectrum of **A5** in DMSO-d_6.

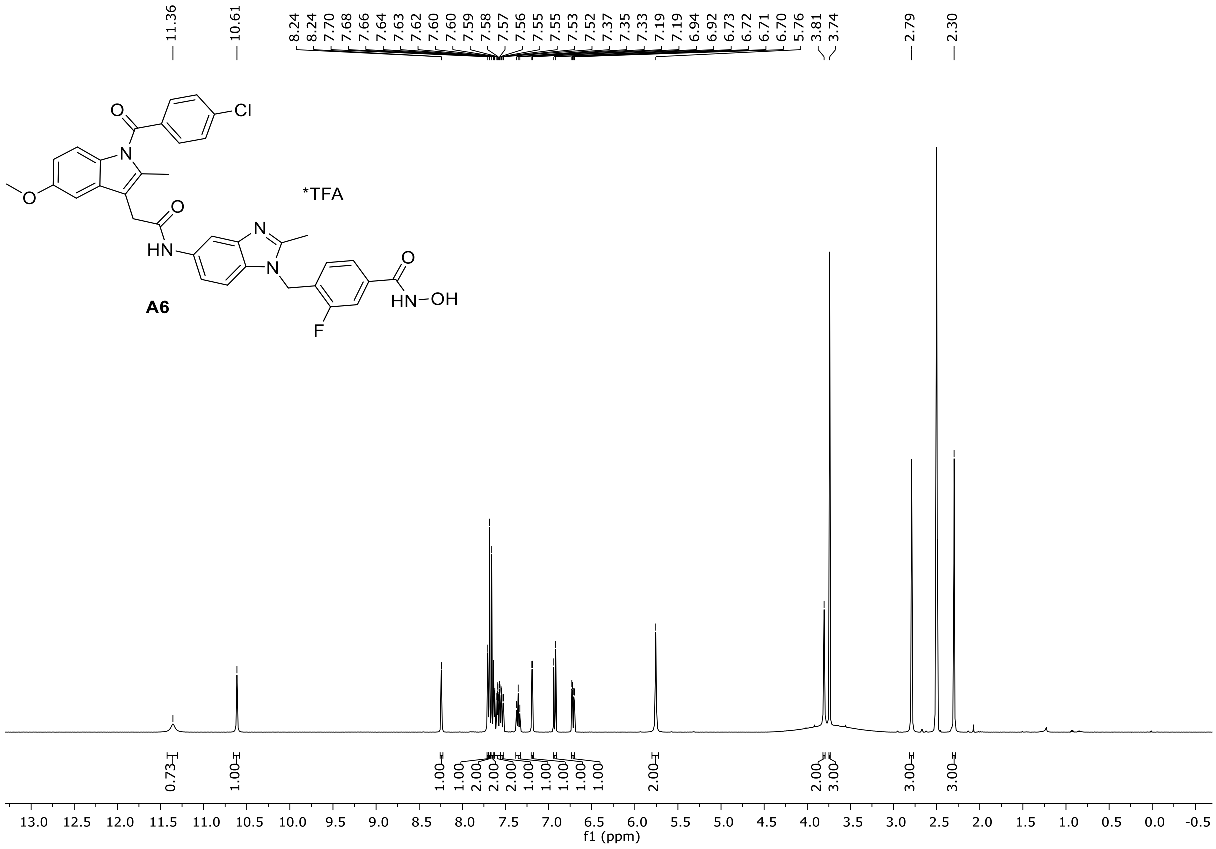

Figure S15. ^{1}H-NMR spectrum of **A6** in DMSO-d_6.

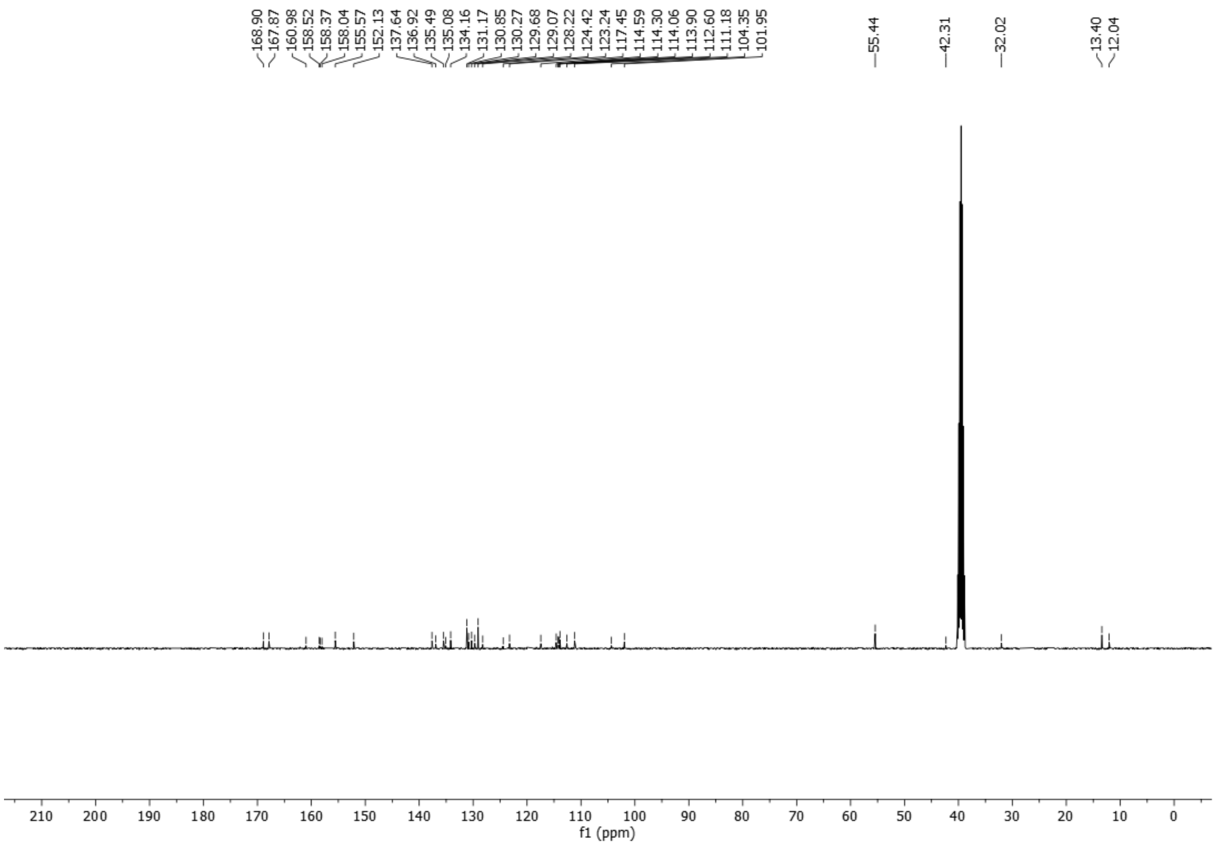

Figure S16. ^{13}C-NMR spectrum of **A6** in DMSO-d_6.

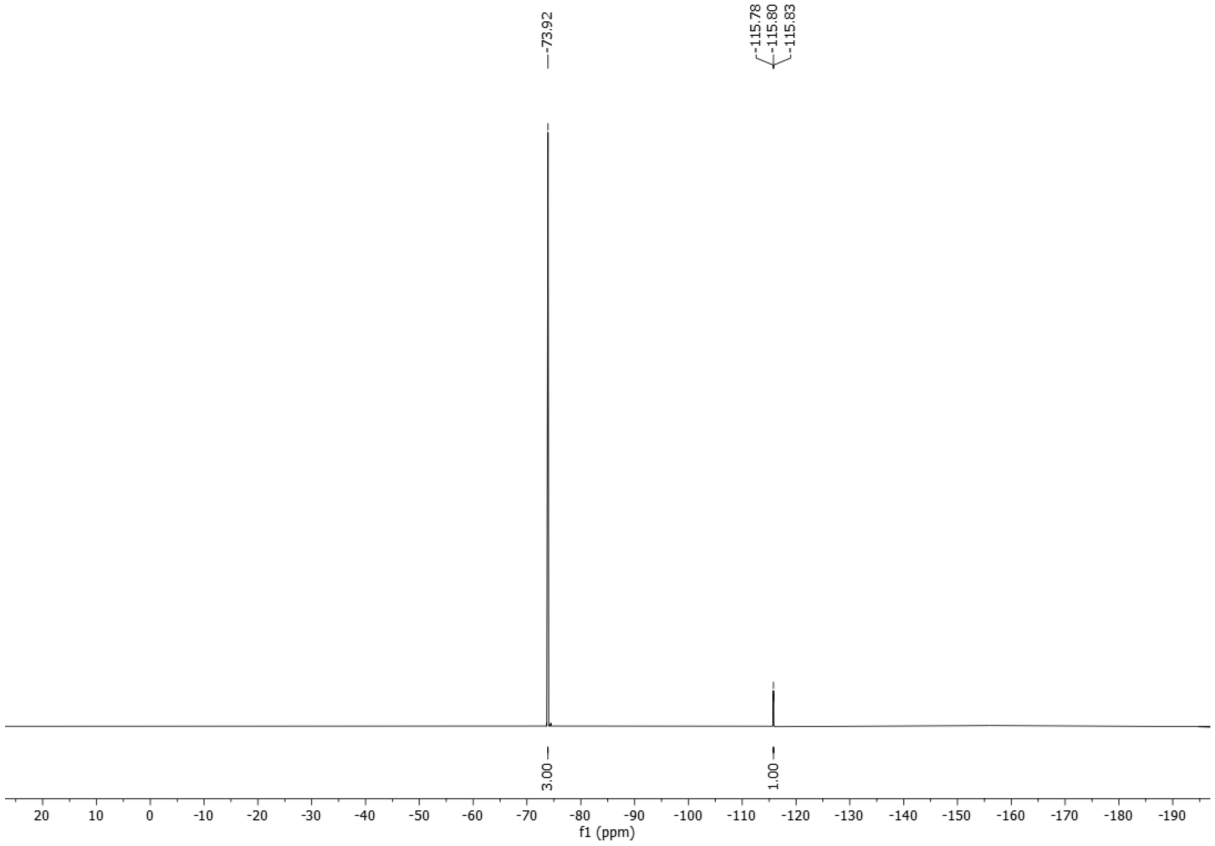

Figure S17. ^{19}F-NMR spectrum of **A6** in DMSO-d_6.

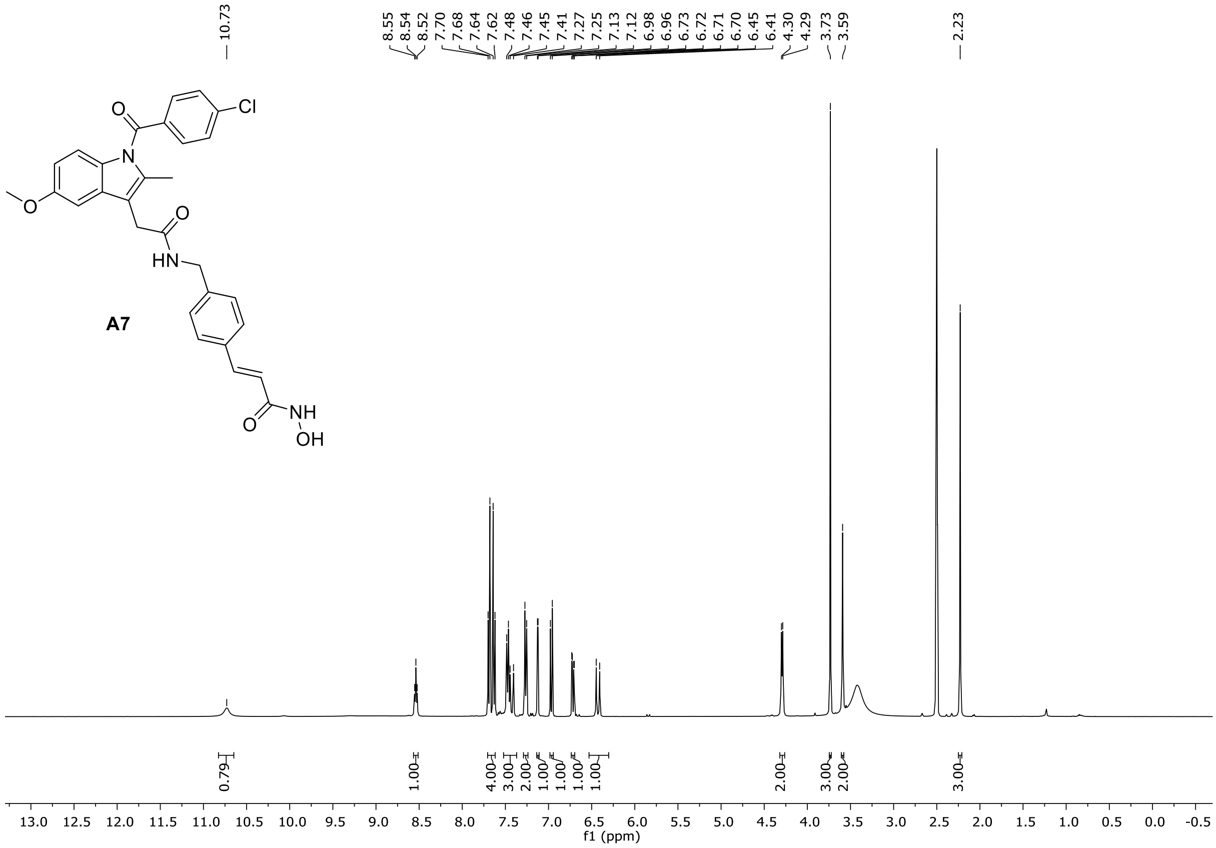

Figure S18. ^{1}H-NMR spectrum of **A7** in DMSO-d_6.

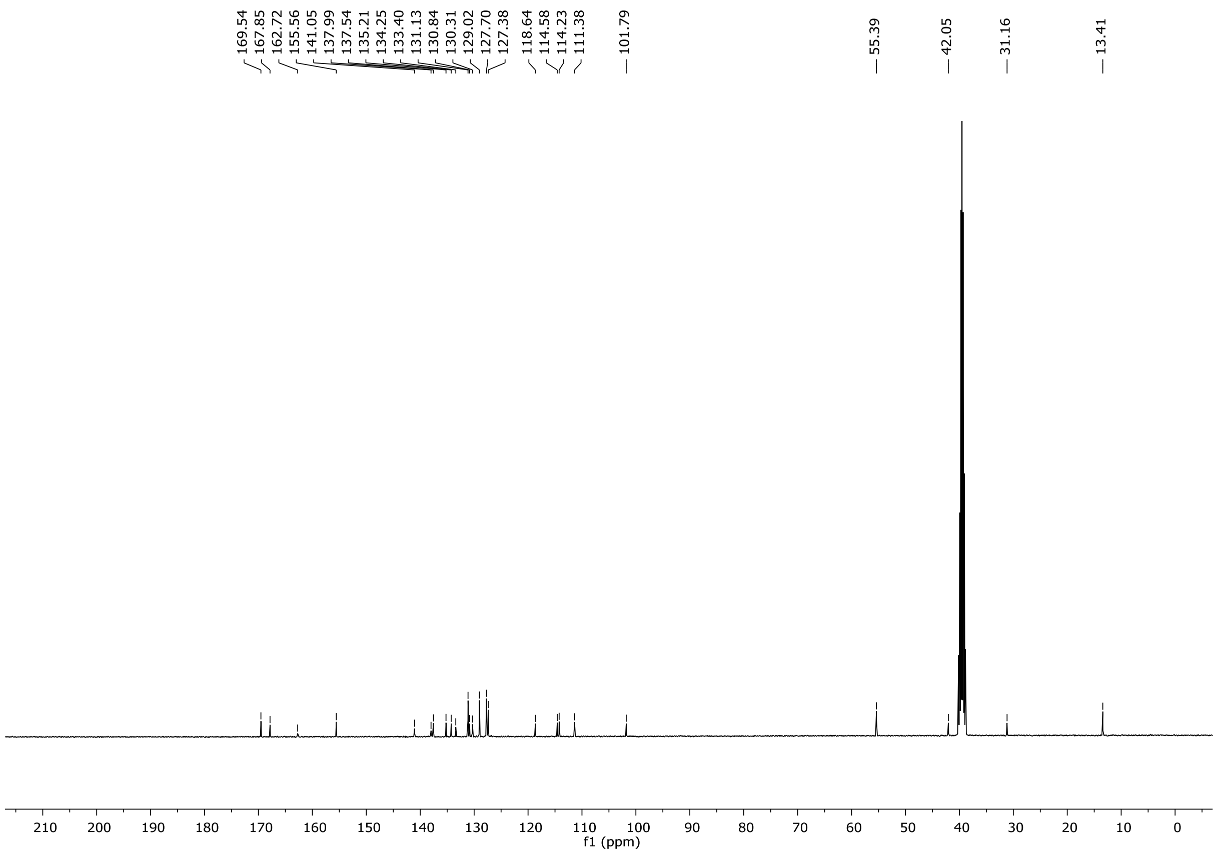

Figure S19. ^{13}C-NMR spectrum of **A7** in DMSO-d_6.

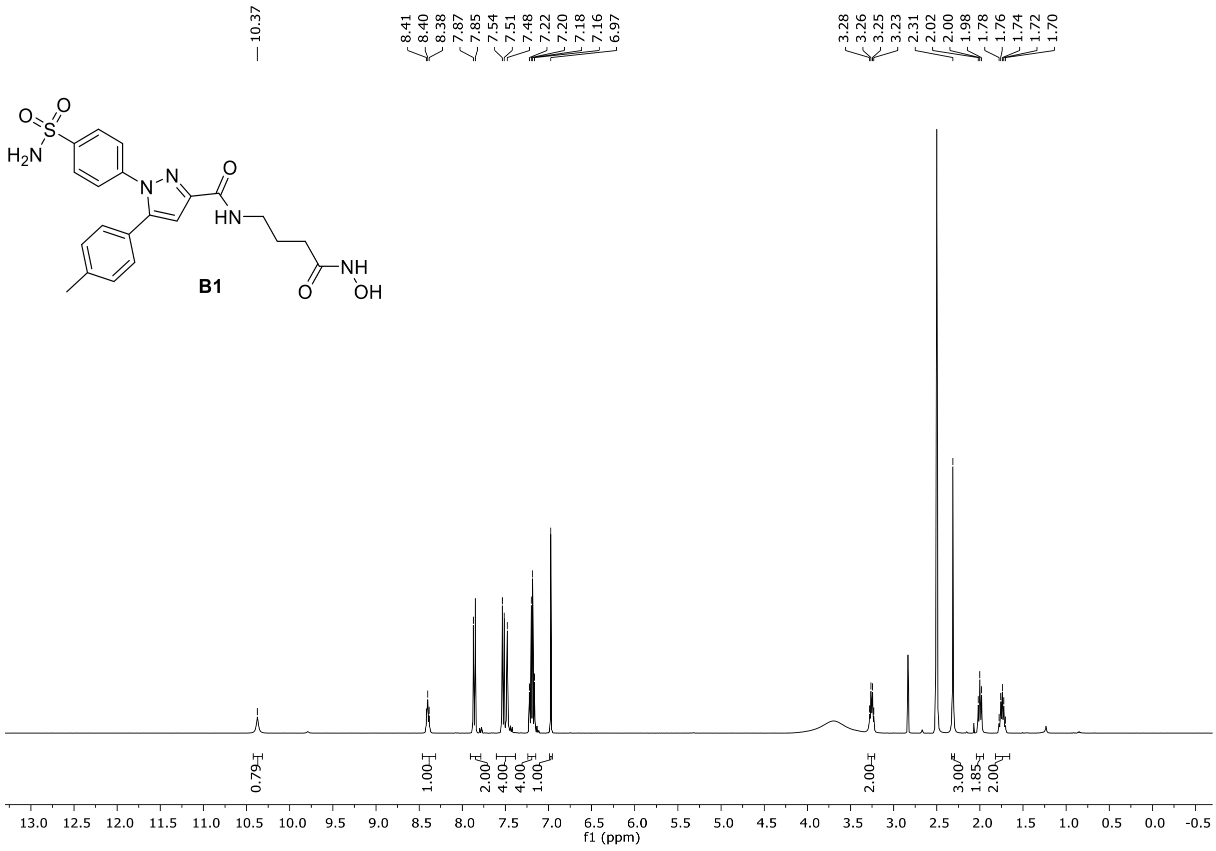

Figure S20. ^{1}H-NMR spectrum of **B1** in DMSO-d_6.

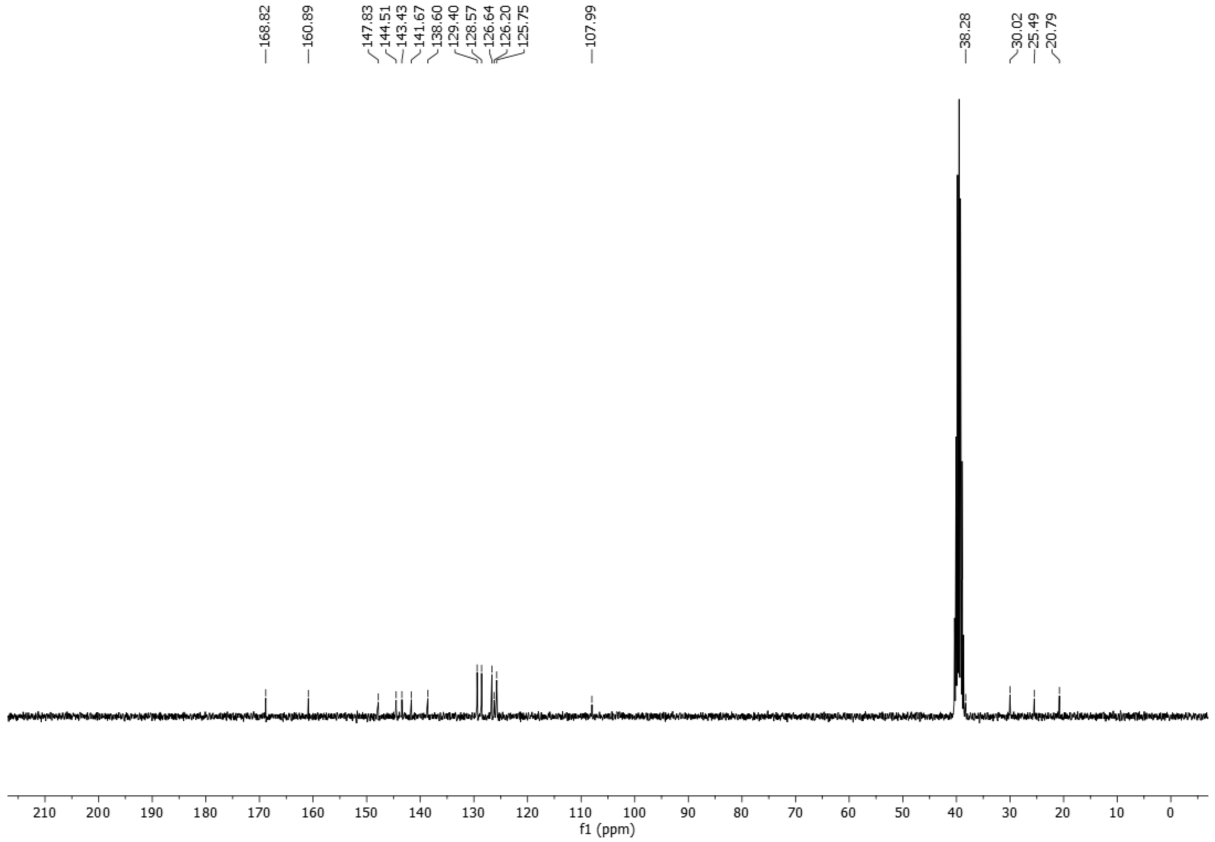

Figure S21. ^{13}C-NMR spectrum of **B1** in DMSO-d_6.

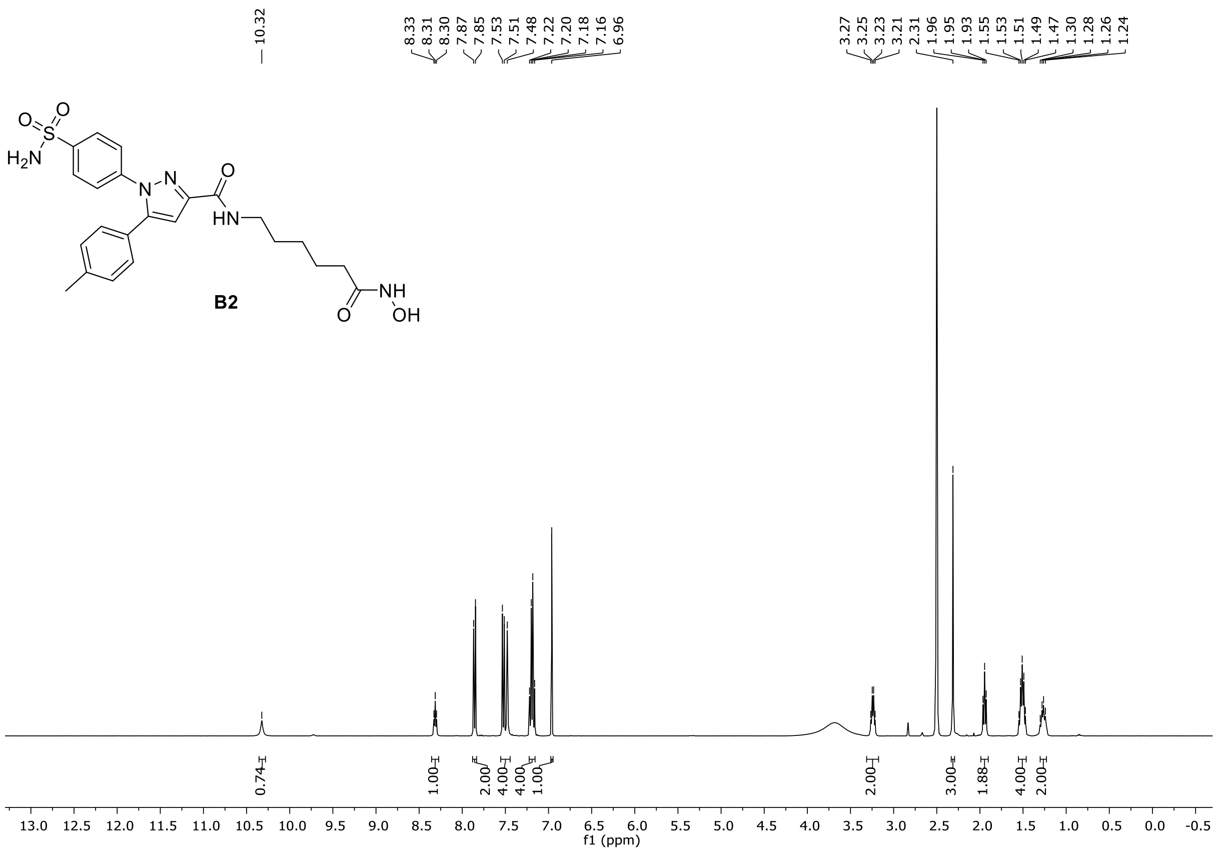

Figure S22. ^{1}H-NMR spectrum of **B2** in DMSO-d_6.

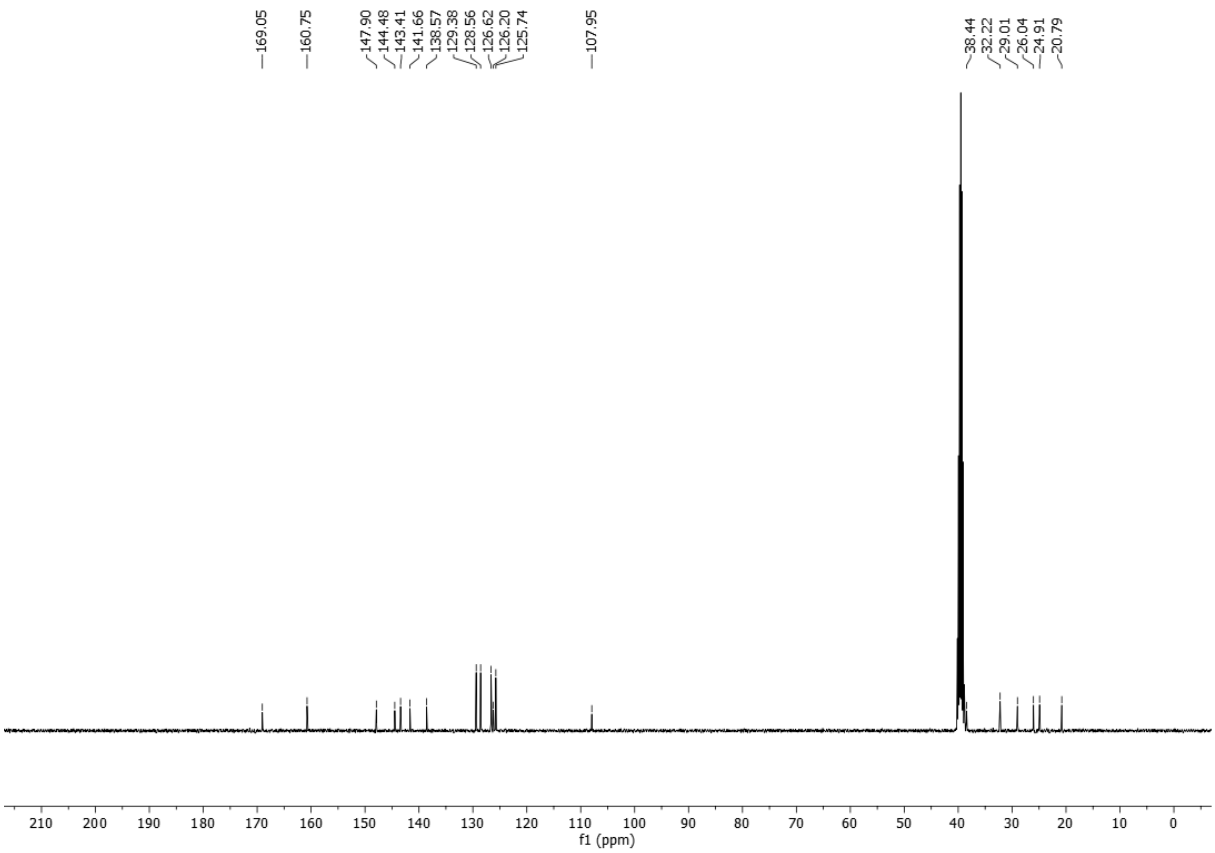

Figure S23. ^{13}C-NMR spectrum of **B2** in DMSO-*d*$_6$.

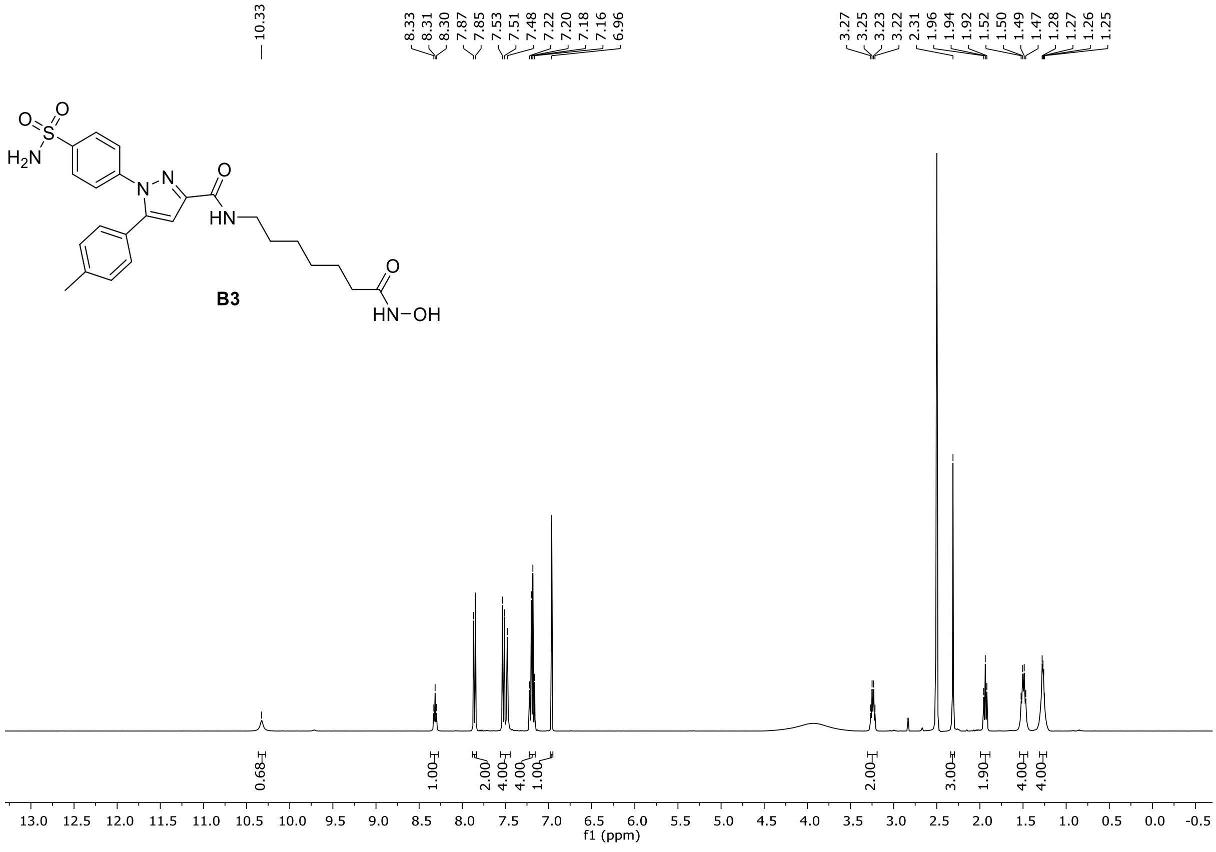

Figure S24. ^{1}H-NMR spectrum of **B3** in DMSO-*d*$_6$.

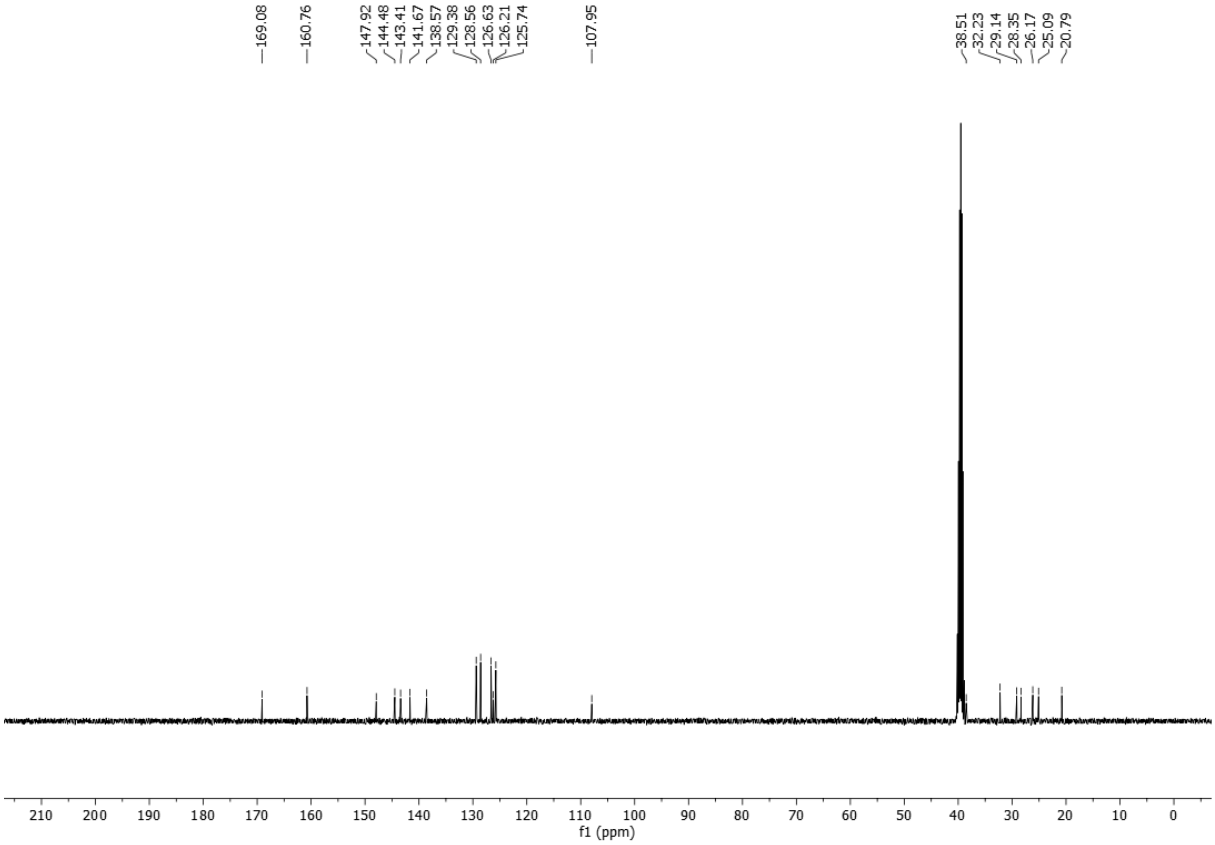

Figure S25. ^{13}C-NMR spectrum of **B3** in DMSO-d_6.

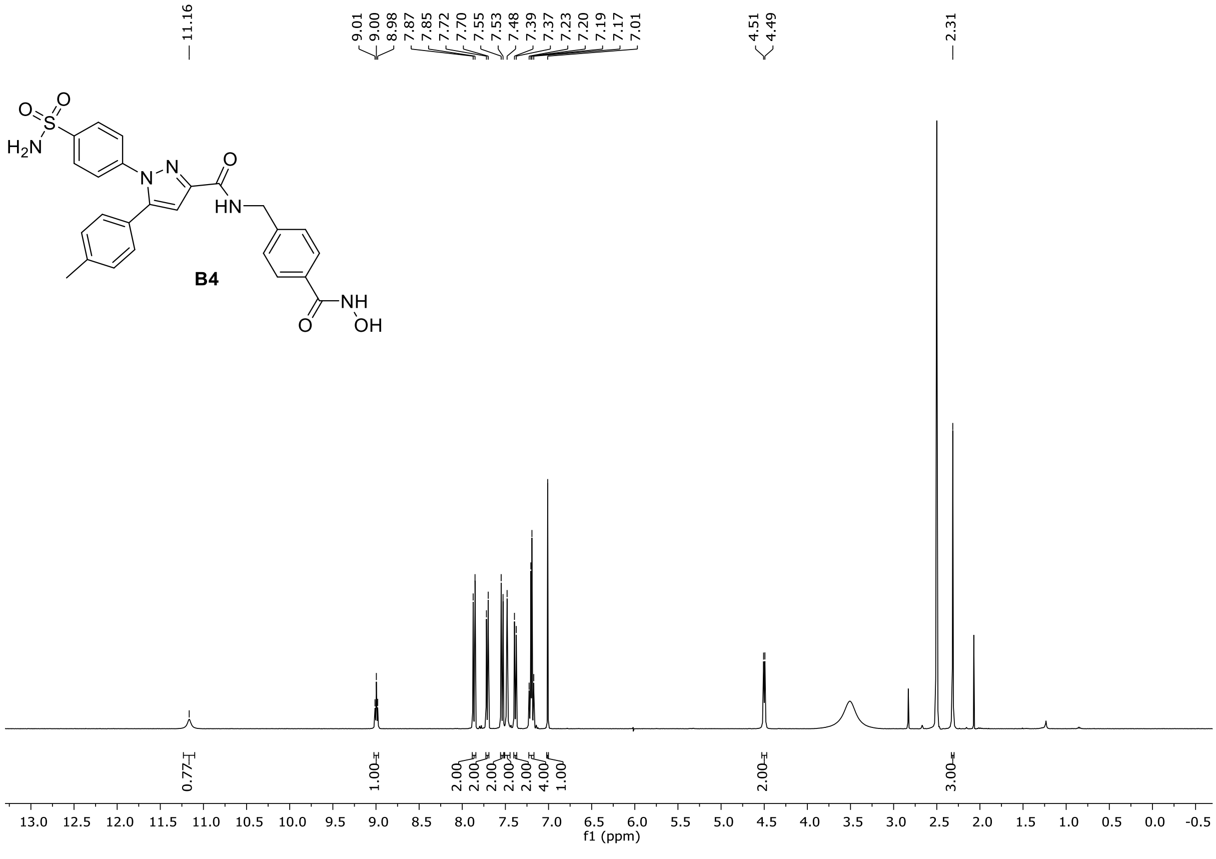

Figure S26. ^{1}H-NMR spectrum of **B4** in DMSO-d_6.

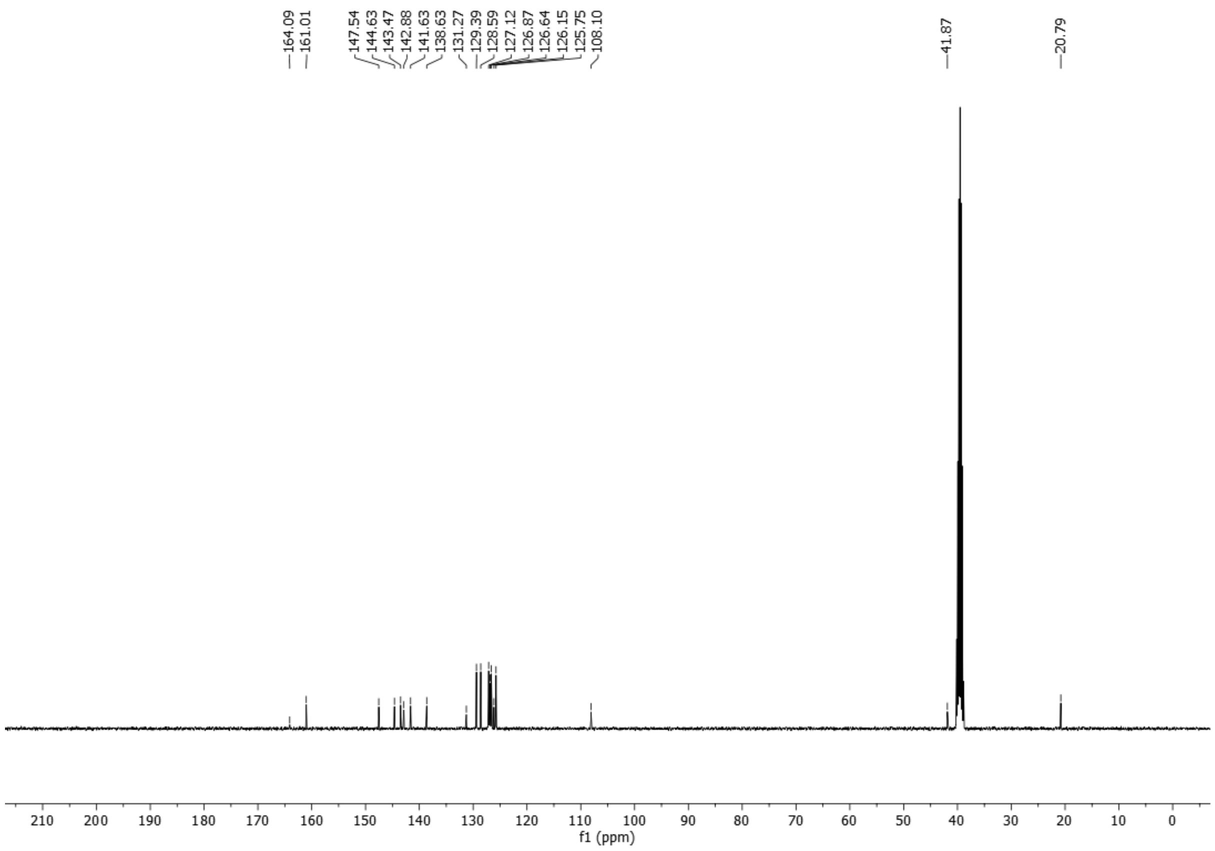

Figure S27. ^{13}C-NMR spectrum of **B4** in DMSO-d_6.

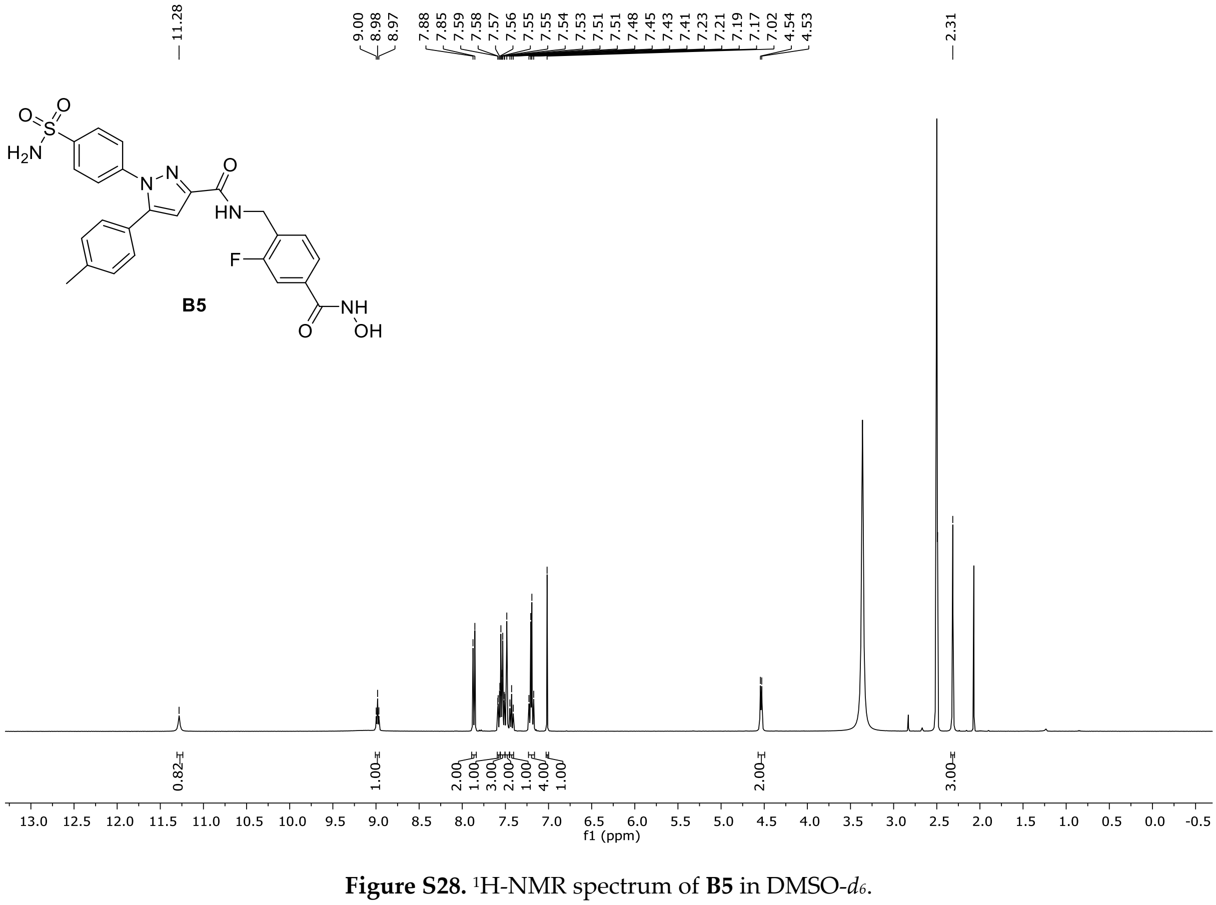

Figure S28. ^{1}H-NMR spectrum of **B5** in DMSO-d_6.

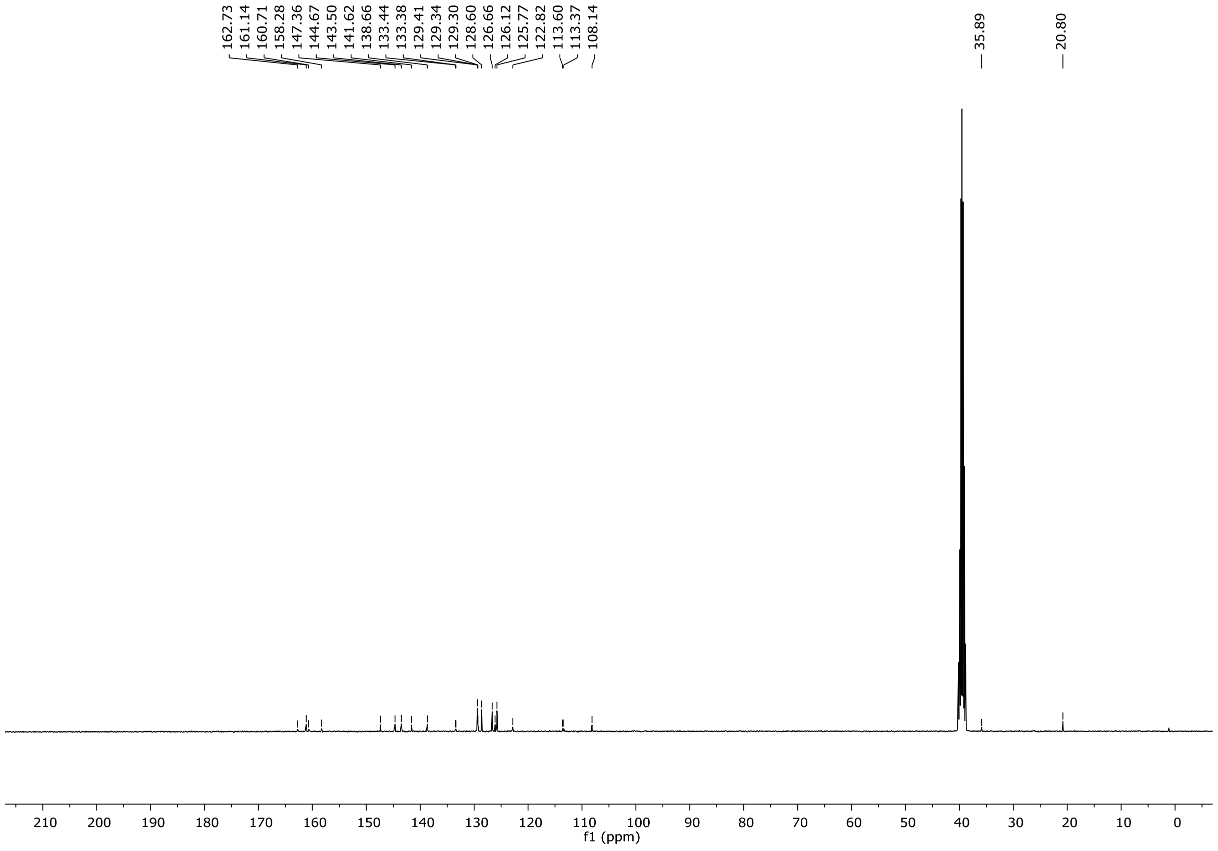

Figure S29. ^{13}C-NMR spectrum of **B5** in DMSO-*d*6.

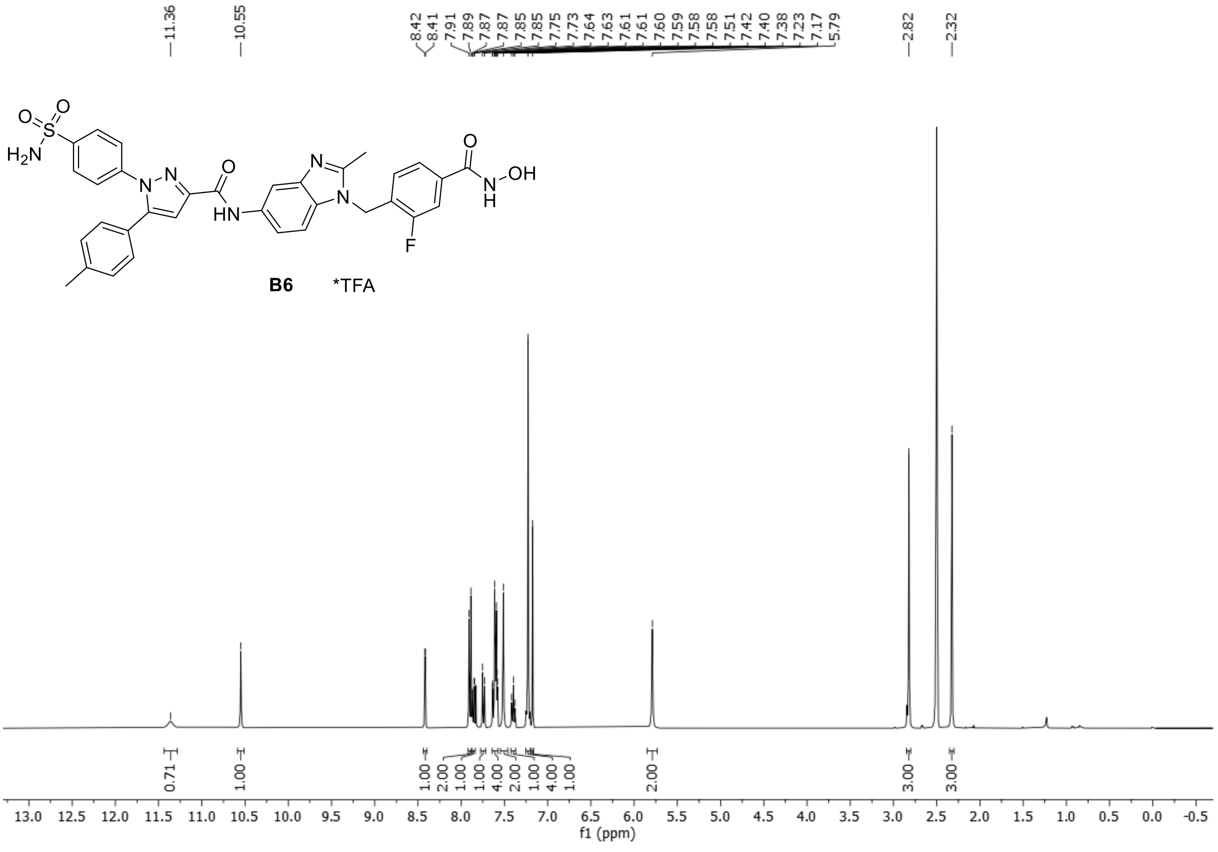

Figure S30. ^{1}H-NMR spectrum of **B6** in DMSO-*d*6.

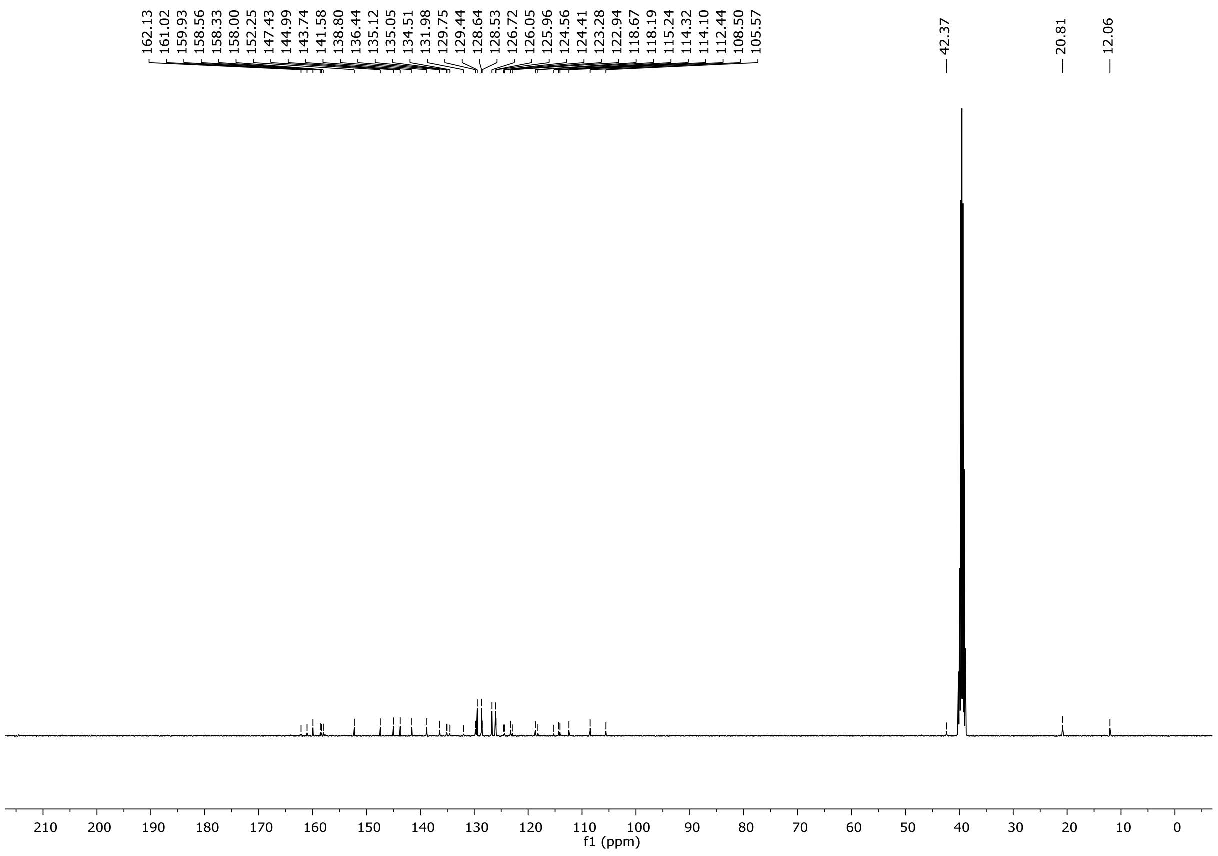

Figure S31. ^{13}C-NMR spectrum of **B6** in DMSO-d_6.

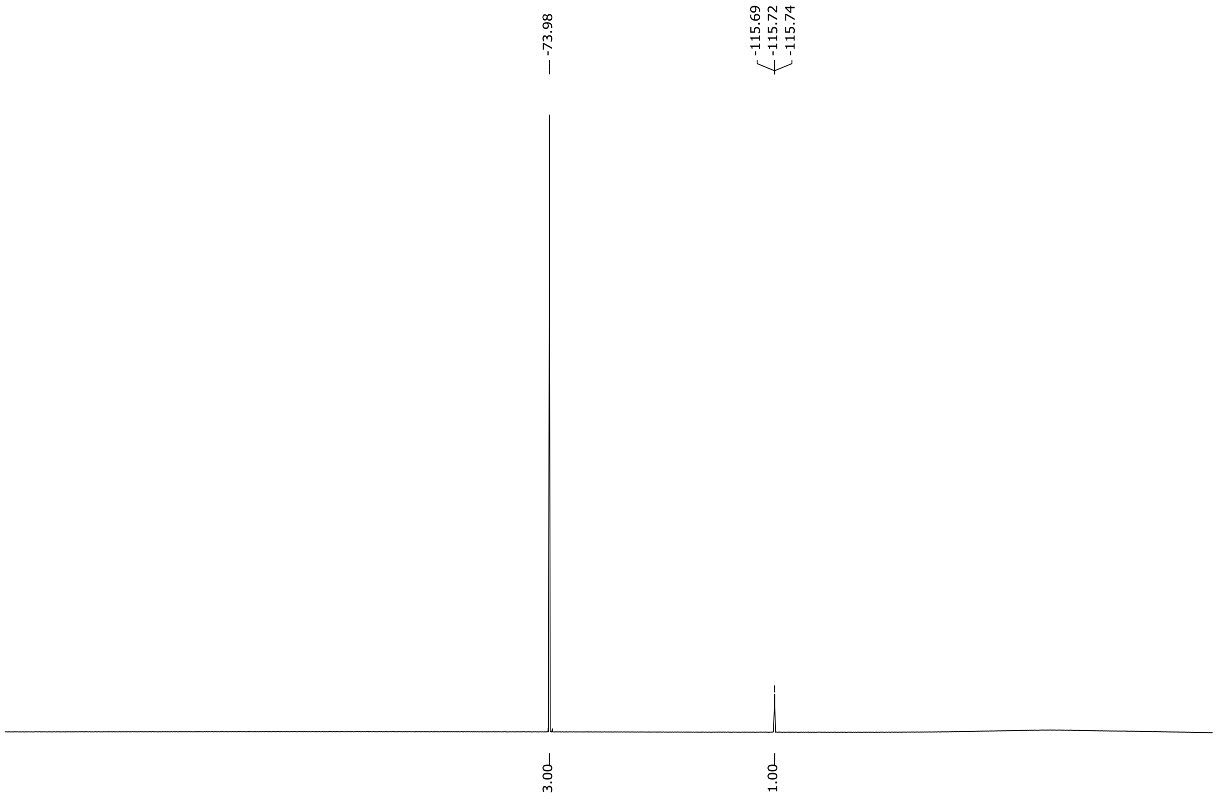

Figure S32. ^{19}F-NMR spectrum of **B6** in DMSO-d_6.

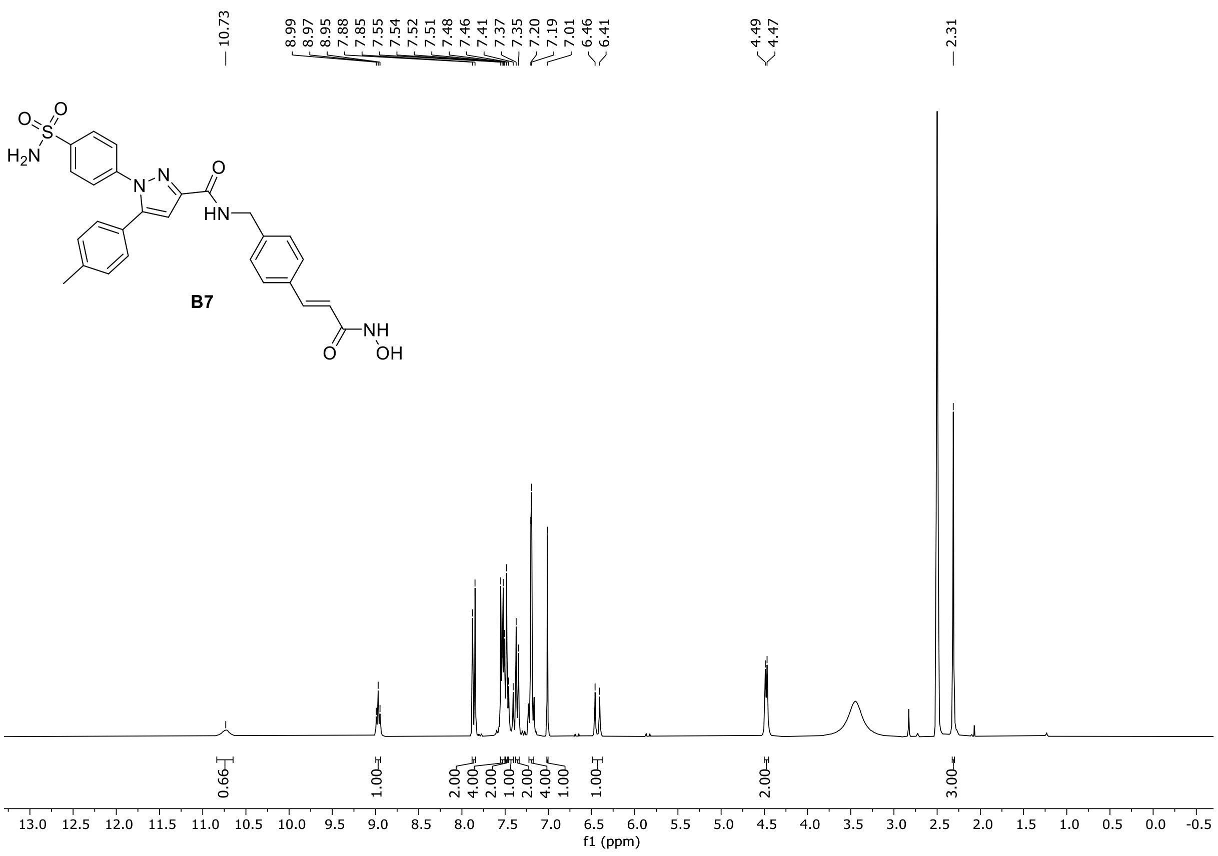

Figure S33. ^{1}H-NMR spectrum of **B7** in DMSO-d_6.

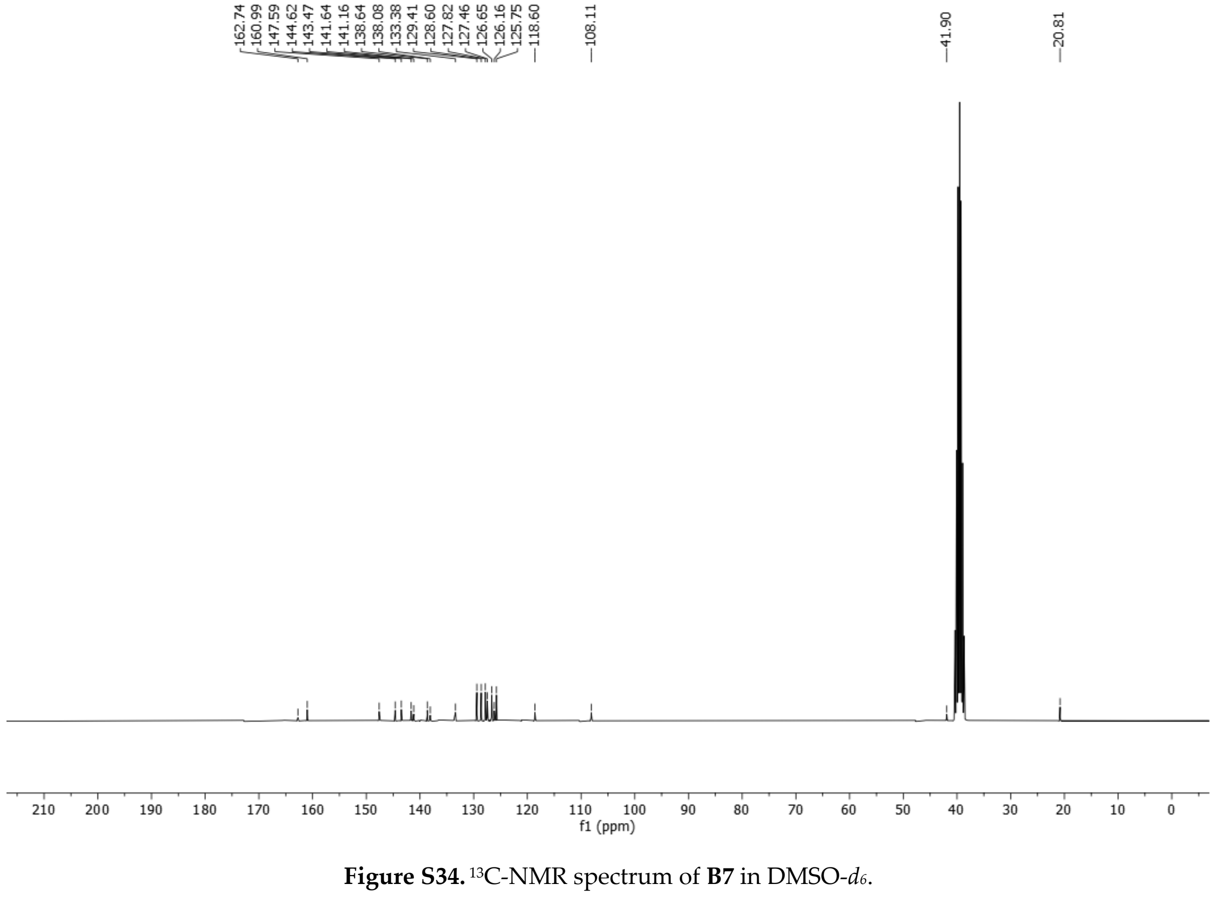

Figure S34. ^{13}C-NMR spectrum of **B7** in DMSO-d_6.

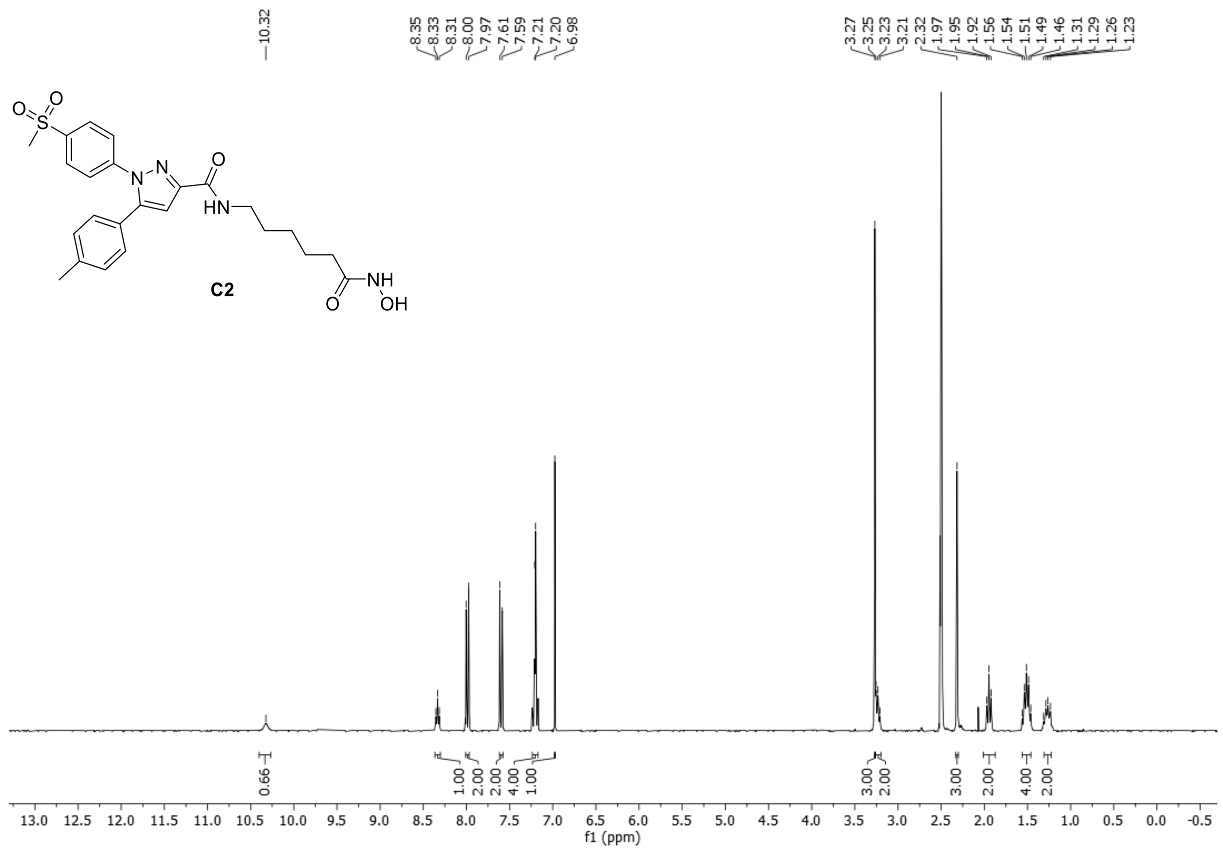

Figure S35. ^{1}H-NMR spectrum of **C2** in DMSO-d_6.

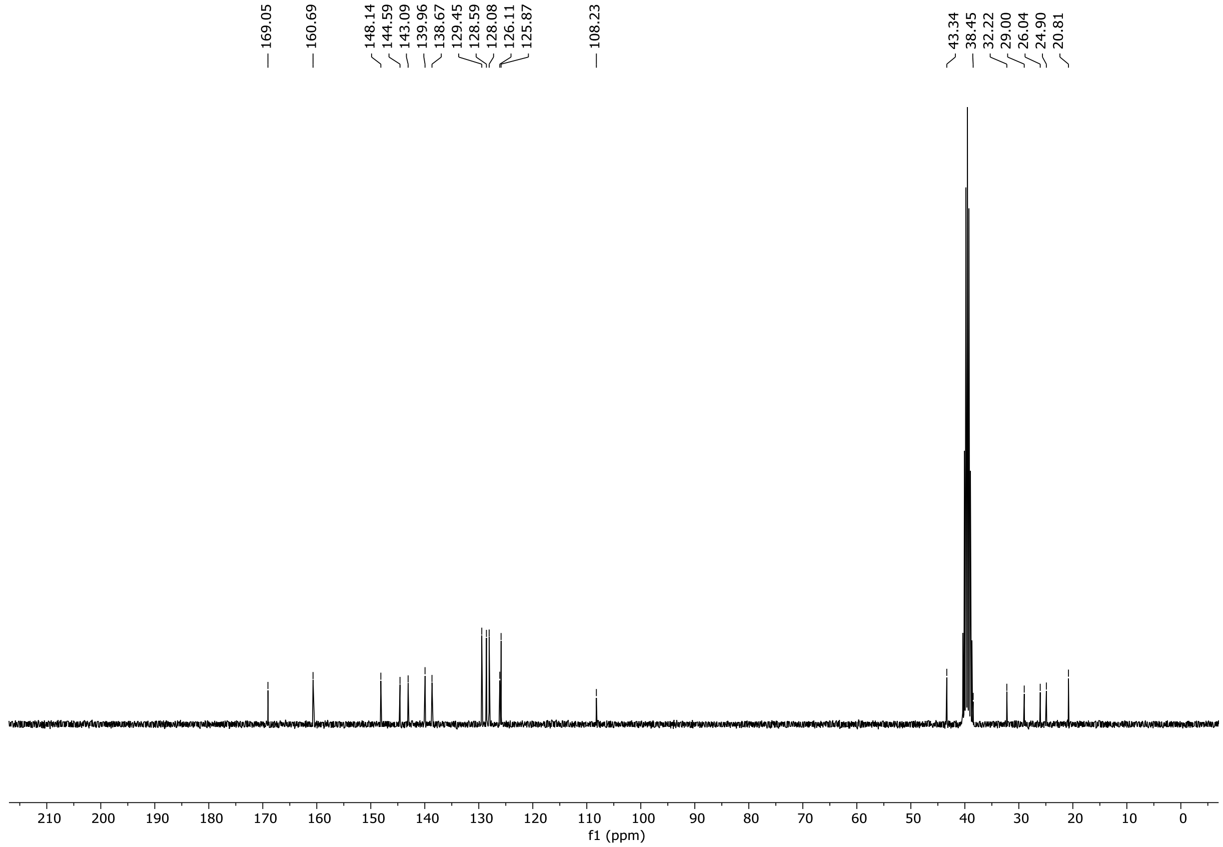

Figure S36. ^{13}C-NMR spectrum of **C2** in DMSO-d_6.

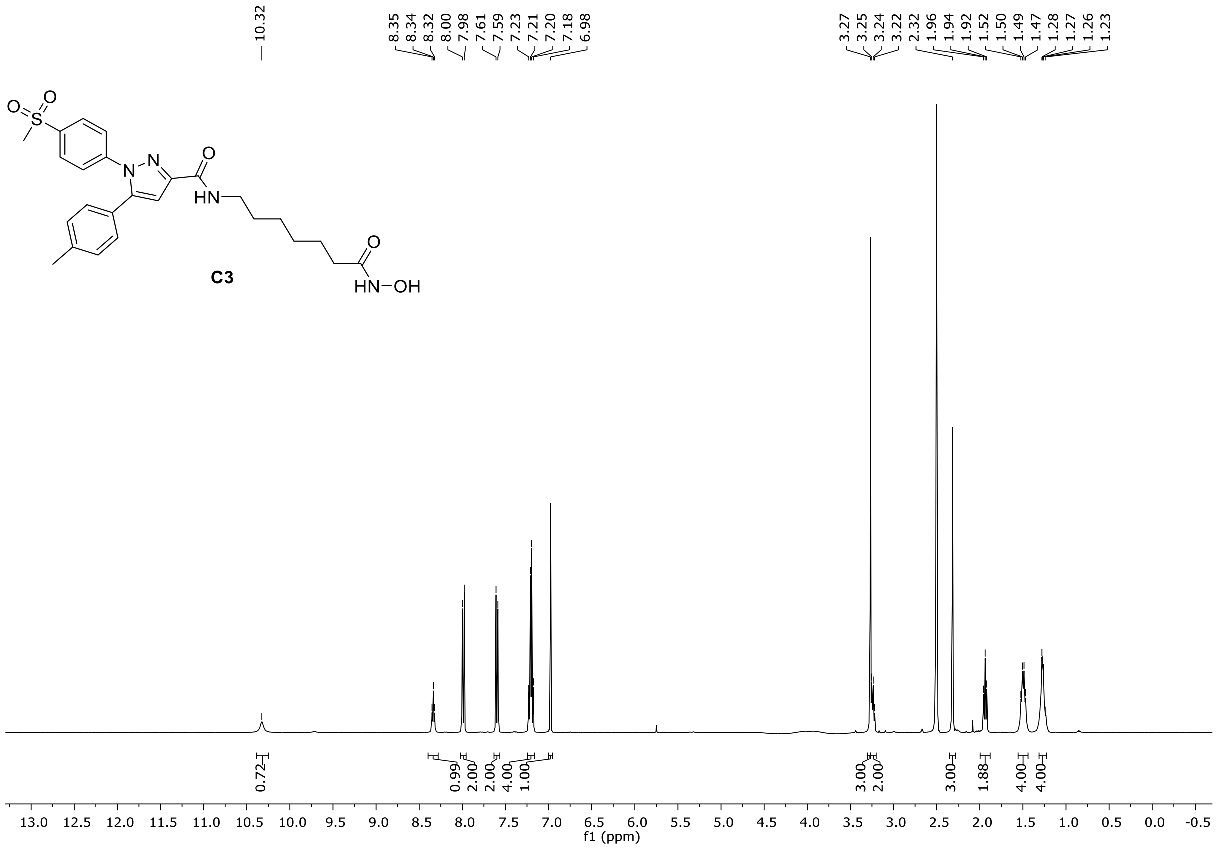

Figure S37. ^{1}H-NMR spectrum of **C3** in DMSO-d_6.

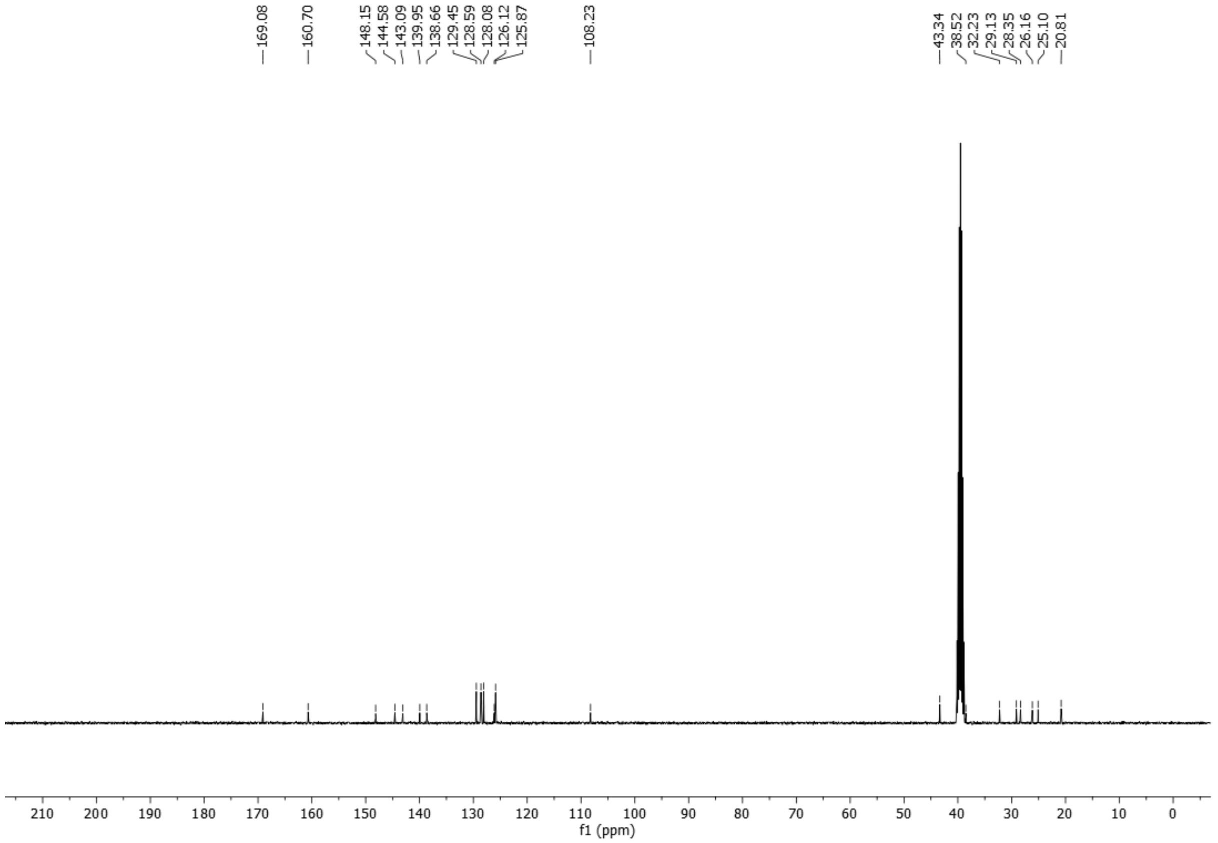

Figure S38. ^{13}C-NMR spectrum of **C3** in DMSO-d_6.

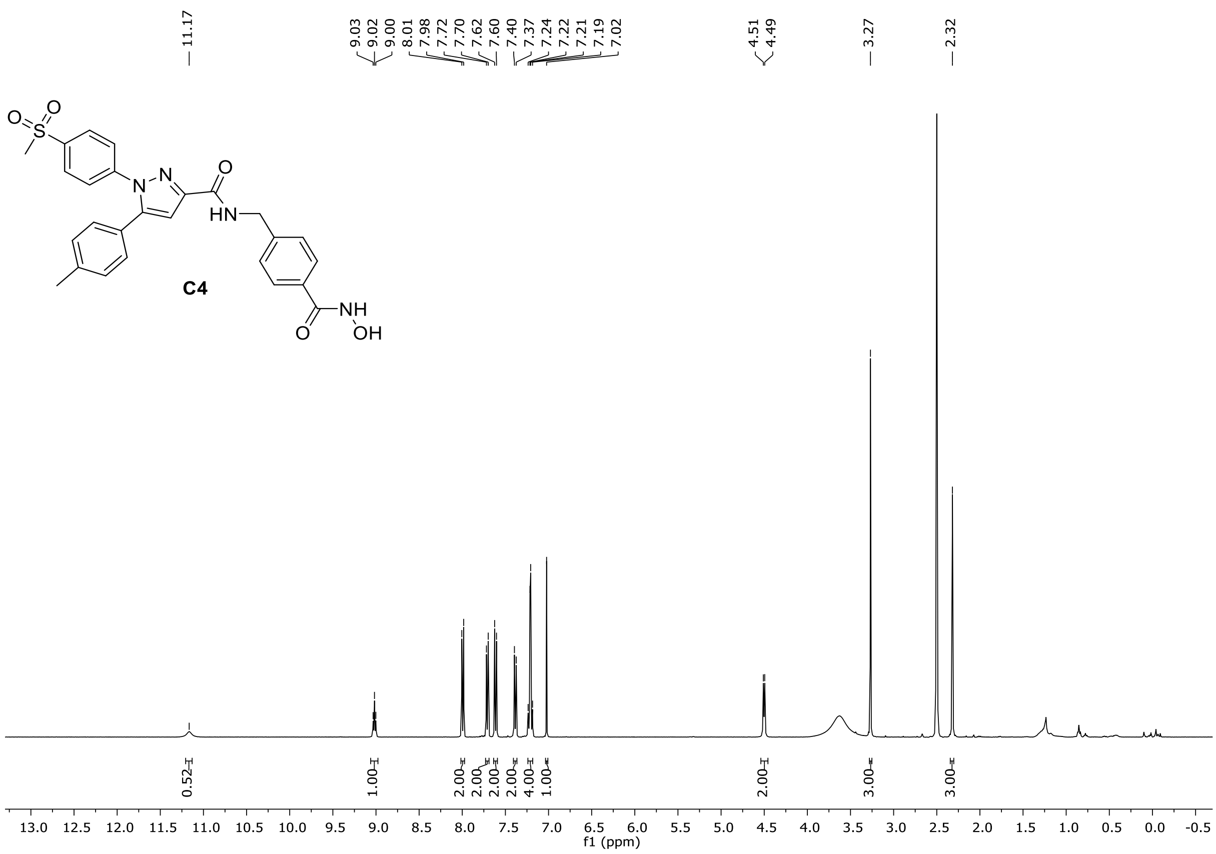

Figure S39. ^{1}H-NMR spectrum of **C4** in DMSO-d_6.

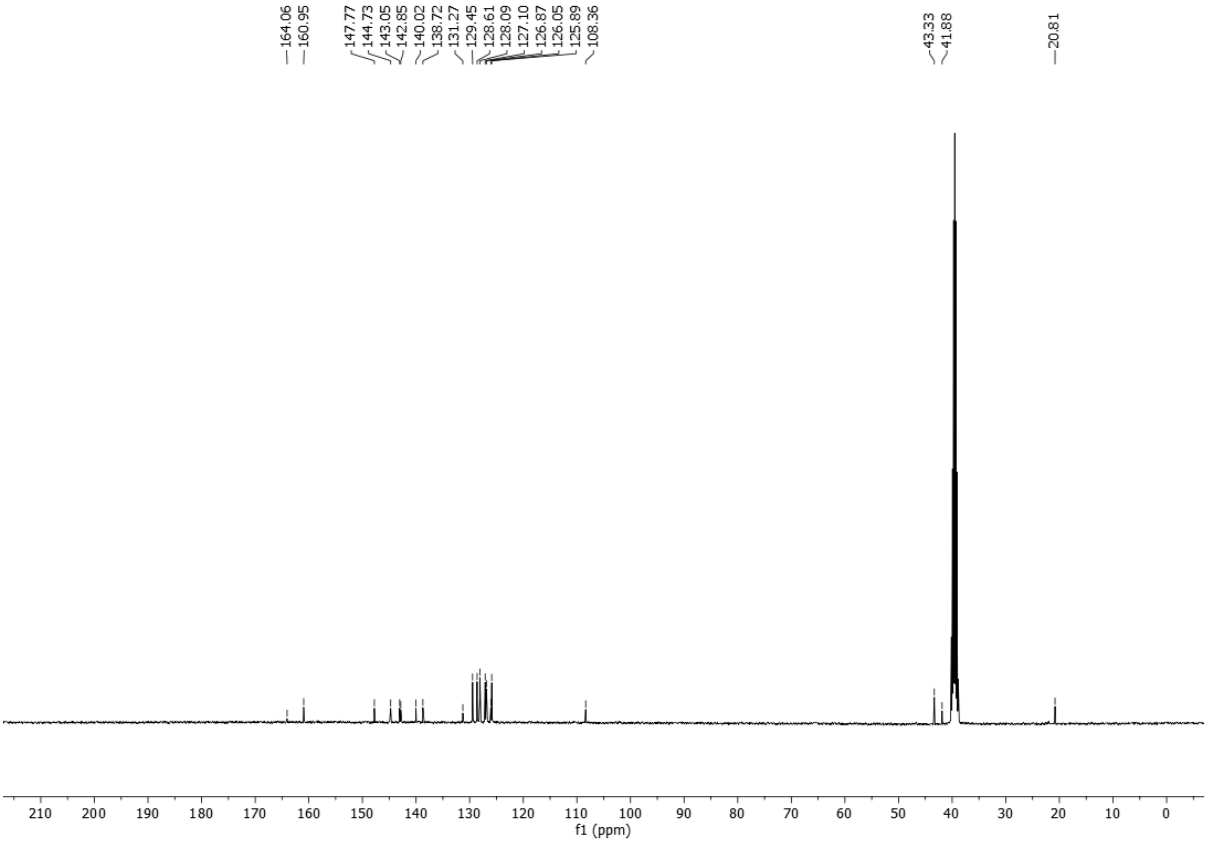

Figure S40. ^{13}C-NMR spectrum of **C4** in DMSO-d_6.

3. HPLC Chromatograms

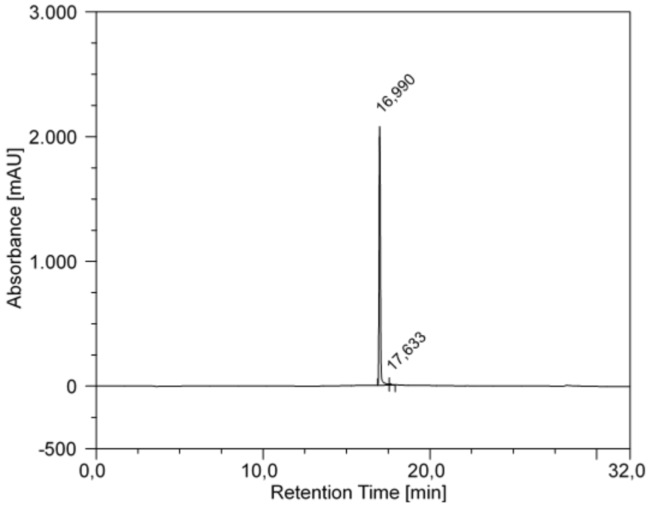

Sample	Ret.Time [min]	Height [mAU]	Area [mAU*min]	Rel.Area [%]
**A1**	16,99	2076,322	184,019	99,20

Figure S41. HPLC chromatogram of **A1** (purity: 99%).

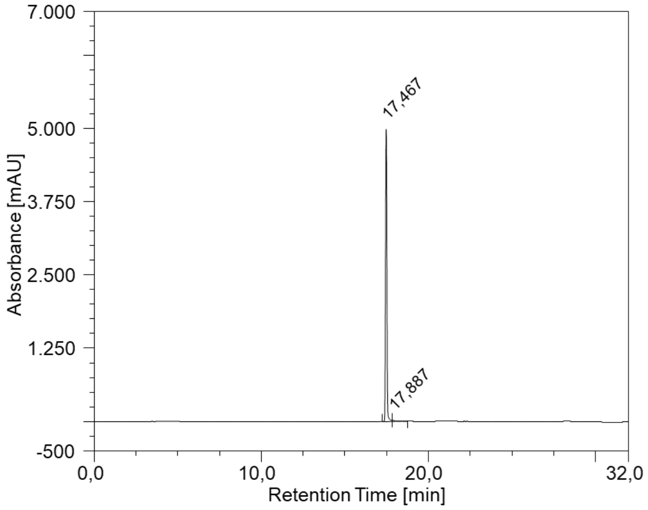

Sample	Ret.Time [min]	Height [mAU]	Area [mAU*min]	Rel.Area [%]
**A2**	17,47	4989,215	479,067	98,89

Figure S42. HPLC chromatogram of **A2** (purity: 99%).

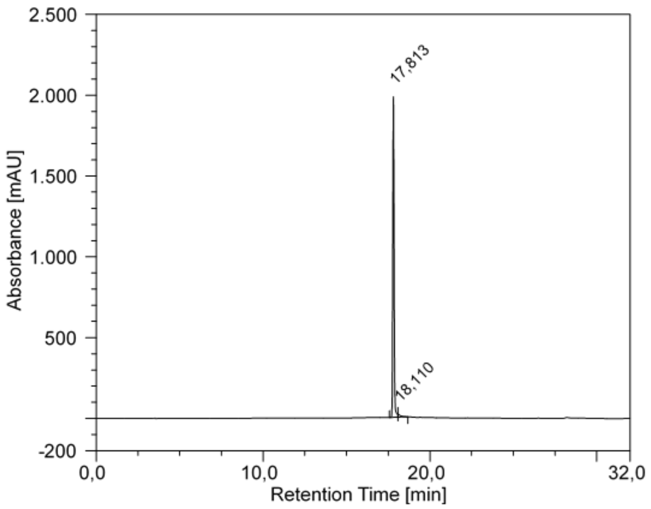

Sample	Ret.Time [min]	Height [mAU]	Area [mAU*min]	Rel.Area [%]
**A3**	17,81	1983,886	180,482	97,94

Figure S43. HPLC chromatogram of **A3** (purity: 98%).

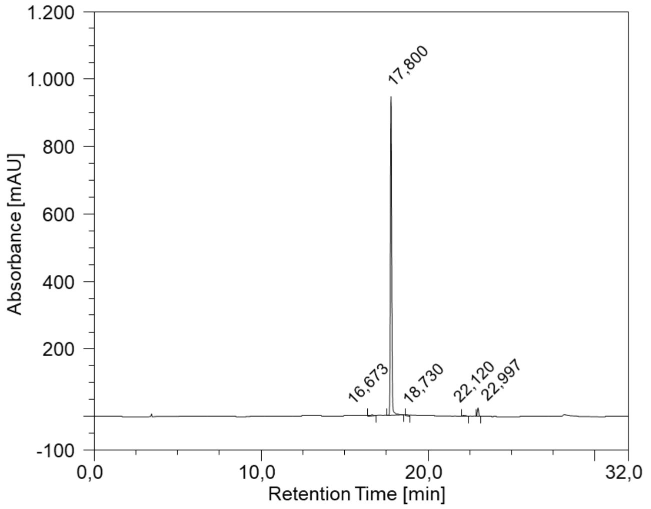

Sample	Ret.Time [min]	Height [mAU]	Area [mAU*min]	Rel.Area [%]
**A4**	17,80	945,577	85,966	96,56

Figure S44. HPLC chromatogram of **A4** (purity: 97%).

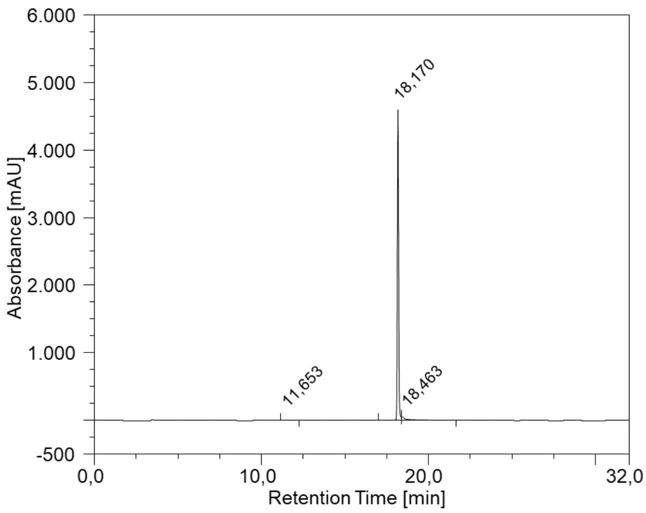

Sample	Ret.Time [min]	Height [mAU]	Area [mAU*min]	Rel.Area [%]
**A5**	18,17	4594,059	409,017	96,14

Figure S45. HPLC chromatogram of **A5** (purity: 96%).

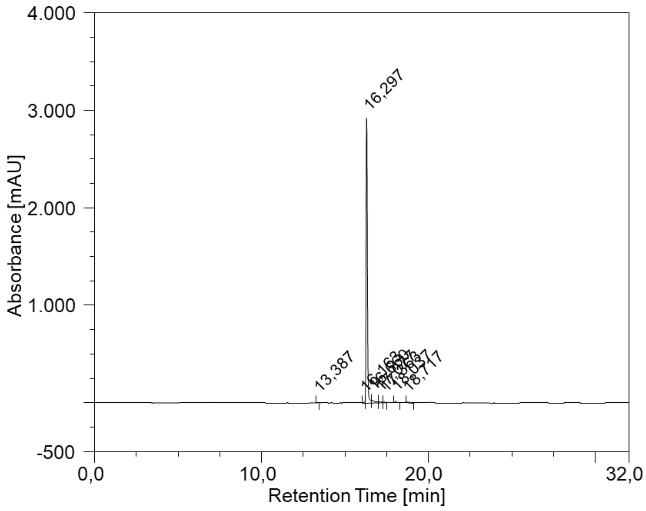

Sample	Ret.Time [min]	Height [mAU]	Area [mAU*min]	Rel.Area [%]
**A6**	16,30	2920,745	263,558	96,67

Figure S46. HPLC chromatogram of **A6** (purity: 97%).

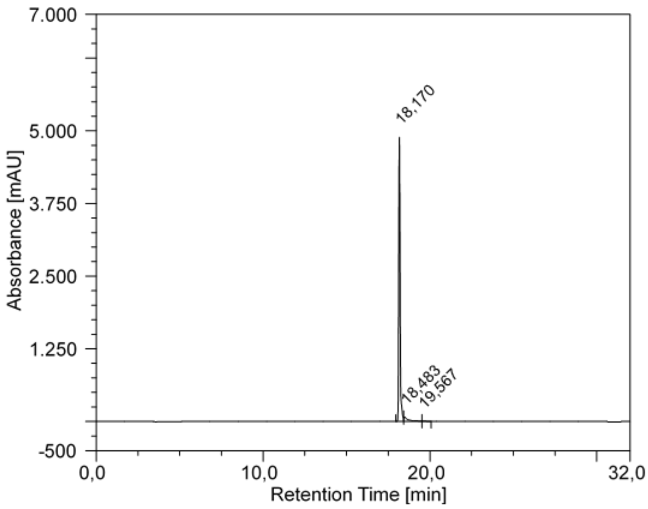

Sample	Ret.Time [min]	Height [mAU]	Area [mAU*min]	Rel.Area [%]
**A7**	18,17	4888,603	471,329	95,47

Figure S47. HPLC chromatogram of **A7** (purity: 95%).

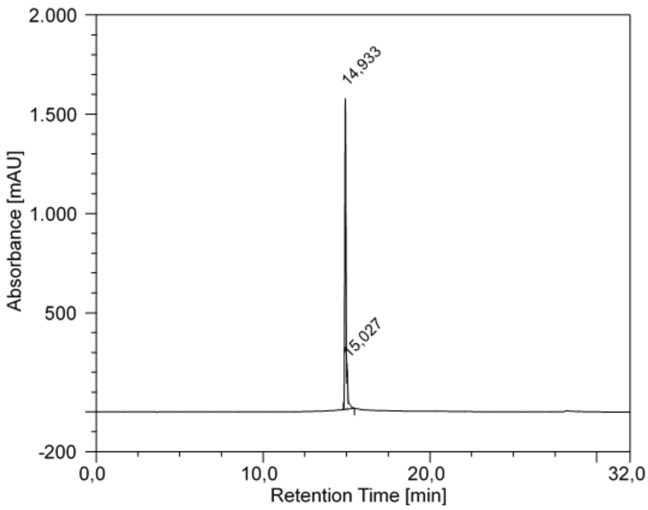

Sample	Ret.Time [min]	Height [mAU]	Area [mAU*min]	Rel.Area [%]
**B1**	14,93	1566,333	126,024	88,93

Figure S48. HPLC chromatogram of **B1** (purity: 89%).

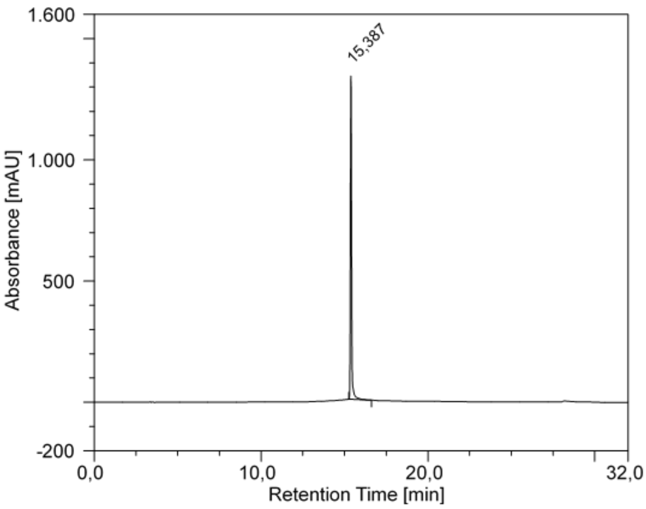

Sample	Ret.Time [min]	Height [mAU]	Area [mAU*min]	Rel.Area [%]
**B2**	15,39	1333,555	114,397	100,00

Figure S49. HPLC chromatogram of **B2** (purity: >99%).

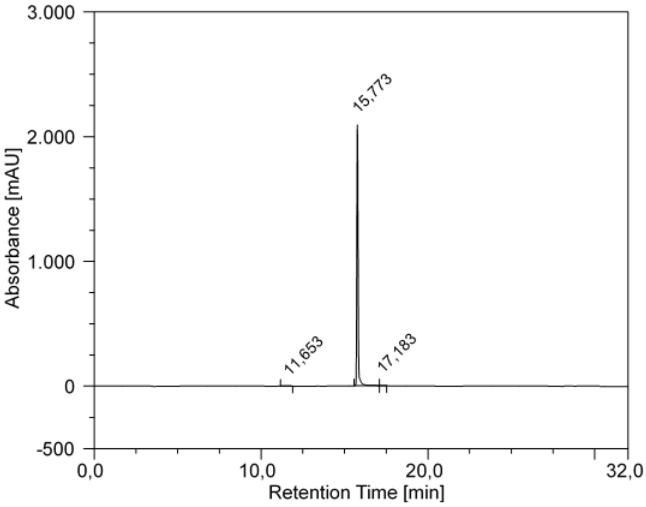

Sample	Ret.Time [min]	Height [mAU]	Area [mAU*min]	Rel.Area [%]
**B3**	15,77	2092,195	188,008	99,68

Figure S50. HPLC chromatogram of **B3** (purity: >99%).

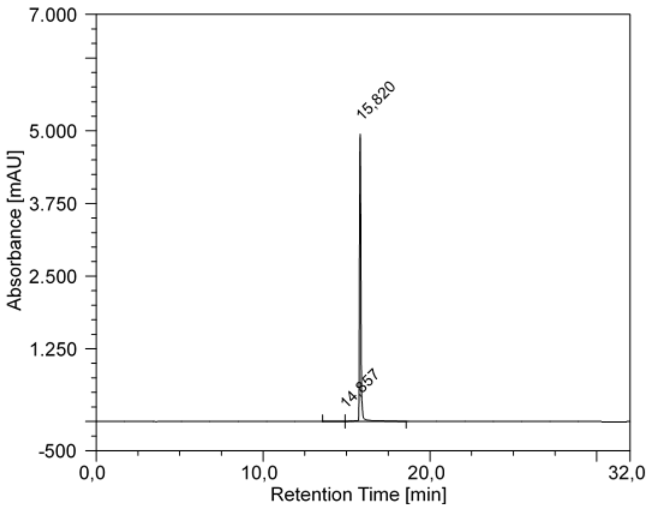

Sample	Ret.Time [min]	Height [mAU]	Area [mAU*min]	Rel.Area [%]
**B4**	15,82	4939,956	497,955	99,73

Figure S51. HPLC chromatogram of **B4** (purity: >99%).

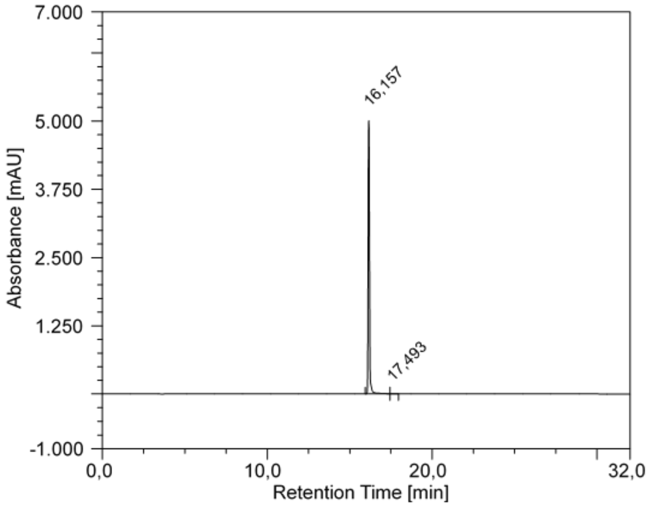

Sample	Ret.Time [min]	Height [mAU]	Area [mAU*min]	Rel.Area [%]
**B5**	16,16	5006,839	492,472	99,90

Figure S52. HPLC chromatogram of **B5** (purity: >99%).

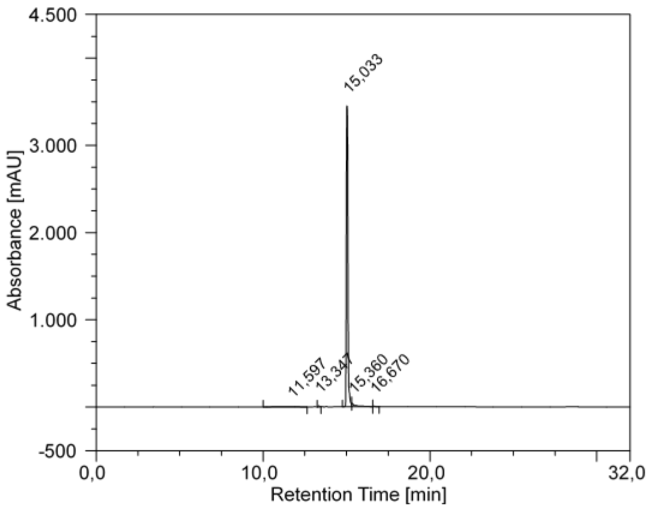

Sample	Ret.Time [min]	Height [mAU]	Area [mAU*min]	Rel.Area [%]
**B6**	15,03	3449,384	398,968	96,44

Figure S53. HPLC chromatogram of **B6** (purity: 96%).

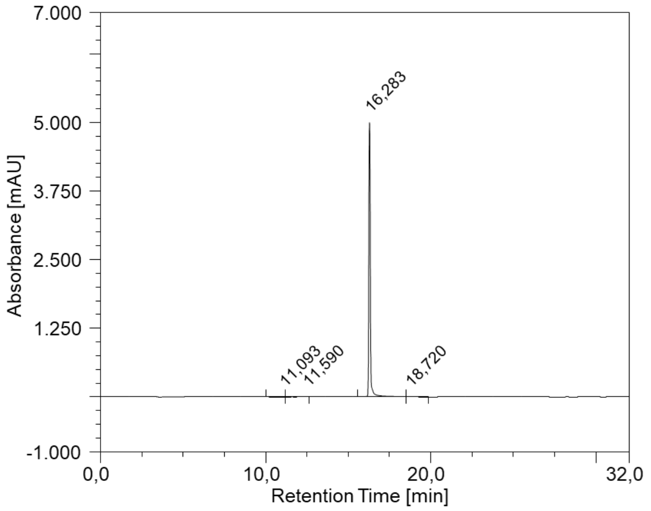

Sample	Ret.Time [min]	Height [mAU]	Area [mAU*min]	Rel.Area [%]
**B7**	16,28	4997,809	530,242	99,33

Figure S54. HPLC chromatogram of **B7** (purity: 99%).

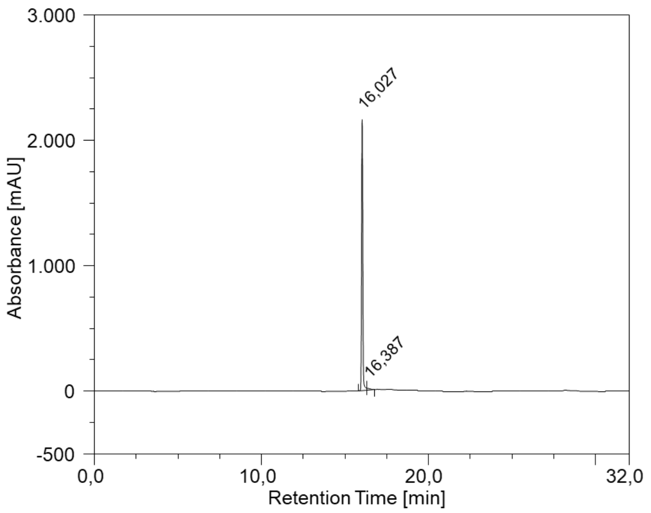

Sample	Ret.Time [min]	Height [mAU]	Area [mAU*min]	Rel.Area [%]
**C2**	16,03	2160,187	181,737	98,42

Figure S55. HPLC chromatogram of **C2** (purity: 98%).

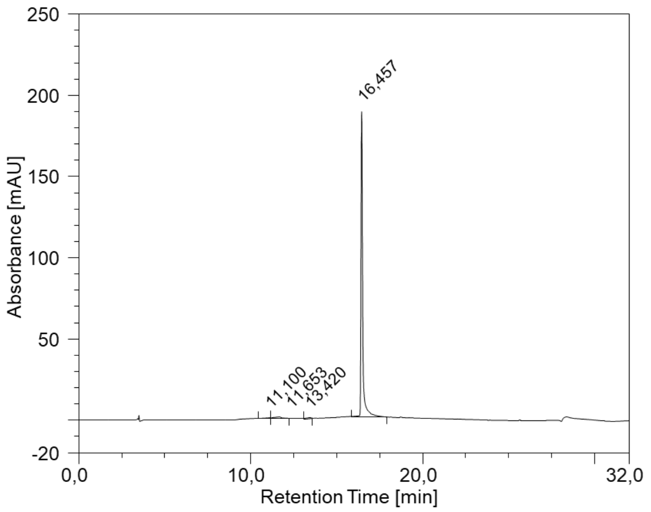

Sample	Ret.Time [min]	Height [mAU]	Area [mAU*min]	Rel.Area [%]
**C3**	16,46	187,911	21,539	96,52

Figure S56. HPLC chromatogram of **C3** (purity: 97%).

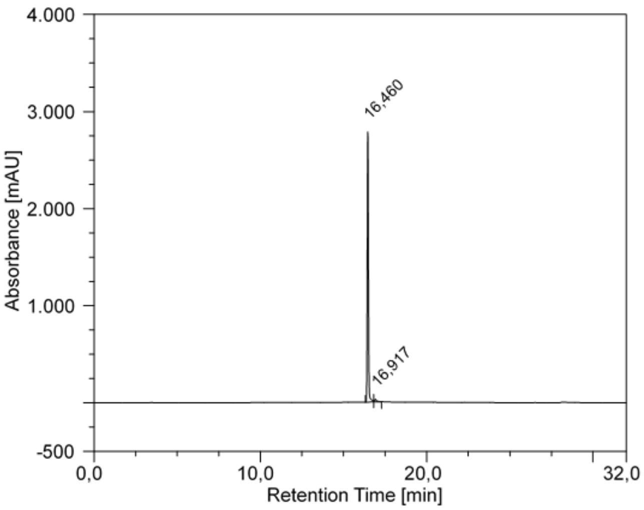

Sample	Ret.Time [min]	Height [mAU]	Area [mAU*min]	Rel.Area [%]
**C4**	16,46	2786,346	232,502	98,18

Figure S57. HPLC chromatogram of **C4** (purity: 98%).
